# High-Resolution AMS Dating of Architecture, Boulder Artworks and the Transition to Farming at Lepenski Vir

**DOI:** 10.1038/s41598-018-31884-7

**Published:** 2018-09-21

**Authors:** Dušan Borić, Thomas Higham, Emanuela Cristiani, Vesna Dimitrijević, Olaf Nehlich, Seren Griffiths, Craig Alexander, Bojana Mihailović, Dragana Filipović, Ethel Allué, Michael Buckley

**Affiliations:** 1 The Italian Academy for Advanced Studies in America, Columbia University, 1161 Amsterdam Avenue, New York, NY 10027 USA; 2 Oxford Radiocarbon Accelerator Unit, RLAHA, University of Oxford, Dyson Perrins Building, Oxford, OX1 3QY UK; 3 DANTE Diet and Ancient Technology Laboratory, Sapienza University of Rome, Via Caserta 6, 00161 Rome, Italy; 4 Department of Archaeology, University of Belgrade, Čika Ljubina 18–20, 11000 Belgrade, Serbia; 5 Department of Anthropology, University of British Columbia, 6303 NW Marine Drive, Vancouver, BC V6T 1Z1 Canada; 6 The School of Forensic and Applied Sciences, University of Central Lancashire, Preston, Lancashire PR1 2HE UK; 7 Independent Researcher, Brescia, Italy; 8 National Museum, Trg Republike 1, 11000 Belgrade, Serbia; 9 Institute for Balkan Studies, Serbian Academy of Sciences and Arts, Knez Mihailova 45, 11000 Belgrade, Serbia; 10 Institut Català de Paleoecologia Humana i Evolució Social (IPHES), Campus Sescelades URV (Edifici W3), 43007 Tarragona, Spain; 11 Àrea de Prehistòria, Universitat Rovira i Virgili (URV), Av. Catalunya 35, 43002 Tarragona, Spain; 12 Manchester Institute for Biotechnology, The University of Manchester, 131 Princess Street, Manchester, M1 7DN UK

## Abstract

The archaeological site of Lepenski Vir is widely known after its remarkable stone art sculptures that represent a unique and unprecedented case of Holocene hunter-gatherer creativity. These artworks were found largely associated with equally unique trapezoidal limestone building floors around their centrally located rectangular stone-lined hearths. A debate has raged since the discovery of the site about the chronological place of various discovered features. While over years different views from that of the excavator about the stratigraphy and chronology of the site have been put forward, some major disagreements about the chronological position of the features that make this site a key point of reference in European Prehistory persist. Despite challenges of re-analyzing the site’s stratigraphy from the original excavation records, taphonomic problems, and issues of reservoir offsets when providing radiocarbon measurements on human and dog bones, our targeted AMS (Accelerator Mass Spectrometry) dating of various contexts from this site with the application of Bayesian statistical modelling allows us to propose with confidence a new and sound chronological framework and provide formal estimates for several key developments represented in the archaeological record of Lepenski Vir that help us in understanding the transition of last foragers to first farmers in southeast Europe as a whole.

## Introduction

**Figure 1:**
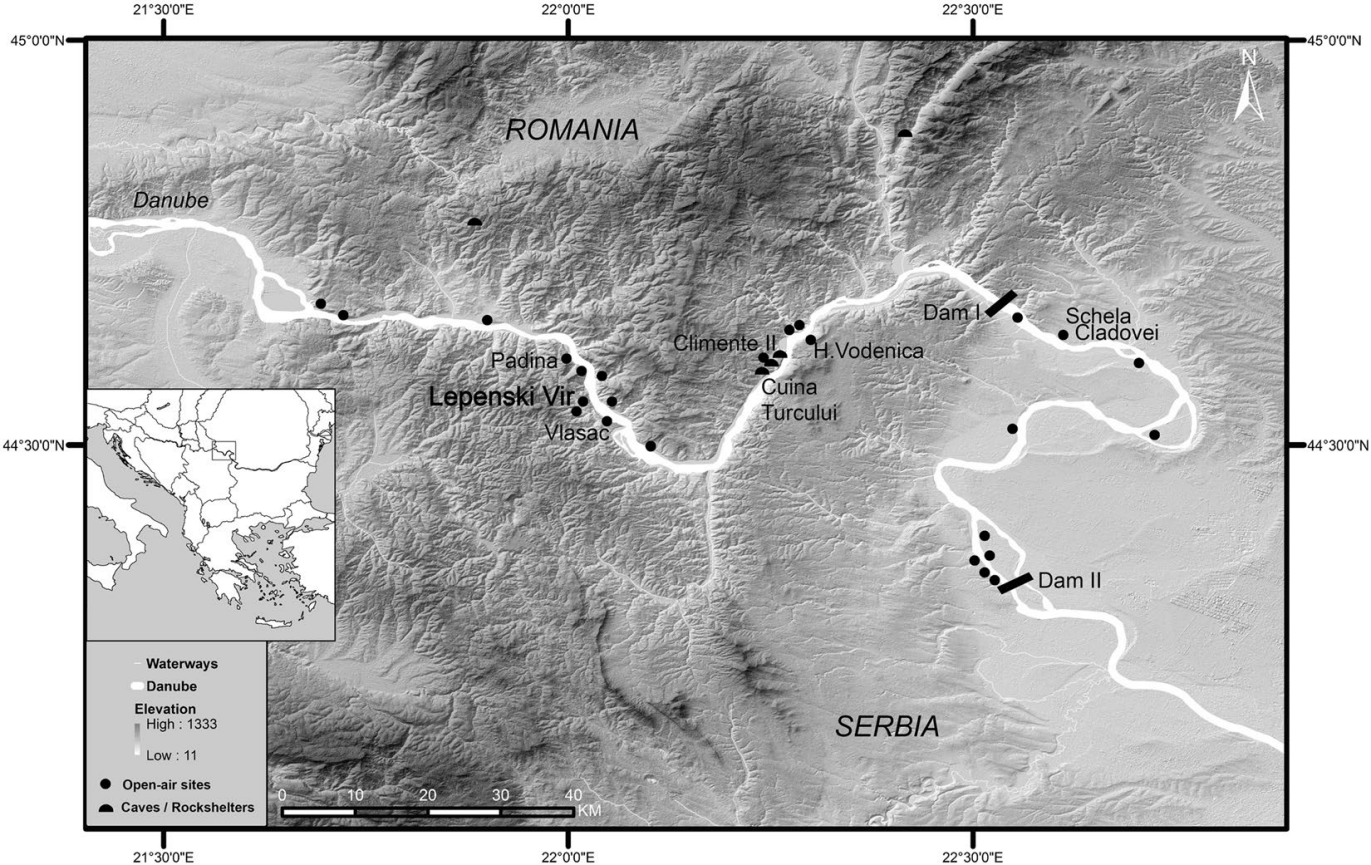
Map of the Danube Gorges area showing Lepenski Vir and other Mesolithic and Neolithic sites along the Danube. Base map elevation data source: ASTER GDEM (“ASTER GDEM is a product of METI and NASA”) courtesy NASA/JPL-Caltech. Figure prepared by D. Borić.

Lepenski Vir (translated from Serbian “Lepenski Whirlpool”). The site (44°33′N, 22°01′E) is situated on a Danube’s river terrace of the Lady’s Whirlpool Gorge of the Danube Gorges, also known as the Iron Gates region, of the north-central Balkans, 14 km upstream from the present-day town of Donji Milanovac. It is located at the elevation between 59 and 72 masl and the river terrace consists of finely laminated riverine sands and colluvial deposits. Excavations at the site took place from 1965 until 1970 and 2400 m^2^ were investigated, exposing the complete Mesolithic-Neolithic settlement spread^1,2^. Upon its discovery, the site became the type-site of a new, pre-Neolithic (Epipalaeolithic and Mesolithic) forager archaeological culture phenomenon that was limited to this region and found at another 20 sites along around 150 km stretch of the River Danube on both Serbian and Romanian banks (Fig. 1).

The region forms a specific micro zone with four gorges and three river valleys characterized by a complex geological history^3^. Here, the Danube cuts through the southern arm of the Carpathian Mountains’ range. Differential erosion of underlying rocks created irregularities in the riverbed that enabled the creation of large whirlpools and cataracts in many places. Humans effectively utilized these natural affordances for fishing at least since the beginning of the Holocene. Many places along the Danube where Mesolithic and Neolithic sites are found were the best locations for specialized fishing on whirlpools also in later times, as reflected in place naming^4^. The irregularities of the riverbed especially fostered sturgeon (Acipenseridae) fishing^5^, with scale bones of starlet and beluga species found in Mesolithic and Neolithic deposits of several sites in this region^6,7^. All these aspects of local ecology contributed to the development of long lasting forager adaptations with recorded stratigraphies reaching back to the Epipalaeolithic, which is dated to the Bølling–Allerød interstadial at Cuina Turcului rockshelter^8^ and Climente II Cave^9^. Several open-air locations, including Lepenski Vir, started being occupied from the beginning of the Holocene, c. 9700 cal BC.

**Figure 2:**
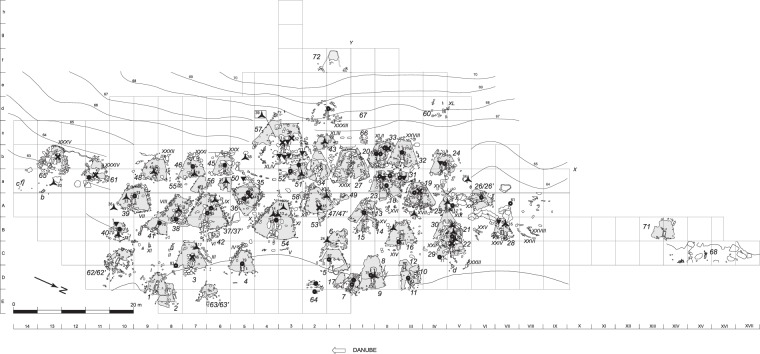
Plan of Lepenski Vir with phase I-II trapezoidal building features and distribution of sandstone boulders (see Supplementary Table S3 for the catalogue of boulders that corresponds to numbers associated with each sign). Key: triangles: depiction of hybrid human-fish faces; stars: geometric patterns over boulder surfaces; x: ‘aniconic’ (unornamented boulders); circles: ornamented and unornamented mortars. Figure prepared by D. Borić.

**Figure 3:**
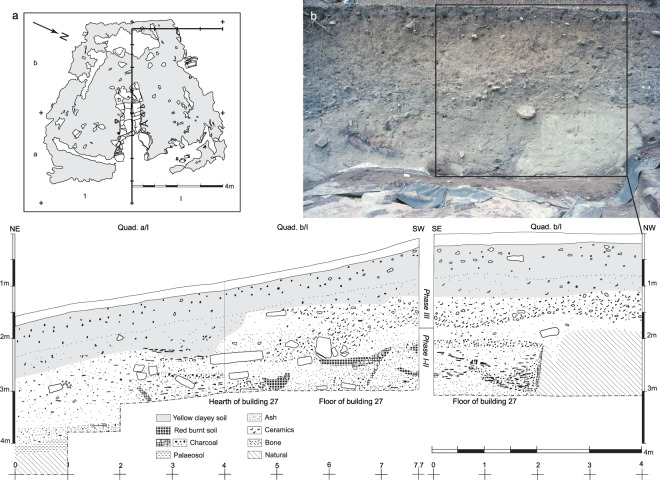
Representative stratigraphic sections exposed above the floor of building 27 at Lepenski Vir. (**a**) Section drawing 2 (1967): NE–SW along axis *y* through quad. a,b/I, 0–8 (7.7 m); section drawing 2’: SE–NW parallel with axis *x* through quad. b/I, 1–4 m. Figure prepared by D. Borić. (**b**) Photo by A. McPherron.

**Figure 4:**
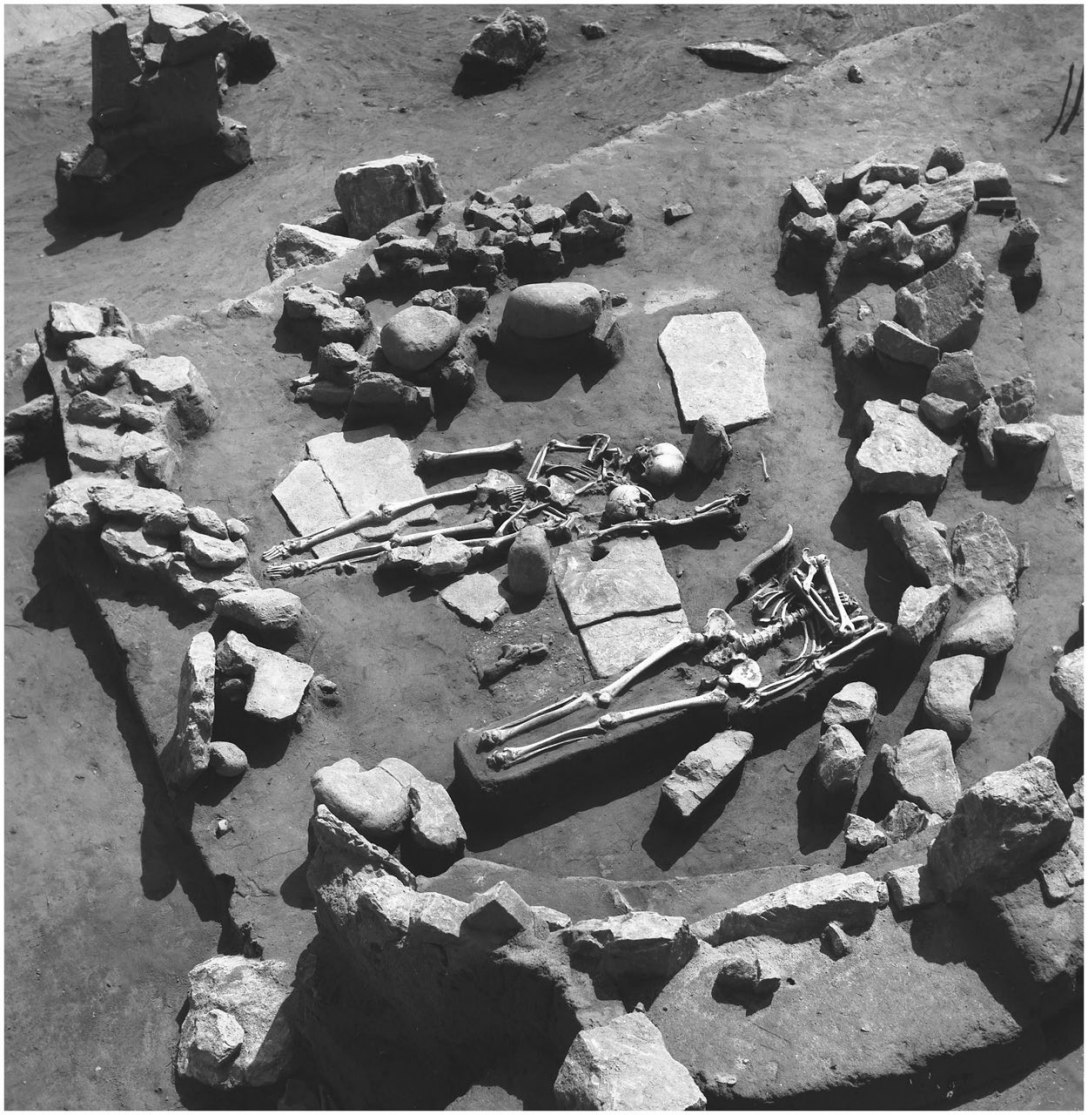
Building 65/XXXV from Lepenski Vir, phase I-II, and AMS-dated burials 54c, 54d and 54e placed over the floor. Photo courtesy of the Faculty of Philosophy, University of Belgrade.

**Figure 5:**
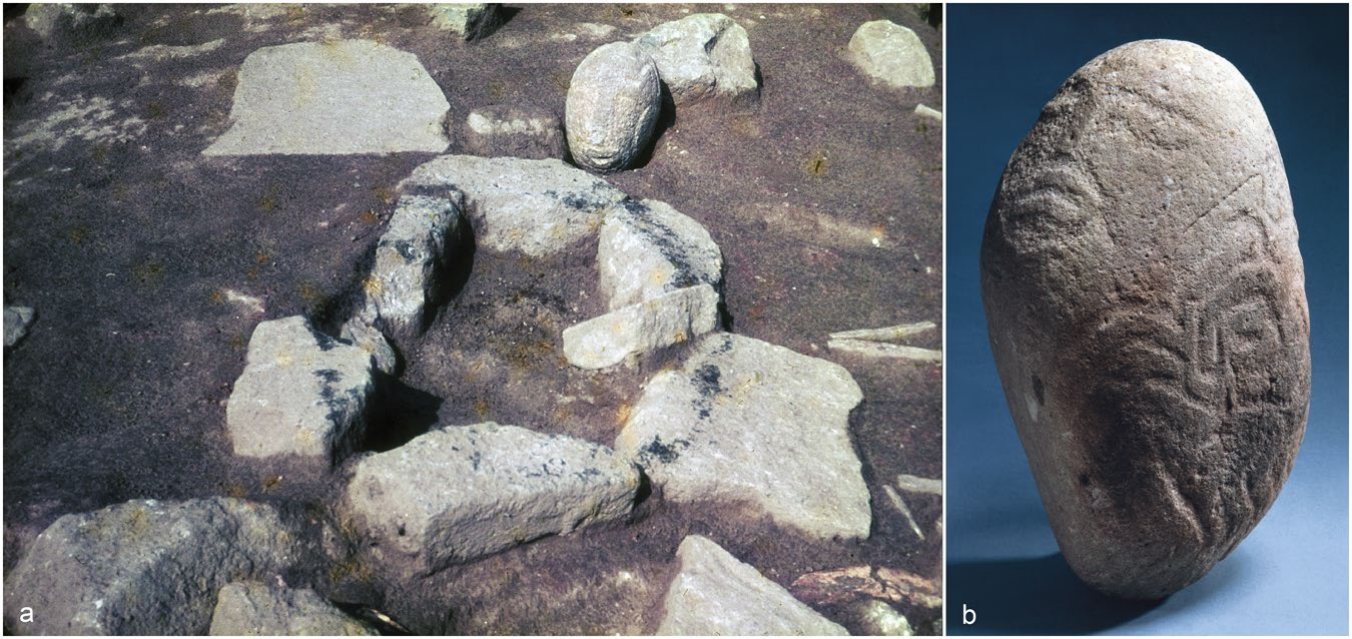
(**a**) Sculpted boulder *Shaman* found in the back part of the rectangular hearth of building 31 inserted in the limestone floor, Lepenski Vir phase I-II (18.5 × 16 × 22 cm; 8.5 kg). Photo by A. McPherron. (**b**) Sculpted boulder *Chronos* found next to a stone “path”/construction above the level of building 23, in front of building 32, facing down, Lepenski Vir phase I-II (36.5 × 23 cm; 21.86 kg). This figure is not covered by the CC BY licence. Credits to the National Museum in Belgrade. All rights reserved, used with permission.

Three aspects of Lepenski Vir evidence make the site better known than other contemporaneous sites in the Danube Gorges area. First, there are around 70 building structures that are elaborately furnished with reddish limestone floors and central rectangular stone-lined hearths (Fig. 2). These likely dwelling features, dug into the slope of the river terrace with the wider part facing the river, are semi-subterranean (Fig. 3) and absolutely unique in their architectural shape. The features were built across the river from the dominant landmark of the Treskavac Mountain that is also trapezoidally shaped. This position as well as the shape resemblance make it very likely that people consciously established relational links between the mountain and building floors as only here one finds a substantial concentration of trapezoidal structures with floors that were furnished with concrete-like limestone. Second, many of these dwelling features were transformed into tombs at the end of their life-use and were associated with human burials (Fig. 4), some accompanied by animal heads and disarticulated human remains, while there were also 40 neonate burials interred in 17 buildings^10^. Third, by and large, the trapezoidal building floor level or stone constructions around the floors were associated with artworks made of sandstone boulders of varying sizes (Fig. 5). The largest boulders weigh over 50 kg and were brought here from the upper reaches of the Boljetinska River, a tributary of the Danube, some 10 km away from Lepenski Vir^11^ (Supplementary Table S3). There were at least 94 objects that can be considered artworks, and these include sandstone boulders ornamented by geometric designs and, occasionally, depictions of human/fish hybrid faces or “X-ray” images of the fish body, mortars ornamented by geometric designs and the so-called “aniconic” boulders and mortars that were not engraved but were found in the same contexts as ornamented boulders, and hence can be considered to have had similar roles and were imbued with similar symbolic meanings. The spatial placement of boulder artworks beneath or on the level of building floors, often being built into the floor, all make it very likely that the context of production and use of these objects was contemporaneous with the occupation of trapezoidal buildings, i.e. that boulder artworks are exclusively associated with phase I-II (Fig. 2). This is further confirmed by the fact that boulder artworks commemorated some of the directly AMS-dated primary human burials: 7/I (building 21) and 61 (building 40) as well as currently undated primary burial 92 (building 28) interred into burial pits dug from the level of trapezoidal building floors either during buildings’ use as dwelling features or, alternatively, these burials marked events of buildings’ formal abandonment, also underlined by structured depositions of red deer skulls and antlers in buildings 21, 22, 26, 28, 57/XLIV, 45, 46 and 48^10^.

The dating and stratigraphy of Lepenski Vir has been surrounded by some controversy. According to the excavator, Srejović^1^, the archaeological deposits should be divided into four main phases. The earliest Mesolithic phase is labelled Proto-Lepenski Vir and is represented by rectangular stone-lined hearths without limestone floors and also includes a handful of burials. According to Srejović, this early development was followed by the main and best represented, also Mesolithic, phase I (with subphases a to f) with trapezoidally shaped limestone building floors, followed by final Mesolithic phase II, consisting of stone walls found at a higher level than trapezoidal building floors. Srejović suggested that the site was temporarily abandoned after phase II and again occupied by Neolithic groups that formed layers IIIa and IIIb on top of the Mesolithic settlement. This interpretation was at first challenged by a series of radiocarbon dates made on charcoal that indicated that phase I with trapezoidal buildings overlapped the duration of known Early Neolithic settlements in the adjacent regions^12–14^. Secondly, the neighbouring site of Padina, where buildings with the same trapezoidal outlines were also found albeit with a more modest settlement size, furnished evidence of Early Neolithic ceramics associated with trapezoidal building floors^15^. Srejović^16^ also reported ceramic finds in association with a number of trapezoidal buildings at Lepenski Vir, dismissing their association with the use of trapezoidal buildings and interpreting them as chronologically later intrusions. However, it has been shown that Early Neolithic Starčevo ceramics were unambiguously associated with the use of building floors and backfill of trapezoidal buildings^17–20^. In the 2000s, it has been suggested that Srejović’s phases I and II are one and the same phase, where the stone walls previously assigned to phase II were in reality retaining walls for dugout trapezoidal building floors of phase I features^18,21^ (Figs 2 and 4). However, most recently, Perić and Nikolić^22^ suggested that trapezoidal buildings with boulders should not be linked with the material culture or burials found on building floors, which they exclusively associate with Neolithic intrusions. In their view, the construction of trapezoidal buildings predates 7,500 cal BC and no material culture was associated with these features.

This persistent stratigraphic and dating controversy has limited the impact of important evidence from Lepenski Vir in discussions about forager-farmer/Mesolithic-Neolithic transitions in Europe. With the availability of modern and improved dating techniques, in particular Accelerator Mass Spectrometry (AMS), enabling dating of short-lived single entity materials, it became possible to repay a visit to this sequence by providing new dates on human remains and a reanalyzed collection of animal bones as well as some of the preserved charcoal samples. Over the past 15 years, a substantial number of new dates have accumulated making it possible for the first time to provide formally modelled estimates for the duration of particular phases at the site within the Bayesian statistical framework. In addition, the available excavation archive makes it possible to provide a detailed reworking of the site’s stratigraphy^10^ (see Supplementary Information).

## Material and Methods

There are 111 radiocarbon measurements from Lepenski Vir (Supplementary Table S1): 25 charcoal dates including two sets of duplicate measurements and one set of triplicate measurements; 47 AMS dates on animal bones that include two sets of duplicate measurements; and 39 AMS dates on human bones, including four sets of duplicate and one set of triplicate measurements on the same skeletons. One set of duplicate measurements from the same burial come from the same lab while the other sets of duplicate measurements on the same burials were obtained in two different laboratories. Ninety measurements were previously published^21,23–27^, while 21 measurements are published here for the first time. Mesolithic and Neolithic contexts discussed herein are dated with 105 charcoal, human and animal bone dates, while 6 AMS measurements date Copper Age, Roman and Medieval burials.

Dates were produced by nine radiocarbon laboratories, using a variety of methods (Supplementary Table S2). Not all of these measurements are conventional radiocarbon ages^28^. From an archive of charcoal samples from this site we obtained new dates on four samples of different wood species (*Pinus sylvestris* type, *Quercus* sp. deciduous and *Cornus* sp.) achieving higher precision results than charcoal dates made in the late 1960s.

Most of the AMS-dated samples on animal bones were processed at the Oxford Radiocarbon Accelerator Unit (ORAU) in 2005 and 2011–2016 using collagen extraction^29^, followed by the revised gelatinization and filtration protocol described by Bronk Ramsey *et al*.^30^, and dated by AMS as outlined in Bronk Ramsey^31^. First five OxA- AMS dates on human burials were obtained in 1996^23^. AMS dates on human bones from 20 Lepenski Vir burials, among which 14 date Mesolithic-Neolithic contexts, were obtained between 2000 and 2002^24^ and are affected by a technical problem in the ORAU, which used the ultrafiltration protocol in this period, obtaining measurements that could be around 100–300 radiocarbon years too old. That measurements on ultrafiltrated human bone samples from Lepenski Vir provide dates that are too old was confirmed by duplicate results obtained on four burials (burials 7/I, 8, 26 and 54d) measured along with three other human bone samples from Lepenski Vir in the NSF Arizona AMS Facility^25^. The burials with ultrafiltrated OxA- dates have recently been re-dated and replaced by a new set of AMS dates^32^, which have been used in our Bayesian modelling. There are three additional AMS-dated samples on human bone that were processed at the Laboratory of Quaternary Chronology, University of Beijing (Supplementary Table S2).

Stable isotope measurements produced on human bone as part of the AMS or subsequent measurement process indicate that individuals represented here subsisted off a diet that had a varying fish contribution. It has long been known that diet-derived reservoir offsets effect radiocarbon dates on Mesolithic and Neolithic human skeletal remains from the Danube Gorges region^33,34^. To produce accurate date ranges for the date of death of individuals it is necessary to correct measurements for the offset. The most sophisticated methods of investigating offsets analyze measurements of multiple stable isotopes — sulphur, carbon and nitrogen^35^. While not all sulphur values from the bone collagen used for the Lepenski Vir human radiocarbon measurements are currently available, in Supplementary Table S1 we provide sulphur values for a number of Lepenski Vir burials. We have calculated diet-derived freshwater fish offsets from the δ^15^N and δ^13^C values using two methods outlined by Cook *et al*.^33^. We appreciate that the estimates for the percentage protein/fish component represent relatively crude reflections of the *in vivo* signal, both because of concerns with the reproducibility of the δ^15^N and δ^13^C signals and the mixing models applied (Supplementary text 1). One AMS-dated sample on a bone tool (OxA-26554: SI Table 1) was analyzed with collagen peptide mass fingerprinting, also known as Zooarchaeology by Mass Spectrometry (ZooMS)^36^ due to unusual stable isotope values (Supplementary text 2).

Calibrated results presented here have been calculated using the curve of Reimer *et al*.^37^ and the computer program OxCal (v. 4.3.2)^38–40^. The ranges cited in the text are quoted in the form initially recommended by Stuiver and Polach^28^ but adapted for the increased precision available in later datasets, with the end points rounded outwards to 10 years as the error terms are greater than 25 radiocarbon years. The ranges in plain type have been calculated according to the maximum intercept method^41^. The ranges quoted in italics are *posterior density estimates* derived from the Bayesian modelling outlined below (Supplementary text 3). The calibrated probability distributions shown in the figures have been calculated using the probability method^42^. Groups of dates representing multiple observations on a single entity were combined.

**Figure 6:**
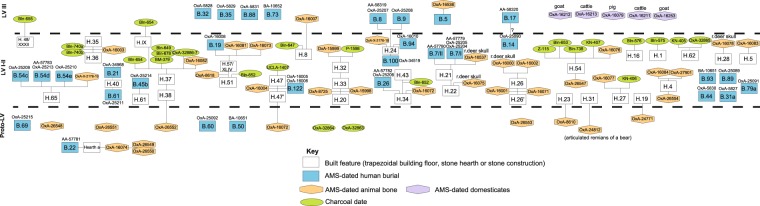
A Harris matrix showing stratigraphic relations between radiocarbon dated contexts and samples within the proposed stratigraphic model for Lepenski Vir. Figure prepared by D. Borić.

Our chronometric work identifies three main phases (Fig. 6). The Early-Middle Mesolithic phase is equated in the original stratigraphy with the label Proto-Lepenski Vir. This phase is primarily linked to contexts beneath trapezoidal building floors or those found around rectangular “open-air” hearths, which in construction and proportions differ from those found in trapezoidal buildings and have no limestone floors associated with them. The phase is also linked to several Mesolithic burials, including one seated burial with crossed lower limbs (Supplementary Fig. S2). The phase is followed by the Mesolithic-Neolithic transition phase, which is equated with combined phases I and II of the original stratigraphy of the site, i.e. mainly contexts associated with trapezoidal buildings and Mesolithic extended supine burials with largely uniform orientations predominantly found in buildings but occasionally also in the space outside buildings (Supplementary Fig. S3). Finally, the Early Neolithic phase is equated with the original definition of phase III, and marks post-trapezoidal building phase features, such as pits and domed ovens, the appearance of first domesticates and burials in crouched positions (Supplementary Fig. S4). The boundaries of these phases are allowed to overlap – the data is allowed to decide the relationships among them. Stratigraphic relationships identified by previous workers^1,7,10,21,43^ were incorporated into the model.

**Figure 7:**
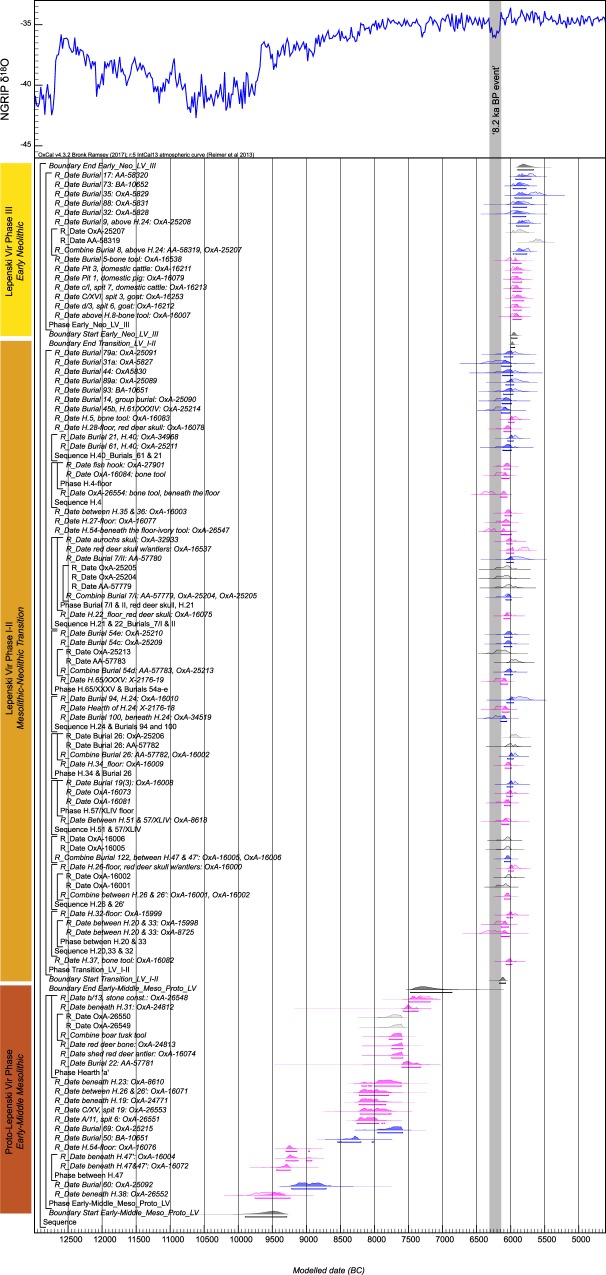
Preferred Bayesian Model 2 with posterior distributions of radiocarbon measurements from Lepenski Vir (*n* = 80) plotted against the North Greenland (NGRIP) δ^18^O_ice_ record and event stratigraphy. For the radiocarbon measurements, distributions in outline are the results of simple radiocarbon calibrations, solid distributions are the output from the chronological model (for the CQL code see Supplementary text 5). Blue: human bone; magenta: animal bone.

In order to obtain robust and reliable results not dependent on the type of samples dated, the same stratigraphic interpretation of the site was modelled by including (a) all available radiocarbon dates (we term this Model 1, Supplementary Fig. S5A), (b) all dates on short-lived single entity materials only thus excluding charcoal dates (Model 2, Fig. 7) and (c) only AMS dates taken from entities that were found as articulations (e.g. primary burials) under the assumption that these do not represent residual and redeposited remains but freshly deposited carcasses (Model 3, Supplementary Fig. S5B). We used Outlier detection methods in our models^44^ to detect results that are outlying with respect to others in the model. These are down-weighted in different iterations of the models. The results tend to be an average of a model in which the measurement is accepted as reliable and one in which it is removed. A sample with an outlier posterior probability of 50% means that it is ignored in the model half the time the model is run.

## Results

Comparison of the results allows us to gauge the impact of potentially problematic charcoal samples or dated residual non-articulated bone material on our conclusions (Table 1). Samples which are clear outliers (~90–100% outlying) are few (there are 6 in Model 1, listed in Supplementary Table S1) and the majority of likelihoods are less than or equal to the prior outlier values (5%) given. There is a similarity in the start, end and duration of identified phases regardless of the inclusion or exclusion of possibly problematic sets of dates, which is encouraging and allows us to draw robust conclusions about the duration of particular phases. However, our preferred model is the one that relies on human and animal bones only (Model 2), in this way removing the possible bias of problematic older charcoal dates (Fig. 7).

**Table 1:** Comparison between posterior density estimates of the start, end and duration of particular phases at Lepenski Vir based on different sets of dates being modelled at 95% probability (unless otherwise stated) and expressed as cal BC.

Stratigraphic phase	Model 1: All dates (human, animal bone and charcoal) (*n* = 105)	Model 2: All animal and human bone (*n* = 80)	Model 3: Articulates only (burials and animal articulates) (*n* = 35)
**Early Neolithic LV III**			
End	*5,87*0–*5,490*	*5,900*–*5,660*	*5,850*–*5,610*
Start	*5,970*–*5,910*	*5,990*–*5,900*	*5,960*–*5,760*
Duration	*60*–*470 years*	*10*–*300 years*	*0*–*280 years*
**Mesolithic-Neolithic Transition LV I-II**			
End	*5,980*–*5,930*	*6,010*–*5,940*	*6,020*–*5,860*
Start	*6,160*–*6,080*	*6,170*–*6,070*	*6,150*–*6,020*
Duration	*120*–*210 years*	*80*–*210 years*	*20*–*260 years*
**Epipalaeolithic?/Early-Middle Mesolithic Proto-LV**			
End	*7,490*–*6,840*	*7,480*–*6,860*	*7,540*–*6,240*
Start	*10,360*–*9,350 (93.8%) 12,550*–*12,300 (1.6%)*	*9,900*–*9,280*	*10,530*–*8,620*
Duration	*1,990*–*3,400 years*	*1,930*–*2,870 years*	*1,220*–*3,630 years*

The site was likely occupied from the beginning of the Holocene in *9,900–9,280 cal BC (95% probability; Start Early-Middle_Meso_Proto_LV*) probably in *9,660–9,370 cal BC* (*68% probability)*. The Early and Middle Mesolithic phases (Proto-Lepenski Vir) might have been discontinuous. However, samples dating features of both phases are limited and do not come from stratigraphically superimposed contexts and at present only indicate a long spread of the posterior density distributions. There is a density of dates possibly relating to the Middle Mesolithic phase over the 8th millennium cal BC. The estimate for the end of this early period in *7,480–6,860 cal BC* (*95% probability; End Early-Middle_Meso_Proto_LV*), probably in *7,420–7,140* *cal BC* (*68% probability*), corresponds with the date for the end of the Middle and the start of the Late Mesolithic at other sites in the Danube Gorges area^21,45^. The Proto-Lepenski Vir phase almost certainly did not overlap the Mesolithic-Neolithic Transition phase with trapezoidal buildings and boulders (phase I-II). There appears to have been a hiatus of at least 700 years between these phases. Surprisingly, the Transition phase I-II, most prominently represented at the site with the build-up of trapezoidal buildings and proliferation of ornamented boulder artworks, was short, starting in *6,170–6,070* *cal BC (95% probability; Start Transition_LV_I-II*), probably in *6,140–6,090* *cal BC* (*68% probability*), and ending in *6,010–5,940* *cal BC (95% probability; End Transition_LV_I-II*), probably in *5,990–5,960* *cal BC* (*68% probability*). The duration of the phase is estimated between *80 and 210 years (95% probability; Duration Transition_LV_I-II*), probably between *110* *and 170 years* (*68% probability*), i.e. anywhere between three and eight generations taking 25 years as a generation or two to four lifespans, taking 50 years as a lifespan. Early Neolithic phase III almost certainly overlaps with the later part of phase I-II. Phase III started in *5,990–5,900* *cal BC (95% probability; Start Early_Neo_LV_III*), probably in *5,980–5,930* *cal BC* (*68% probability*) and ends in *5,900–5,6*60 *cal BC (95% probability; End Early_Neo_LV_III*), probably in *5,870–5,740* *cal BC* (*68% probability*).

## Discussion

Charcoal dates with the laboratory codes “Bln-” and “Z-” ought to be treated with caution. We found in Model 1 that of the 14 determinations 8 yielded partial (>10%) or significant (75%+) outliers, often appearing later than results with which they may be associated, despite sometimes large standard errors (though issues of the taphonomy of dated samples make this difficult to ascertain with any degree of certainty). These determinations are downweighted in Model 1 as described above. New charcoal dates associated with trapezoidal buildings (duplicates OxA-32886-7 dating *Quercus* sp. charcoal from building 37 and OxA-32865 dating *Cornus* sp. charcoal from building 62), obtained in the Oxford lab on archive samples for the purpose of the current project, are more precise than the 1960s and 1970s measurements and also fit better with the proposed stratigraphic model.

OxA-26547, which dates a wild boar tusk tool found beneath the floor of building 54 (Fig. 8.15) and OxA-26554 dating a possible tapered base of a bone point found beneath the floor of building 4 (Fig. 8.12) yielded higher than usual posterior outlier probabilities (10% and 70% outlying). OxA-26554 also had unusual stable isotope values (δ^13^C = 18.0‰, δ^15^N = 12.0‰, Supplementary Table S1); ZooMS analysis identified the specimen as deriving from canine bone (Supplementary Fig. S1), which explains its stable isotope values as the sample was affected by the freshwater reservoir effect due to the dog’s consumption of fish. This is currently the only confirmed example of a canine bone being used for bone tool making in this regional context. To this measurement, we applied the same correction factor applied to human bones affected by the reservoir effect. However, there remains a question of applicability of this correction in the case of dog bones and this may add further uncertainty to the reliability of the measurement, possibly influencing its higher posterior outlier probability. If taken as correct, there could be two different explanations for higher than usual posterior outlier probabilities of these two measurements. First, at the time of their use, these tools might have been curated and a century or so old, being brought to the site at the start of the buildings’ construction, c. 6170 cal BC or later, and might have been discarded before the floors of these buildings were furnished. Alternatively, measurements on these tools might be indications of a currently unrecognized and undated phase of occupation at the site before the onset of the construction activities associated with phase I-II, sometime between c. 6400 and 6200 cal BC. This possible occupation phase may or may not have been continuous with phase I-II. At the moment, it is not possible to make a strong case for either of these two scenarios and further dates from similar contexts beneath building floors might resolve this issue in the future. Yet, both dates could be taken as reliable and acceptable *termini post quos* for the start of building activities at the site.

**Figure 8:**
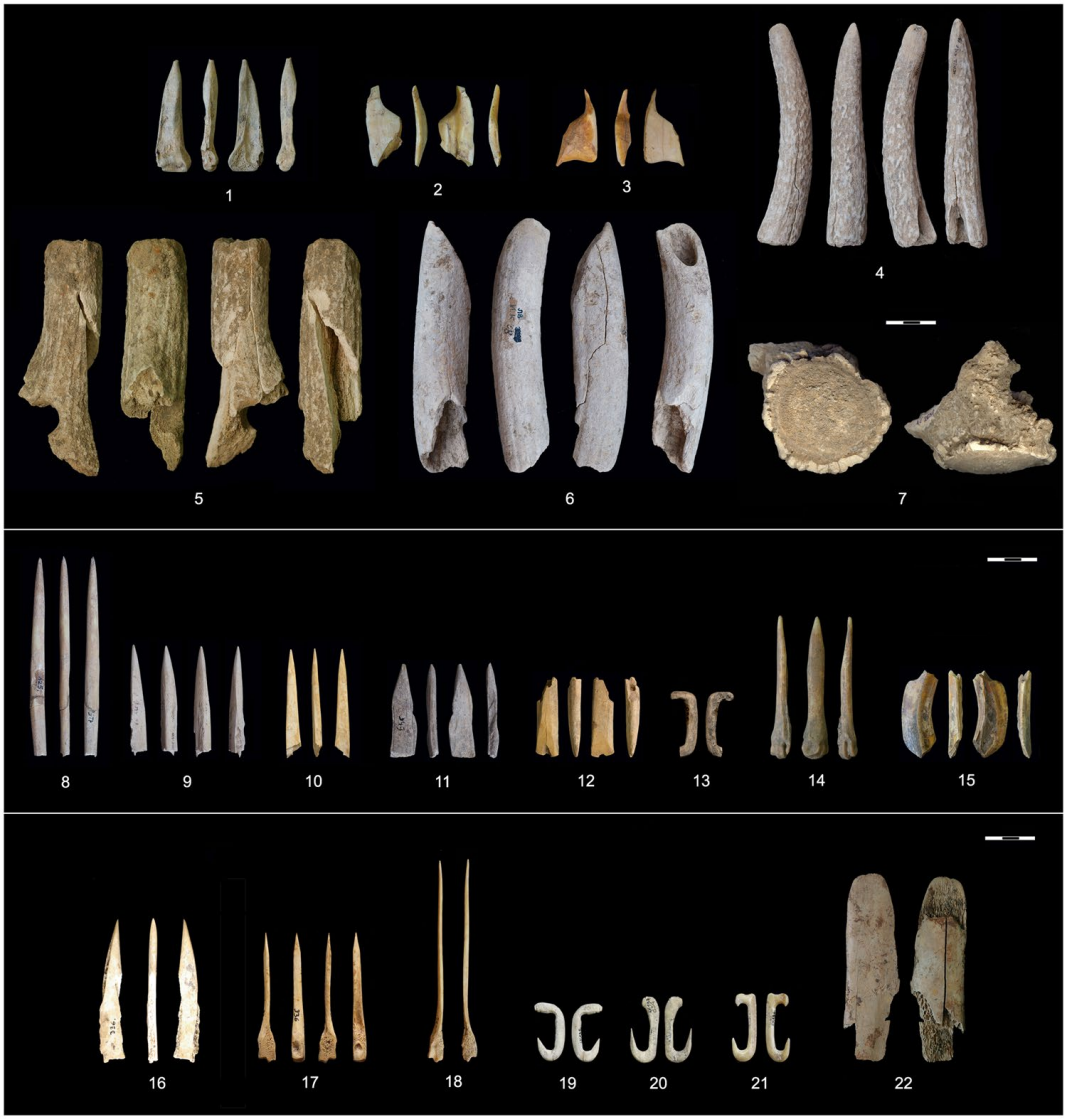
AMS-dated osseous tools and several representative examples of undated osseous tool morphologies from Lepenski Vir by the main stratigraphic phases. *Early/Middle Mesolithic (Proto-Lepenski Vir):* 1. OxA-26553, quad. C/XV, spit 19; 2 (inv. BB-106). OxA-26548, quad. b/13, stone construction (inv. BB-112); 3. OxA-26549, -26550, hearth “a” (inv. BB-187); 4. OxA-24771, beneath the floor of building 19; 5. OxA-26551, quad. A/11, spit 6 (inv. BB-23); 6. OxA-26552, beneath the floor of building 37; 7. OxA-16074, hearth “a”; *Mesolithic-Neolithic Transition Phase (Lepenski Vir I-II)*: 8. OxA-16083, building 5, floor level (inv. 125); 9. OxA-16081, building 57/XLIV, stone construction (inv. 689); 10. OxA-16082, House 37, floor level (inv. 673); 11. OxA-16084, building 4, floor level (inv. 349); 12. OxA-26554, beneath the floor of building 4 (inv. BB-193); 13. OxA-27901, building 4, floor level (inv. 350); 14. not dated, building 62 (inv. BB-79); 15. OxA-26547, beneath the floor of building 54 (BB-209); *Early Neolithic (Lepenski Vir III)*: 16. OxA-16007, stone construction above building 8, spit 7 (inv. 336-1); 17. not dated, stone construction above building 8, spit 7 (inv. 336-2); 18. not dated, quad. b/I, spit 5 (inv. 563); 19. not dated, quad. d/4, spit 8 (inv. 857); 20. not dated, quad. c/II, spit 6 (inv. 854); 21. not dated, no context (inv. 906); 22. not dated, quad. c/II, spit 2. Photos by D. Borić and E. Cristiani.

A particularly important *terminus post quem* for the construction of trapezoidal buildings and limestone floors are articulated remains of burial 100 found beneath the floor of building 24 dated by OxA-34519. Other *termini post quos* for dating the construction of these architectural features are OxA-16003 (dating animal remains between the overlapped floors of buildings 35 and 36), OxA-8618 (dating animal remains between the overlapped floors of buildings 51 and 57/XLIV), duplicate measurements OxA-16005 and OxA-16006 (dating disarticulated child skull burial 122 between the overlapped floors of buildings 47 and 47′), duplicate measurements OxA-16001 and OxA-16002 (dating animal remains between the overlapped floors of buildings 26 and 26′), OxA-8725 and OxA-15998 (dating animal remains between overlapped floors of buildings 20 and 33). The construction/use of building 40 is dated by OxA-34968 that directly dates disarticulated mandible burial 21. This mandible was inserted into the floor of this building, forming, along with a stone plaque, a ∀-shaped support typically found associated with hearths of various other trapezoidal buildings at the site (e.g. Figs 3a and 5b), as a unique constructional tradition found only in this region during this period. There are also significantly older results dating the remains of the Proto-Lepenski Vir phase occupation that also act as *termini post quos* for architectural units with trapezoidally shaped floors: OxA-26552, OxA-16004, OxA-24771, OxA-8610 and OxA-28412. All of these determinations fit well within the models.

Three dates on samples that come from the floors of trapezoidal buildings are interpreted as residual materials in these contexts as remains of older Early-Middle Mesolithic occupation (Supplementary Table S1). OxA-16004 dates a sample of Early Mesolithic age from the context between the superimposed floors of older building 47′ and younger building 47; however, this context is dated with a set of duplicate measurements OxA-16005 and OxA-16006 that date disarticulated skull burial 122 and confirm the expected date for phase I-II. It is similar in the case of OxA-16071 that dates a sample of Early Mesolithic age from the floor of building 26′ superimposed by the floor of building 26; this context is also dated by a set of duplicate measurements OxA-16001 and OxA-16002 that correspond well with the estimates for the duration of phase I-II. Finally, OxA-16076 dates another residual (Early Mesolithic) red deer antler sample from the floor of building 54, while OxA-26547 on a wild boar tusk tool found beneath the floor of the same building (despite a higher outlier probability of 35% in Model 2, see above), provides an acceptable, much later *terminus post quem* date for the start of building activities at the site that is in line with the estimates for the start of phase I-II. The most parsimonious explanation for the presence of residual material, especially in the two instances when these are found between superimposed building floors, is the fact that turned-over deposits, which contained residual materials from earlier occupation of the site, were used for levelling the ground over older trapezoidal floors before a new building was constructed.

Five measurements obtained on animal bone samples dating domesticates (goat, cattle, and pig) from phase III features suggest that these were introduced to the site by *6,000–5,900* *cal BC (95% probability)*. No domesticates were associated with the occupation of phase I-II trapezoidal building floors^7^ which suggests strongly that domesticates from several pit features date to the later Early Neolithic phase III.

With 15 samples we have also dated a range of antler, bone and wild boar tusk tools from different phases of occupation at Lepenski Vir (Fig. 8). These measurements directly date diagnostic elements of osseous industry and can be used as an additional check of diachronic changes in the operational sequence of artefact production between the Mesolithic and Neolithic occupation phases. Most of the osseous tools associated with contexts underneath building floors and those found on building floors exhibit common elements of a sequence of production gestures in the process of making tools, i.e. a typical Mesolithic *chaîne opératoire*, dominated by red deer antler intermediate pieces and mattocks used for heavy-duty activities, pointed tools on cervid proximal metapodials, curated points, sometimes with a tapered base, and combined pointed and cutting-edged wild boar tusk tools. This suggests a long continuity of the local tradition of osseous material working from the Early Mesolithic through to the Mesolithic-Neolithic Transition phase. However, in association with transitional phase contexts, for the first time appear new tool types that can be linked to a new technological *chaîne opératoire*, typical of Early Neolithic groups’ traditions of working osseous materials. OxA-27901 from the floor of building 4 dates a fishhook between *6*,*100*–*6,000* *cal BC (95% probability)* (Fig. 8.13). This fish hook was made with a newly introduced mechanical drilling technique (bow or pump drill) that might have arrived in the Danube Gorges area with migrant women who appeared at Lepenski Vir during the transition phase based on strontium isotope^25^ and aDNA evidence^46^, and who might have transmitted to the indigenous population this new technique, a range of new osseous tool types (e.g. flat symmetrical awls with half-worked distal metapodial epiphysis manufactured from longitudinal halves [Fig. 8.14, 17, 18]) as well as new types and materials for body adornment^45^. These new tool types became dominant in the repertoire of osseous tool making during Early Neolithic phase III.

Direct dates on human bone from five primary inhumations (burials 8, 17, 32, 73 and 88) placed in crouched positions suggest that these were restricted to phase III in *5,980–5,640* *cal BC (95% probability)*. This dating corresponds with the expectation that the crouched-flexed burial position reflects a typical Neolithic burial rite that was introduced to the site during phase III for the first time^10^.

We have compared calibrated and modelled radiocarbon ages from Lepenski Vir to North Greenland (NGRIP) δ^18^O_ice_ record and event stratigraphy (Fig. 7) in order to examine possible correlation between recorded climatic changes and the distribution of posterior density estimates throughout the occupation of Lepenski Vir. In particular, we looked at the possible correlation between the start and duration of Mesolithic-Neolithic transition phase I-II and the timing of the so-called 8.2 ka cal BP cooling event. Bonsall *et al*.^47,48^ have previously argued that this particular climatic oscillation dated between c. 6300 and 6100 cal BC might have affected significantly Late Mesolithic settlement pattern in the Danube Gorges area along the river edges and especially low-lying portions of the sites due to the increased risk of flooding, thus prompting relocation of settlements. It should be noted that to date the only evidence used to argue this scenario in the Danube Gorges area comes from the distribution of radiocarbon dates with arguable “gap” in the occupation of all sites except Lepenski Vir during this cold spell. Yet, this argued “gap” has been contested based on the radiocarbon evidence from several other sites in the region^19,49^. Bonsall *et al*.^47^ also suggested that limestone floors of Lepenski Vir might have been built in response to flooding while sculpted boulder fish-human hybrid depictions might have been carved in order to appease “river gods”. These authors argued that the site was not relocated like other contemporaneous settlements due to its “sacred” character and importance.

Our high-resolution Bayesian modelling of the large series of radiocarbon dates from Lepenski Vir, and in particular those associated with phase I-II, allows us to re-examine with high accuracy a correlation between this rapid climate change and resettling of Lepenski Vir during the Mesolithic-Neolithic transition phase I-II. The timing of the start of this phase in *6,170–6,070* *cal BC (95% probability; Start Transition_LV_I-II*), probably in *6,140–6,090* *cal BC* (*68% probability*) suggests that after a gap in the occupation of the site during the Late Mesolithic, when several other neighboring sites and those farther afield saw the most intense periods of occupation, the phase associated with the construction of trapezoidal buildings most likely started after or at the very end of the duration of the 8.2 ka cal BP event. This evidence of correlation should not necessarily be taken as evidence of causation even though the close timing of these two phenomena requires further research and attention. At this stage, it can be suggested that even though it is not likely that Lepenski Vir was abandoned due to this particular climatic oscillation, its reoccupation with elaborate building construction and even the proliferation of boulder artworks could in some ways be connected with or be a reaction to what might have been observed as a dramatic change in climatic conditions and the productivity of the river Danube. Yet, if Lepenski Vir is to be considered special and “sacred” when compared to other contemporaneous sites in the region based on the evidence associated with phase I-II, this happened after not before 8.2 ka cal BP event.

Bonsall *et al*.^48^ also suggested that lower lying parts of Lepenski Vir were affected by flooding, which might have removed datable material during floods, prompting people to move their dwelling structures to higher parts of the site. This scenario is unlikely on the basis of the evidence presented here for two reasons. First, we have presented dates on datable material from buildings found in the lowermost part of the site (e.g. building 4 with three new AMS dates, Fig. 2, Supplementary Table S1), and second, these dates are neither significantly older nor younger than the dates for trapezoidal structures farther up the slope. There is a theoretical possibility that the site was occupied during the Late Mesolithic prior to 8.2 ka cal BP event and that flooding removed the remains of this occupation phase, rendering it “invisible” in the archaeological record and hence undatable. However, in this case we would expect to find the remains of such occupation farther up the slope at this site as it is unlikely that only lower portions of the site would have been occupied. For instance, Early/Middle Mesolithic dates come from different locations on the topographic gradient of the site, and it would be unusual that the Late Mesolithic occupation was restricted to the low-lying parts of the site only, and it is also unlikely that the flooding removed absolutely all Late Mesolithic deposits across the spread of the site as Late Mesolithic occupation deposits were found at other settlement locations in the region at similar altitudes. Hence, we suggest that the current gap in the occupation of Lepenski Vir during the Late Mesolithic is real and not the effect of erosional events and that our estimates for the start of phase I-II are not affected by a preservation bias caused by flooding. Finally, in order to argue with some confidence about the region-wide pattern of settlement relocation at other sites based on the distribution of radiocarbon dates, similar chronological datasets to the one now available for Lepenski Vir should be available for the application of high-resolution Bayesian modelling. At the downstream site of Schela Cladovei, robust dating evidence does seem to indicate a gap in the occupation of this site during the 8.2 ka cal BP event.

The reoccupation of Lepenski Vir with the start of phase I-II is characterized by evidence of heightened levels of symbolic expression and concentration of special purpose objects, such as sculpted boulder artworks, all of which were seen in this region neither at chronologically earlier nor contemporaneous sites. There is now also strong evidence for the presence of Neolithic northwest Anatolian ancestry among individuals buried here during this phase. All this suggests that at this pivotal historical moment Lepenski Vir became an important hub of cultural exchanges and mixing between indigenous foragers and the first farming groups. Evidence that some of the individuals were buried inside trapezoidal buildings following typical Mesolithic rites of extended supine inhumations despite of their “pure” Anatolian Neolithic ancestry (AMS-dated burials 61 and 54e^46^, Fig. 4) may suggest that during this phase the power relationship was in favour of the indigenous forager community. This was possibly due to the fact that farming groups were still few and far between. We may only speculate that the timing of the coming together of these two genetically and culturally different populations at this newly reestablished locale just after a period of rapid climatic deterioration was not coincidental, and that the settlement and its elaboration were meant to celebrate this new reality and a productive merging of the two cultural worlds. Hence, rather than envisaging mechanistic one-size-fits-all responses to climate oscillations in prehistoric past, we may suggest a more nuanced way to incorporate complex strands of evidence from climatic proxies, genetic evidence of mixing of different populations and details of archaeological context. Crucial for a successful coming together of these different strands of data is the ability to provide a high-resolution chronological timeline.

## Conclusions

Our formal chronological modelling of the robust series of dates from Lepenski Vir has firmly set the date for the context of architecture and boulder artworks and has important implications for understanding mechanisms involved in the transition to farming in this key region of southeast Europe. Phase I-II offers a rare opportunity to observe the pace of and the impact that the spread of Neolithic groups had on the local forager population and their cultural expression. There is no other region in southeast Europe that preserves such richness of archaeological evidence for the Mesolithic-Neolithic transitional phase. Extensive absolute dating of various features at the site also allowed us to revise the chronological and stratigraphic understanding suggested by its excavator.

While we cannot exclude the possibility that the site was occupied during the Late Mesolithic, this is very unlikely. We have shown that after several centuries of an occupational hiatus after the Early and Middle Mesolithic use of the site, the construction of trapezoidal structures started at the time when Early Neolithic groups were already established in the adjacent regions of the Balkans^27^. We argue that this timing strongly suggests that it was precisely contacts and exchanges, which involved both women and material goods^25^, between Early Neolithic farming groups and the Danube Gorges foragers that triggered the burst of cultural and symbolic creativity found at Lepenski Vir. This was expressed in the elaboration of an older, autochthonous dwelling blueprint and in an unprecedented rate of construction of trapezoidal buildings with hard reddish limestone floors over only a couple of generations at the location directly across a mountain that was possibly considered sacred. These structures were accompanied by a similarly high rate in the proliferation of sandstone boulder artworks (Supplementary Table S3), the style of which never occurred in earlier Mesolithic phases of occupation in this region. While we cannot claim that similar decorative motives and depictions did not exist earlier in some other, perishable media (e.g. wood, body painting), it is for the first time during phase I-II that such imagery occurs in durable, non-perishable material form. The style of these artworks is absolutely unique to this region and likely represents an objectification of indigenous forager mythology, symbolism and beliefs^10,11^.

We have noted that the timing of the reoccupation of Lepenski Vir and start of phase I-II based on our current posterior density estimates when modelling radiocarbon dates within the Bayesian statistical framework corresponds with the end of 8.2 ka cal BP event of rapid climate deterioration. While we should refrain from immediately inferring a causal relationship between the two, this correlation could be significant for the cultural history of Lepenski Vir and should be explored further, especially in the context of similar high-resolution sets of dating evidence to be obtained from other sites in the region in the near future.

The observed and now dated changes in the technological production sequence with the introduction of novel, Neolithic materials and techniques during phase I-II, may suggest that new material culture practices and aesthetics of living and crafting were introduced during phase I-II by immigrant women^10,25,46^ and transmitted through processes of vertical and horizontal learning. After only two to four lifespans from the first contacts and interactions with Early Neolithic farming groups and a short flourishing of this indigenous forager stronghold in the region, the local cultural specificity was entirely subsumed by novel, Neolithic subsistence, material practices and beliefs. The establishment of a high-resolution chronological framework for these key developments at Lepenski Vir allows this site to take its rightful place in European and World Prehistory.

## Electronic supplementary material

Supplementary Information
