# Supplementary material for: High-Resolution AMS Dating of Architecture, Boulder Artworks and the Transition to Farming at Lepenski Vir
Source: Sci Rep. 2018 Sep 21;8:14221. doi: 10.1038/s41598-018-31884-7 (PMC6155048; doi:10.1038/s41598-018-31884-7)
Supplement: Supplementary file 1 — Supplementary Information [file 41598_2018_31884_MOESM1_ESM.docx]

**High-Resolution AMS Dating of Architecture, Boulder Artworks and the Transition to Farming at Lepenski Vir**

Dušan Borić, Thomas Higham, Emanuela Cristiani, Vesna Dimitrijević, Olaf Nehlich, Seren Griffiths, Craig Alexander, Bojana Mihailović, Dragana Filipović, Ethel Allué & Michael Buckley

**Supplementary Information**

**Supplementary Table S1**. Radiocarbon measurements on charcoal, animal and human bones from Lepenski Vir. Ages are corrected for those dates that have δ^15^N values >+9.5‰ (affected by the aquatic reservoir effect) as suggested by Cook *et al.*^1-2^. The δ^15^N values used to estimate percentage of aquatic diet. Method 1: A weighted mean age offset for a 100% fish-based diet estimated as 540±70 radiocarbon years. Method 2: δ^15^N values >13.0 = 100% reservoir correction applied (440±45 years); δ^15^N values <13.0 = 50% reservoir correction applied (220±23 years). Dates are calibrated with OxCal v. 4.3.2^3-4^. Orange shading: animal bone samples; blue shading: human bone samples; green shading: charcoal samples; white: unknown whether human or animal.

| **Laboratory code** | **Sample material and reference** | **Sample association** | **Radiocarbon age (BP)** | **δ^13^C**  **(‰)** | **δ^15^N**  **(‰)** | **δ^34^S**  **(‰)** | **C:N** | **% protein (fish?) signal** | **Radiocarbon measurement corrected for average freshwater fish offset (Cook *et al.* 2011) BP** | **Calibrated date (95% confidence)**  **cal BC or cal AD** | ***Posterior density estimate for Model 1 (95% probability unless otherwise stated) cal BC*** | **Source** |  |
| --- | --- | --- | --- | --- | --- | --- | --- | --- | --- | --- | --- | --- | --- |
| **Proto-Lepenski Vir Contexts (Early-Middle Mesolithic phases)** | | | | | | | | | | | | | |
| OxA-32864 | *Pinus sylvestris* (S. no. 7) | Quad. c/I-II (hearth) (22/08/1968) | 12,335±50 | –24.2 | – | – | – | – | – | 12,730–12,140 | *12,370–12,170 (2.6%)*  or  *10,060–7430 (92.8%)*  *(poor agreement 10%)* | This paper |  |
| OxA-32863 | *Pinus sylvestris* (S. no. 6) | Quad. A/3, LV horizon II, beneath rock (18/10/1967) | 10,075±45 | –24.3 | – | – | – | – | – | 10,000–9400 | *9880–9320* | This paper |  |
| OxA-26552^[[1]](#footnote-1)^ | Red deer antler mattock (S. no. 33) | building 38, underneath the building’s floor | 10,035±50 | –19.3 | 6.3 | – | 3.2 | – | – | 9852–9338 | *9790–9320* | This paper |  |
| OxA-25092 | Human right or left femur | Burial 60, primary, adult male (?), extended perpendicular to the Danube | 9970±45 | –19.1 | 15.0 | 5.9 | 3.2 | 78% | 9549±71 (method 1)  9530±64 (method 2) | 9209–8721 (1)  9176–8657 (2) | *9220–8700 (95.4%)*  *or*  *8670–8650 (0.5%)* | 5 |  |
| OxA-16072 | LV12, red deer right M_2_ | Beneath the floor of building 47’ (1315a) (09/10/1970) | 9850±50 | –20.2 | 7.8 | – | 3.2 | – | – | 9440–9241 | *9450–9230* | 6 |  |
| OxA-16004 | LV10, red deer metatarsus | Between the floors of building 47 and 47’, on the floor of Building 47’ (1314a) (09/10/1970) | 9730±50 | –20.3 | 6.0 | – | 3.4 | – | – | 9294–8928 | *9310–9120 (86.6%)*  or  *9010–8910 (8.1%)*  or  *8900–8860 (0.8%)* | 6 |  |
| OxA-16076 | LV30, red deer antler | Building 54, floor (October 1967) | 9750±45 | –19.6 | 6.1 | – | 3.2 | – | – | 9297–9152 | *9310–9130 (93.7%)*  or  *8980–8930 (1.7%)* | 6 |  |
| BA-10651 | Human rib | Burial 50, primary, adult male, extended supine with flexed lower limbs at the knees (raised), perpendicular to the Danube | 9455±38 | –19.5 | 14.2 | 4.8 | – | 69% | 9082±62 (method 1)  9015±59 (method 2) | 8532–8208 (1)  8340–7961 (2) | *8550–8200 (94.3%)*  or  *8110–8090 (0.3%)*  or  *8040–8000 (0.8%)* | 7 |  |
| OxA-25215 | Human left femur | Burial 69, adult male, seating w/crossed legs | 9089±38 | –19.3 | 14.6 | – | 3.2 | 73% | 8695±64 (method 1)  8649±59 (method 2) | 7940–7591 (1)  7933–7573 (2) | *7970–7580* | 5 |  |
| OxA-26551 | Red deer antler intermediate piece/tool (BB-23) (S. no. 20) | Quad. A/11, spit 6 (801) | 8910±45 | –21.2 | 9.7 | – | 3.1 | – | – | 8251–7941 | *8260–7910 (93.4%)*  or  *7900–7840 (2.0%)* | This paper |  |
| OxA-26553 | Red deer, pointed tool on a proximal end of a metapodial (BB-106) (S. no. 25) | Quad. C/XV, spit 19 (1058b) (23/08/1969) | 8840±45 | –22.8 | 6.8 | – | 3.2 | – | – | 8208–7760 | *8220–7750* | This paper |  |
| OxA-24771 | Red deer antler diffused-end tool (punch) (S. no. 32) | Building 19, beneath the building’s floor | 8871±38 | –21.1 | 5.5 | – | 3.1 | – | – | 8224–7837 | *8230–7830* | This paper |  |
| OxA-16071 | LV6, red deer modified antler | Building 26’, floor (bb-1303a) (31/08/1970) | 8855±40 | –21.4 | 6.2 | – | 3.2 | – | – | 8218–7794 | *8230–7780 (95.2%)*  or  *7770–7760 (0.2%)* | 6 |  |
| OxA-8610 | Long bone of a large-size ungulate | Building 23, beneath the floor (1299c) | 8770±60 | –21.6 | 4.7 | – | 3.3 | – | – | 8200–7600 | *8200–8110 (7.1%)*  or  *8100–8030 (2.5%)*  or  *8010–7600 (85.8%)* | 8 |  |
| AA-57781 | Human mandible | Burial 22, old adult, in association with hearth “a” | 8814±60 | –20.0 | 14.4 | – | – | 71% | 8431±78 (method 1)  8374±75 (method 2) | 7596–7317 (1)  7580–7191 (2) | *7600–7320* | 7 |  |
| OxA-16074 | LV22, shed red deer antler | Occupation zone around hearth “a” (268/1) (02/10/1967) | 8645±40 | –20.3 | 5.9 | – | 3.2 | – | – | 7740–7587 | *7750–7580* | 6 |  |
| OxA-26549 | Wild boar tusk pointed–edged tool/burin (BB-187) | Space between buildings 40 and 41 [zone around hearth “a”] (02/10/1967) | 8659±45 | –22.0 | 7.7 | – | 3.2 | – | – | 7754–7596  Weighted mean:  8685±32  X2-Test: df=1 T=0.6(5% 3.8) | *7790–7590* | This paper |  |
| OxA-26550 |  |  | 8710±45 | –22.0 | 8.1 | – | 3.2 | – | – |  |  | This paper |  |
| OxA-24813 | LV34, red deer bone | Beneath building 34 (1307/5) | 8640±40 | –22.1 | 5.2 | – | 3.1 | – | – | 7740–7580 | *7790–7770 (0.4%)*  or  *7760–7570 (95.0%)* | This paper |  |
| OxA-24812 | Brown bear, humerus, from a partially articulated skeleton with butchering cutmarks (BB-30/7) | Building 31, beneath the floor | 8410±39 | –19.7 | 6.8 | – | 3.1 | – | – | 7574–7359 | *7580–7360* | This paper |  |
| OxA-26548 | Wild boar tusk pointed–edged tool/burin (BB-112) (S. no. 47) | Quad. b/13, stone construction (1028b) (16/08/1968) | 8265±45 | –21.1 | 9.8 | – | 3.3 | – | – | 7476–7143 | *7570–7120 (95.3%)*  or  *7110–7080 (0.2%)* | This paper |  |
| **Lepenski Vir I-II contexts (Mesolithic-Neolithic Transition phase)** | | | | | | | | | | | | | |
| *Building 37* | | | | | | | | | | | | | |
| Bln-649 | LV5/67, charcoal (*Quercus* sp.), large timber beam | Building 37, floor | 6800±100 | – | – | – | – | – | – | 5882–5638  Weighted mean:  6860±64  X2-Test: df=2 T=0.6(5% 6.0) | *6100–5940*  *(poor agreement 6.3%)* | 9 |  |
| Bln-678 |  |  | 6900±100 | – | – | – | – | – | – |  |  |  |  |
| BM-379 |  |  | 6900±150 | – | – | – | – | – | – |  |  |  |  |
| OxA-32886 | Charcoal, *Quercus* sp. (S. no. 9) | Wooden beam from building 37 (June 1967) | 7156±36 | –25.3 | – | – | – | – | – | 6074–6000  Weighted mean:  7174±26  X2-Test: df=1 T=0.5(5% 3.8) | *6080–6000* | This paper |  |
| OxA-32887 |  |  | 7191±35 | –24.3 | – | – | – | – | – |  |  | This paper |  |
| OxA-16082 | LV20, bone tool (inv. 673) | Building 37, floor | 7138±37 | –20.6 | 5.9 | – | 3.2 | – | – | 6071–5922 | *6070–5980* | 6 |  |
| *Building 62* | | | | | | | | | | | | | |
| KN-405 | Charcoal (possibly *Quercus* sp.) | Building 62, from a timber beam over the hearth of building 62 | 7430±160 | – | – | – | – | – | – | 6595–6006 | *6140–5980* | 9 |  |
| OxA-32865 | Charcoal, *Cornus* sp. | Building 62, in front of the hearth, toward side C-D (charcoal 5) (29/07/1968) | 7176±36 | –25.4 | – | – | – | – | – | 6110–5980 | *6090–5990* | This paper |  |
| *Buildings 20, 33 and 32* | | | | | | | | | | | | | |
| P-1598 | Charcoal | Building 32, hearth of the building | 6814±69 | – | – | – | – | – | – | 5872–5571 | *6080–5940*  *(poor agreement 3.8%)* | 9 |  |
| OxA-15998 | LV1, roe deer metacarpus | Between the floors of buildings 20 and 33, on the floor of building 20 in corner D (1082) (30/08/1968) | 7280±45 | –22.1 | 7.9 | – | 3.2 | – | – | 6231–6056 | *6140–6030* | 6 |  |
| OxA-8725 | Fish vertebra | Between the floors of buildings 20 and 33, on the floor of building 20 in corner D | 7600±90 | –16.9 | 9.7 | – | 3.3 | – | 7380±93 (method 2) | 6420–6060 (2) | *6140–6020* | 8 |  |
| OxA-15999 | LV2, red deer right proximal metatarsus | Building 32, from the floor (1090/4) (1968) | 7111±40 | –20.8 | 5.4 | – | 3.4 | – | – | 6061–5902 | *6070–5950* | 6 |  |
| *Buildings 26 and 26’* | | | | | | | | | | | | | |
| OxA-16000 | LV4, red deer skull | Building 26, floor, quad. A/VI (143) (22/07/1967) | 7070±40 | –21.5 | 6.7 | – | 3.4 | – | – | 6023–5849 | *6030–5940* | 6 |  |
| OxA-16001 | LV5, red deer vertebra | Between the floors of buildings 26 and 26’, spit 1 below the floor of building 26 (1320a) (29/08/1970) | 7235±40 | –20.6 | 5.5 | – | 3.3 | – | – | 6200–6002  Weighted mean:  7198±29  X2-Test: df=1 T=1.8(5% 3.8) | *6100–6000* | 6 |  |
| OxA-16002 |  |  | 7160±40 | –20.1 | 6.4 | – | 3.2 | – | – |  |  | 6 |  |
| *Buildings 47 and 47’ and Burial 122* | | | | | | | | | | | | | |
| UCLA-1407 | Charcoal from a timber beam | Building 47, timber beam found lying along the left side | 6970±60 | – | – | – | – | – | – | 5983–5736 | *6010–5940* | 9 |  |
| OxA-16005 | LV11, skull fragment | Burial 122, disarticulated 15–18 year-old (M^3^ erupting) skull found between the floors of buildings 47 and 47’ (09/10/1970) | 7190±45 | –19.5 | 9.5 | – | 3.3 | 17% | 7098±47 (method 1) | 6102–5996  Weighted mean:  7190±30  X2-Test: df=1 T=0.0(5% 3.8) | *6090–6000* | 6 |  |
| OxA-16006 |  |  | 7190±40 | –19.3 | 9.3 | – | 3.3 | 15% | 7109±41 (method 1) |  |  |  |  |
| *Buildings 51, 57/XLIV and Burial 19(3)* | | | | | | | | | | | | | |
| OxA-16008 | LV17, unfused human humerus | Burial 19(3), disarticulated remains of a 10-year-old child, over the floor of building 57/XLIV | 7205±40 | –18.1 | 10.2 | 9.9 | 3.3 | 21% | 7092±42 (method 1)  6985±46 (method 2) | 6050–5892 (1)  5982–5756 (2) | *6050–5940* | 6 |  |
| OxA-16081 | LV15, bone tool (inv. 689) | Stone construction above the floor of building 57/XLIV (05/10/1967) | 7219±37 | –21.3 | 6.3 | – | 3.2 | – | – | 6210–6012 | *6100–6000* | 6 |  |
| OxA-16073 | LV16, roe deer right M_2_, | Floor of building 57/XLIV (558/1) (04/07/1968) | 7125±40 | –21.5 | 6.5 | – | 3.2 | – | – | 6068–5913 | *6070–5970* | 6 |  |
| OxA-8618 | Large-size ungulate long bone | Floor of the rear area of building 51 (1313,a,c) beneath building 57/XLIV | 7200±60 | –21.2 | 3.9 | – | 3.3 | – | – | 6220–5920 | *6130–6030* | 8 |  |
| Bln-652 | LV8/67, charcoal (*Ulmus* sp.) | Timber beam found on the floor of building 51, along its left side | 6620±100 | – | – | – | – | – | – | 5720–5376 | *6120–5950*  *(poor agreement 5.4%)* | 9 |  |
| *Building 34 and Burial 26* | | | | | | | | | | | | | |
| Bln-650 | LV6/67, charcoal (*Quercus* sp.) | Timber beam found on the floor of building 34 beneath the floor of building 43 | 6820±100 | – | – | – | – | – | – | 5973–5556 | *6020–5940*  *(poor agreement 15.3%)* | 9 |  |
| AA-57782 | Human mandible | Burial 26, adult male, extended inhumation, parallel to the Danube, head downstream direction, interred through the floor of building 34 | 7332±50 | –26.5 | 11.5 | 6.6 | – | 39% | 7122±57 (method 1)  7112±55 (method 2) | 6016–5896  Weighted mean:  7077±32  X2-Test: df=1 T=0.9(5% 3.8) | *6030–5930* | 7 |  |
| OxA-25206 | Human right tibia |  | 7161±34 | –20.3 | 9.7 | – | 3.2 | 19% | 7058±37 (method 1)  6941±41 (method 2) |  |  | 5 |  |
| OxA-16009 | LV19, red deer mandible | Floor of building 34 (517, 483) (18/10/1967) | 7165±40 | –22.3 | 7.9 | – | 3.1 | – | – | 6100–5925 | *6090–5980* | 6 |  |
| *Building 24 and Burials 94 and 100* | | | | | | | | | | | | | |
| OxA-34519 | Human unfused long bone epiphysis | Burial 100, primary disturbed, c. 8 years old, extended supine, articulated beneath the floor of building 24 (26/08/1970) | 7695±40 | –18.0 | 14.0 | 8.9 | – | 67% | 7333±62 (method 1)  7255±60 (method 2) | 6362–6065 (1)  6231–6016 (2) | *6140–6060* | This paper |  |
| OxA-16010 | LV25, human short bone | Burial 94, primary, neonate, in the rear of building 24 found beneath the floor level (15/10/1970) | 7520±40 | –18.1 | 19.5 | 11.5 | 3.2 | 100% | 6980±81 (method 1)  7080±60 (method 2) | 6008–5722 (1)  6066–5833 (2) | *6050–5940* | 6 |  |
| OxA-X-2176-18 | LV24, red deer right M_1/2_ | Area around the hearth of building 24 (1300a) (02/09/1970) | 7285±45 | –21.5 | 7.0 | – | 3.2 | – | – | 6231–6060 | *6130–6020* | 6 |  |
| *Building 65/XXXV and Burial 54c, 54d, and 54e* | | | | | | | | | | | | | |
| OxA-25210 | Human left femur | Burial 54e, adult female (?), extended parallel to the Danube, head downstream direction | 7474±35 | –19.4 | 13.3 | 5.93 | 3.2 | 59% | 7155±54 (method 1)  7034±57 (method 2) | 6205–5907 (1)  6018–5783 (2) | *6100–5970* | 5 |  |
| OxA-25209 | Human left femur | Burial 54c, adult female, extended parallel to the Danube, head downstream direction | 7461±35 | –19.0 | 12.8 | 8.0 | 3.2 | 54% | 7169±52 (method 1)  7241±42 (method 2) | 6208–5922 (1)  6216–6026 (2) | *6100–5980* | 5 |  |
| AA-57783 | Human skull fragment | Burial 54d, of old adult female placed over the floor of building 65, partly disturbed | 7494±51 | –20.0 | 15.1 | 8.8 | – | 79% | 7067±55 (method 1)  7054±68 (method 2) | 6088–5918  Weighted mean:  7149±43  X-Test fails at 5%  X2-Test: df=1 T=5.361(5% 3.8) | *6100–5960* | 7 |  |
| OxA-25213 | Human left femur |  | 7717±35 | –18.1 | 15.4 | – | 3.2 | 83% | 7269±68 (method 1)  7277±57 (method 2) |  |  | 5 |  |
| OxA-X-2176-19 | LV26, red deer metacarpus | Rear area of the floor of building 65/XXXV (1329a) (30/09/1970) | 7314±40 | –20.7 | 6.5 | – | 3.2 | – | – | 6240–6070 | *6140–6050* | 6 |  |
| *Buildings 21 and 22 and* *Burials 7/I-a and 7/II-b* | | | | | | | | | | | | | |
| OxA-16537 | LV9, red deer skull | Structured deposition of red deer skull with antlers in burial 7, interred through the floor of building 21 (524) (20/10/1967) | 6924±37 | –21.9 | 7.7 | – | 3.4 | – | – | 5887–5730 | *6050–5940*  *(poor agreement 5.8%)* | 6 |  |
| OxA-32933 | Aurochs skull | Structured deposition of aurochs skull in burial 7, interred through the floor of Building 21 (20/10/1967) | 7133±37 | –21.0 | 11.9 | – | 3.2 | – | – | 6068–5920 | *6060–5970* | This paper |  |
| AA-57779 | Human rib | Burial 7/I-a, articulated male adult burial interred through the floor of building 21 | 7368±74 | –18.9 | 11.5 | 10.9 | – | 39% | 7157±79 (method 1)  7148±78 (method 2) | 6208–5992  Weighted mean:  7197±44  X2-Test: df=2 T=0.4(5% 6.0) | *6070–5990* | 7 |  |
| OxA-25204 | Human right femur |  | 7710±35 | –18.3 | 16.1 | – | 3.2 | 90% | 7224±72 (method 1)  7270±57 (method 2) |  |  | 5 |  |
| OxA-25205 |  |  | 7689±37 | –18.1 | 16.1 | – | 3.2 | 90% | 7203±73 (method 1)  7249±58 (method 2) |  |  | 5 |  |
| AA-57780 | Human skull fragment | Burial 7/II-b, disarticulated adult human skull placed on the left shoulder of burial 7/I-a | 7512±71 | –20.0 | 16.0 | – | – | 89% | 7031±95 (method 1)  7072±84 (method 2) | 6066–5726 (1)  6080–5746 (2) | *6050–5940* | 7 |  |
| OxA-16075 | LV28, red deer antler | Structured deposition of red deer skull with antlers on the floor of building 22 (261) (August 1967) | 7157±39 | –21.9 | 6.2 | – | 3.2 | – | – | 6086–5927 | *6100–6010* | 6 |  |
| *Building 54* | | | | | | | | | | | | | |
| Z-115 | Charcoal | Timber beam in building’s corner A | 6984±94 | – | – | – | – | – | – | 6031–5676 | *6070–5940* | 9 |  |
| Bln-653 | LV9/67, charcoal (*Quercus* sp.) | Timber beam in corner A underneath a stone | 7040±100 | – | – | – | – | – | – | 6085–5720 | *6080–5940* | 9 |  |
| Bln-738 | LV12/68, charcoal (*Quercus* sp.) | from the building’s hearth | 7225±100 | – | – | – | – | – | – | 6355–5898 | *6120–5970* | 9 |  |
| KN-407 | Charcoal | Possibly from the building’s hearth | 7280±160 | – | – | – | – | – | – | 6452–5846 | *6120–5960* | 9 |  |
| OxA-26547 | Wild boar tusk pointed–edged tool/burin (BB-209) (S. no. 29) | Building 54, underneath the building’s floor (1317bc) | 7396±40 | –22.7 | 9.6 | – | 3.2 | – | – | 6393–6116 | *6150–6060*  *(poor agreement 9.7%)* | This paper |  |
| *Building 27* | | | | | | | | | | | | | |
| KN-406 | LV1, Charcoal | Probably from the building’s hearth | 7210±200 | – | – | – | – | – | – | 6452–5724 | *6130–5950* | 9 |  |
| OxA-16077 | LV31, red deer D_4_ | Building floor – section above the rear part of the building (1304a) (03/09/1970) | 7225±40 | –24.4 | 8.7 | – | 3.2 | – | – | 6210–6017 | *6120–6010* | 6 |  |
| *Buildings 35 and 36 – between two floors* | | | | | | | | | | | | | |
| OxA-16003 | LV8, pig phalanx III | Between the floors of Buildings 35 and 36, in the hearth of building 36 (1036) (23/08/1968) | 7170±40 | –20.3 | 7.0 | – | 3.3 | – | – | 6198–5928 | *6090–5980* | 6 |  |
| Bln-740a | LV13/68, charcoal (*Quercus* sp.) | from timber beam lying on the floor of building 36 beneath the floor of building 35 | 7310±100 | – | – | – | – | – | – | 6376–6060  Weighted mean:  7335±71  X2-Test: df=1 T=0.1(5% 3.8) | *6140–6020* | 9 |  |
| Bln-740b |  |  | 7360±100 | – | – | – | – | – | – |  |  |  |  |
| *Building 4* |  |  |  |  |  |  |  |  |  |  |  |  |  |
| OxA-16084 | Bone tool (inv. 349), large mammal bone | Building 4, from the building’s floor | 7285±37 | –21.2 | 6.9 | – | 3.2 | – | – | 6226–6068 | *6130–6030* | 6 |  |
| OxA-27901 | Fish hook (inv. 350, 10/07/1967), large mammal bone | Building 4 (block B), from the building’s floor | 7207±35 | –20.0 | 9.8 | – | 3.2 | – | – | 6206–6006 | *6110–6000* | This paper |  |
| OxA-26554 | Bone tool – tapered base (BB-193), dog bone based on isotope values and ZooMS | Building 4, beneath the floor | 7710±40 | –18.0 | 12.0 | – | 3.2 | 46% | 7462±44 (method 1)  7490±46 (method 2) | 6421–6241 (1)  6436–6250 (2) | *6140*–*6060*  *(poor agreement 4%)* | This paper |  |
| *Building 40* | | | | | | | | | | | | | |
| OxA-25211 | Human right femur | Burial 61, beneath the floor of building 40, child 2-6 years old, primary extended parallel with the Danube, head downstream | 7670±35 | –19.0 | 16.1 | – | 3.2 | 90% | 7184±72 (method 1)  7230±57 (method 2) | 6223–5916 (1)  6220–6010 (2) | *6120–5980* | 5 |  |
| OxA-34968 | Human mandible, right condylar process | Burial 21, disarticulated mandible embedded in the floor of building 40 next to its hearth along with a stone plaque to form ∀-shaped support | 7193±39 | –20.1 | 9.9 | – | 3.2 | 21% | 7080±42 (method 1)  6973±45 (method 2) | 6050–5880 (1)  5990–5740 (2) | *6030–5940* | This paper |  |
| *Only one measurement per building* | | | | | | | | | | | | | |
| Bln-576 | LV2/66, charcoal (*Quercus* sp.) | Building 16**,** A thin layer of charcoal on the floor | 6820±100 | – | – | – | – | – | – | 5972–5556 | *6080–5940*  *(poor agreement 14.3%)* | 99 |  |
| Bln-575 | LV1/66, charcoal (*Quercus* sp.) | Building 1, floor, SE area of the building, between two stone slabs | 6860±100 | – | – | – | – | – | – | 5982–5574 | *6060–5940*  *(poor agreement 27.8%)* | 9 |  |
| Bln-647 | LV3/67, charcoal (*Quercus* sp.) | Building 9, Timber beam in the area covered by the floor of building 8 | 6845±100 | – | – | – | – | – | – | 5979–5566 | *6070–5930*  *(poor agreement 22.1%)* | 9 |  |
| OxA-16078 | Red deer skull (S. LV32) | Building 28, from the building’s floor (273) (03/10/1967) | 7191±40 | –21.4 | 8.2 | – | 3.2 | – | – | 6205–5990 | *6100–5990* | 6 |  |
| OxA-16083 | Bone tool (inv. 125), medium size ungulate (LV33) | Building 5, from the building’s floor (12/08/1965) | 7059±36 | –20.7 | 6.0 | – | 3.2 | – | – | 6014–5850 | *6020–5940* | 6 |  |
| OxA-25214 | Human left femur | Burial 45b in the pile of disarticulated bones on the floor of building 61/XXXIV, associated with a robust male disarticulated skull | 7759±33 | –18.9 | 15.8 | – | 3.2 | 87% | 7289±69 (method 1)  7319±56 (method 2) | 6351–6015 (1)  6356–6056 (2) | *6140–6010* | 5 |  |
| *Phase* *I-II contexts outside of trapezoidal buildings* | | | | | | | | | | | | | |
| OxA-25090 | Human right femur | Burial 14 in burial pit, sq. A/VIII, primary, adult female (?), extended parallel with the Danube, head downstream, one red deer antler tool | 7701±37 | –18.9 | 15.9 | 9.9 | 3.2 | 88% | 7226±72 (method 1)  7261±58 (method 2) | 6236–5985 (1)  6231–6021 (2) | *6130–5980* | 5 |  |
| BA-10651 | Human skull fragment | Burial 93, primary, adult female (?), extended supine, parallel with the Danube head downstream; discoid beads and antler (phase I-II) | 7550±70 | –18.5 | 15.1 | 10.8 | – | 79% | 7123±89 (method 1)  7110±83 (method 2) | 6214–5812 (1)  6208–5801 (2) | *6110–5940* | 7 |  |
| OxA-25089 | Human tibia | Burial 89a, primary, subadult?, extended parallel with the Danube partly disturbed burial with an aurochs skull above the head of the deceased, next to section *f* | 7521±36 | –18.4 | 15.9 | – | 3.2 | 88% | 7046±71 (method 1)  7081±58 (method 2) | 6049–5768 (1)  6061–5842 (2) | *6060–5940* | 5 |  |
| OxA-5830 | Human right humerus | Burial 44, adult, disarticulated human remains | 7590±90 | –18.9 | 15.3 | – | – | 81% | 7153±106 (method 1)  7150±101 (method 2) | 6236–5796 (1)  6232–5810 (2) | *6120–5950* | 10 |  |
| OxA-5827 | Human left humerus | Burial 31a, male (?), disarticulated human bones in Pit 2 in square a/VII | 7770±90 | –18.7 | 15.7 | – | – | 85% | 7311±108 (method 1)  7230±101 (method 2) | 6406–6002 (1)  6359–5904 (2) | *6140–5990* | 10 |  |
| OxA-25091 | Human scapula | Burial 79a, adult male (?), disarticulated pile of cranial and postcranial bones (individuals 79a-c) in square e/4, spits 11–13 | 7605±38 | –18.7 | 16.1 | – | 3.2 | 90% | 7119±74 (method 1)  7165±59 (method 2) | 6206–5840 (1)  6210–5916 (2) | *6100–5940* | 5 |  |
| **Phase III (Early Neolithic)** | | | | | | | | | | | | | |
| Bln-654 | LV10/67, charcoal (*Quercus* sp.) | Building IX, i.e. occupation zone above the infill of building 37 | 6630±100 | – | – | – | – | – | – | 5724–5379 | *5920–5550*  *(poor agreement 53.3%)* | 9 |  |
| Bln-655 | LV11/67, Charcoal (*Quercus* sp.) | Building XXXII, occupation zone above the infill of building 48 | 6560±100 | – | – | – | – | – | – | 5658–5325 | *5940–5540*  *(poor agreement 39.7%)* | 9 |  |
| OxA-16007 | LV13, bone tool (inv. 336) | Stone construction above the level of building 8 (spit 7) (08/07/1966) | 7050±40 | –21.1 | 5.2 | – | 3.3 | – | – | 6009–5846 | *5960–5830* | 6 |  |
| OxA-16212 | LV37, domestic goat proximal metacarpus | Domed oven in quad. d/3, spit 6 (831a) (26/07/1968) | 7041±35 | –19.8 | 6.8 | – | 3.2 | – | – | 6000–5845 | *5960–5840* | 6 |  |
| OxA-16253 | LV38, Domestic goat mandible | Quad. C/XVI, spit 3 (16/08/1968) | 7008±38 | –20.7 | 7.1 | – | 3.2 | – | – | 5988–5799 | *5960–5790* | 6 |  |
| OxA-16213 | LV39, domestic cattle proximal metatarsus | Quad. c/I, spit 7 (unit 905a) (01/08/1968) | 7043±37 | –21.5 | 8.3 | – | 3.2 | – | – | 6002–5845 | *5960–5830* | 6 |  |
| OxA-16079 | LV35, domestic pig scapula | Pit 1, quad. a/VII, spit 9 (665) (12/07/1968) | 7037±39 | –20.2 | 9.3 | – | 3.2 | – | – | 6005–5841 | *5960–5810* | 6 |  |
| OxA-16211 | LV36, domestic cattle horncore | Pit 3, quad. a/VIII, spit 9 (674) (15/07/1968) | 7021±36 | –21.1 | 6.7 | – | 3.2 | – | – | 5996–5811 | *5960–5810* | 6 |  |
| OxA-16538 | LV14, unfused epiphysis of a medium-sized mammal (inv. 552) | Placed with crouched primary adult burial 5 (11/07/1966) | 7136±37 | –21.4 | 6.0 | – | 3.3 | – | – | 6070–5922 | *5960–5750*  *(poor agreement 21.4%)* | 6 |  |
| AA-58319 | Human skull fragment | Burial 8, primary, old adult female, crouched position, right side, over the floor of building 24 (bag 263) | 6825±51 | –21.2 | 10.2 | 5.1 | – | 25% | 6690 ±54 (method 1)  6605±56 (method 2) | 5844–5712  Weighted mean:  6887±32  X-Test fails at 5%  X2-Test: df=1 T=19.248(5% 3.8) | *5920–5740*  *(poor agreement 42.3%)* | 7 |  |
| OxA-25207 | Human left femur |  | 7097±36 | –19.9 | 9.9 |  | 3.2 | 21% | 6984±39 (method 1)  6877±43 (method 2) |  |  | 5 |  |
| OxA-25208 | Human left femur | Burial 9, primary, old adult female, crouched position, right side, over the floor of building 24 | 7120±34 | –19.6 | 11.8 | – | 3.2 | 42% | 6893±45 (method 1)  6900±41 (method 2) | 5888–5676 (1)  5882–5716 (2) | *5900–5700* | 5 |  |
| OxA-5828 | Human left femur | Burial 32, primary, adult female (?) in crouched position, right side | 7270±90 | –19.6 | 11.9 | 7 | – | 44% | 7032±95 (method 1)  7050±93 (method 2) | 6066–5727 (1)  6076–5731 (2) | *5960–5730* | 10 |  |
| OxA-5831 | Human femur or left tibia (?) | Burial 88, primary, adult female (?) in flexed position, right side, stone construction | 7130±90 | –20.2 | 10.5 | 6.13 | – | 28% | 6979±92 (method 1)  6910±93 (method 2) | 6025–5676 (1)  5984–5646 (2) | *5950–5720* | 10 |  |
| OxA-5829 | Human long bone (?) | Burial 35, adult female (?), disarticulated bones | 6910±90 | –19.7 | 11.2 | 4.34 | – | 35% | 6721±93 (method 1)  6690±93 (method 2) | 5787–5482 (1)  5748–5475 (2) | *5910–5570* | 10 |  |
| BA-10652 | Human skull fragment | Burial 73, primary, adult male (?), right crouched | 7265±30 | –19.5 | 12.8 | 8.8 | – | 54% | 6973±48 (method 1)  7045±38 (method 2) | 5981–5742 (1)  6004–5846 (2) | *5930–5740* | 7 |  |
| AA-58320 | Human skull fragment | Burial 17, primary, cranial fragment of possible young adult female, partly disturbed crouched inhumation | 7007±48 | –20.0 | 10.9 | – | – | 33% | 6829±53 (method 1)  6787±53 (method 2) | 5836–5632 (1)  5777–5616 (2) | *5890–5640* | 7 |  |
| **Post-Neolithic burials** | | | | | | | | | | | | | |
| OxA-25093 | Human right femur | Burial 2, primary, adult female (?), flexed | 5337±32 | –19.9 | 10.4 | – | 3.2 | 27% | 5191±37 (method 1) | 4220–3946 (1)  4313–4050 (no correction) | – | 5 |  |
| OxA-25217 | Human left ulna | Burial 18, adult, disarticulated remains | 1825±25 | –18.6 | 10.6 | – | 3.2 | 29% | 1668±32 (method 1) | AD 257–506 (1)  AD 125–251 (no correction) | – | 5 |  |
| OxA-25212 | Human left fibula | Burial “4”, adult, mixed bones? | 421±23 | –18.5 | 9.5 | – | 3.2 | 17% | 329±26 (method 1) | AD 1482–1643 (1)  AD 1432–1610 (no correction) | – | 5 |  |
| OxA-25216 | Human left femur | Burial 29, primary, adult male, extended supine | 426±23 | –18.6 | 9.6 | – | 3.2 | 18% | 329±26 (method 1) | AD 1482–1643 (1)  AD 1430–1607 (no correction) | – | 5 |  |
| OxA-25218 | Human right humerus | Burial 30, primary, adult male, extended supine | 427±23 | –18.7 | 10.4 | – | 3.2 | 27% | 281±30 (method 1) | AD 1497–1794 (1)  AD 1430–1487 (no correction) | – | 5 |  |
| OxA-25219 | Human right femur or right tibia | Burial 62, primary, adult male, extended supine | 389±23 | –18.8 | 9.3 | – | 3.2 | 15% | 308±25 (method 1) | AD 1492–1648 (1)  1442–1621 (no correction) | – | 5 |  |

**Supplementary Table S2**. Sample pretreatment, processing and measurement details.

| **Radiocarbon laboratory** | **Lab number** | **Pretreatment, processing and measurement details** | **Measurement method** |
| --- | --- | --- | --- |
| NSF-Arizona AMS Facility | AA-57779–83, AA-58319–20 | Probably as in Stafford *et al.*^11^ | Accelerator mass spectrometry |
| Berlin | Bln-575–6, Bln-647, Bln-649–50, Bln-653–5, Bln-678, Bln-738, Bln-740a–b | Kohl and Müller^12^; cf. Quitta^9^; acid/base wash; using the 5570 half-life; not corrected for fractionation | Gas proportional counting using acetylene |
| Laboratory of Quaternary Chronology, University of Peking, Beijing | BA-10651–3 | ? | Accelerator mass spectrometry |
| The British Museum | BM-379 | Gas proportional counting of acetylene as described in Barker and MacKey^13^ (see also ^14-15^) | ? |
| Labor für ^14^C-Datierung  Institut für Ur-und Frühgeschichte  Universität zu Köln | KN-405–7 | ? | ? |
| Oxford Radiocarbon Accelerator Unit | OxA-5827–5831; OxA-8610, OxA-8618, OxA-8725;  OxA-25089 (replaces OxA-11702); OxA-25090 (replaces OxA-11704); OxA-25091 (replaces OxA-11705); OxA-25092 (replaces OxA-11715); OxA-25093 (replaces OxA-11719); OxA-25204 (replaces OxA-11692); OxA-25205 (replaces OxA-12979); OxA-25206 (replaces OxA-11693); OxA-25207 (replaces OxA-11694); OxA-25208 (replaces OxA-11695); OxA-25209 (replaces OxA-11696); OxA-25210 (replaces OxA-11697); OxA-25211 (replaces OxA-11698); OxA-25212 (replaces OxA-11699); OxA-25213 (replaces OxA-11700); OxA-25214 (replaces OxA-11701); OxA-25215 (replaces OxA-11703); OxA-25216 (replaces OxA-11706); OxA-25217 (replaces OxA-11716); OxA-25218 (replaces OxA-11717); OxA-25219 (replaces OxA-11718);  OxA-15998–16002, OxA-16003–6; OxA-16007–10; OxA-16071–2; OxA-16074–6, OxA-16078–9, OxA-16081–4; OxA-16214–21; OxA-16537–8; OxA-16212 –13, OxA-16253; OxA-X-2176-18–19; OxA-24771; OxA-24812; OxA-26547–54; OxA-27901; OxA-32863-5; OxA-32886-7; OxA-34519; OxA-34968 | OxA-5827–31  — bone^16-19^  The rest:  — bone, tusk or teeth samples^20-24^ | Accelerator mass spectrometry |
| University of Pennsylvania | P-1598 | ? | ? |
| Institute of Geophysics and Planetary Physics  University of California | UCLA-1407 | ? | ? |
| Zagreb | Z-115 | Srdoč *et al.*^25^; Srdoč *et al.*^26^; AAA pretreatment; not corrected for fractionation | Gas proportional counting using methane |

Note: In Model 2 (Fig. 7 in the main text), the following samples yielded outlying values of significance: OxA-16537 (70%), OxA-26547 (35%), OxA-26554 (72%) and AA-58319 (100%). For Model 3 outliers were OxA-16537 (34%) and AA-58319 (100%).

**Supplementary text 1: Methods used for correcting fish-derived offset on human bones**

Method 1 uses the age offset between paired herbivore and human radiocarbon measurements from Schela Cladovei and assumed δ^15^N dietary end points to estimate the percentage of freshwater contribution in ancient human populations. Method 2 uses an average of the data to estimate the offset and estimate offsets proportional to the percentage contribution of freshwater diet. Both these methods are relatively crude and have limitations^1,27-28^. In any case, the calibrated results produced from either of these approaches must be understood as relatively crude; they are based on limited data, on data from a proximal site rather than Lepenski Vir itself, they use estimates of dietary end points which may not reflect the subtlety of the subsistence strategies at Lepenski Vir, and they assume a uniform offset over time. The nature of the dietary offset at the site urgently needs to be the subject of further work.

**Supplementary text 2: ZooMS analysis of OxA-26554**

Collagen peptide mass fingerprinting (also known as Zooarchaeology by Mass Spectrometry; ZooMS) was carried out following a modified form of the approach published by van der Sluis *et al.*^29^. In brief, this involved the ultrafiltration of soluble proteins following overnight decalcification in 0.6 M hydrochloric acid into 50 mM ammonium bicarbonate and overnight digestion with sequencing grade trypsin (Promega, UK). Samples were analysed using a Bruker Ultraflex II Matrix Assisted Laser Desorption Ionization Time of Flight (MALDI-ToF) mass spectrometer at the University of Manchester, UK with the acquisition of 2,000 laser shots. The resultant mass spectra were then searched for taxonomically informative markers through comparison with the set published by Buckley *et al*.^30^.

**
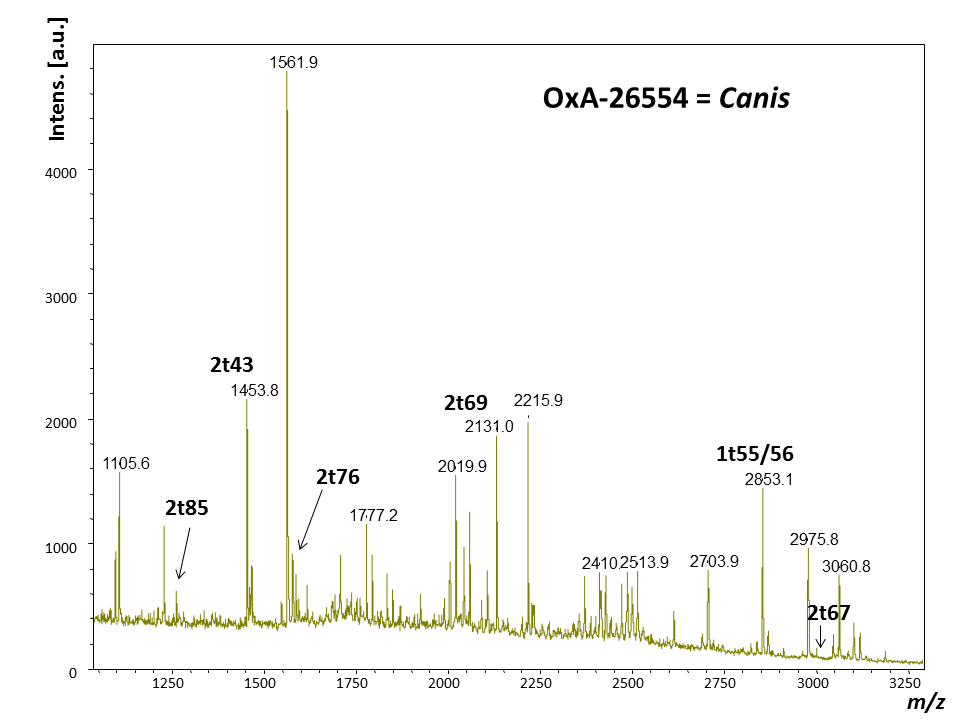
**

**Supplementary Figure S1.** MALDI-ToF mass spectrum showing peptide mass fingerprint of collagen tryptic digest identified by at least six well-established biomarkers for *Canis* sp. (labelling following Buckley^31^).

**Supplementary text 3: Bayesian statistical modelling**

Although Bayesian methods have been available to archaeologists^32^ for some time it is only recently that they have been applied more extensively in archaeology, particularly in a recent project on the chronology of the south English long barrows^33^. While the essence of Bayesian methods is simple – one incorporates all prior information about a problem into its statistical solution – the practicalities are more complex, often calling for numerical solution of complex problems. Markov Chain Monte Carlo methods – a simulation-based approach to deriving the solution to Bayesian problems – have been behind the recent boom in applications through their incorporation into programmes such as OxCal and WinBUGS.

In an archaeological context the prior information often concerns stratigraphic relationships, and that is the case at Lepenski Vir. If we are sure that a certain sample predates another then we can incorporate that information in order to provide more accurate estimates of sample ages. In addition to prior information, Bayesian approaches also often propose formal statistical models of the data generation process: for example, that a site had a specified number of phases or that samples were laid down according to a uniform distribution. Such phases have beginnings and ends – dates that are likely before our earliest sample from a phase and after our latest – and the approach allows us to estimate the probability densities of these parameters.

**Supplementary Figure S2.** Excavation area at Lepenski Vir and architectural features with marked samples of occupation residues from contexts found beneath the floors of trapezoidal buildings and in association with Early and Middle Mesolithic features (phases Proto-Lepenski Vir 1 and 2). Shaded areas indicate contexts with available faunal remains and areas that have been AMS-dated.


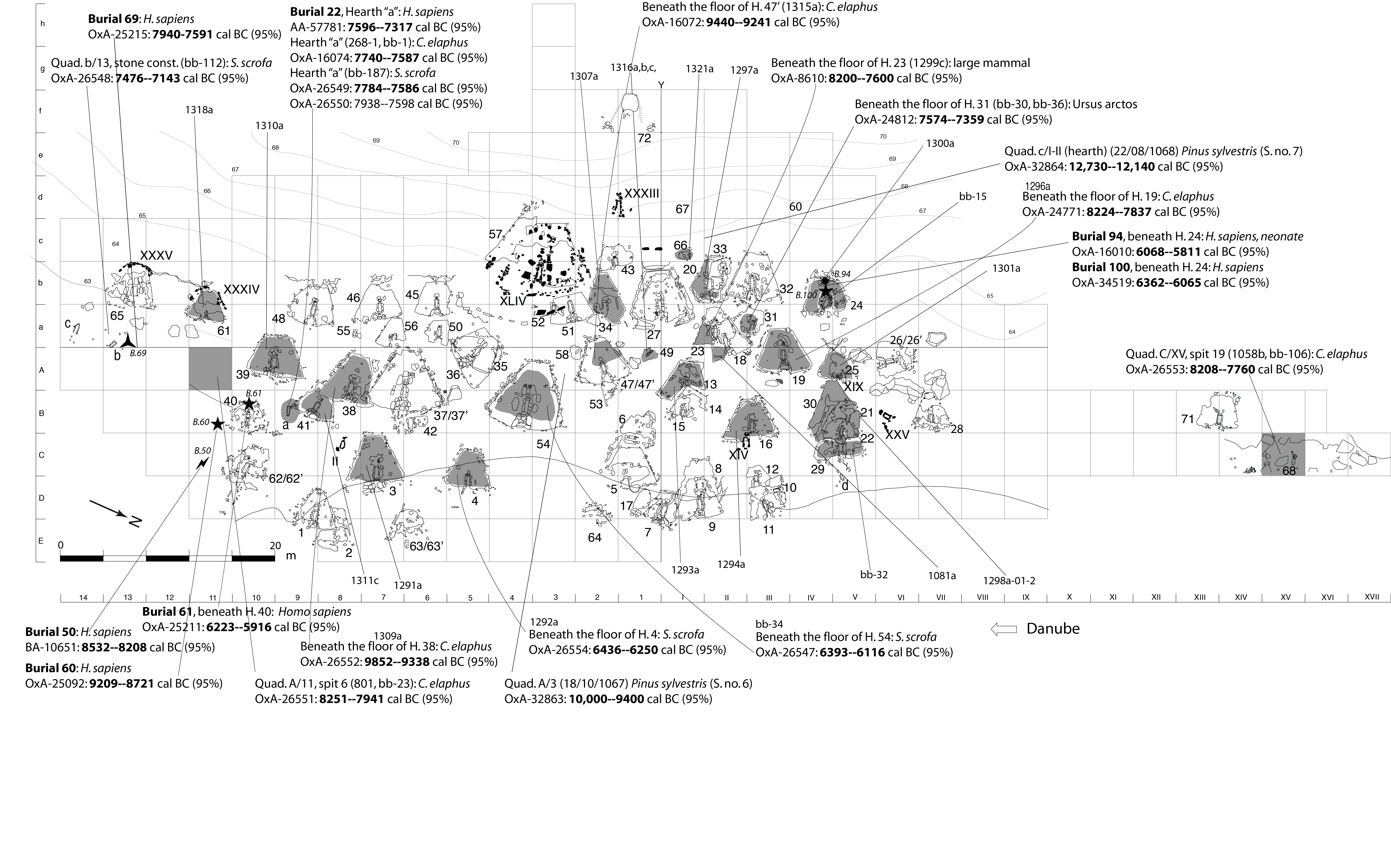


**Supplementary Figure S3.** Excavation area at Lepenski Vir and architectural features with marked samples from contexts found in direct contextual association with the floors of trapezoidal buildings (phase Lepenski Vir I-II). These contexts include occupation residues left on trapezoidal building floors, structured deposition of red deer skulls and antlers and burials cut through trapezoidal building floors or deposited directly on building floors placed as extended supine inhumations, denoting a typical Mesolithic burial rite; some of these contexts relate to deposits/“packing” between superposed limestone floors of two buildings and are considered stratigraphically “closed” contexts. Light shaded areas indicate contexts with available faunal remains; dark shaded areas designate contexts between two superimposed floors with available faunal remains.


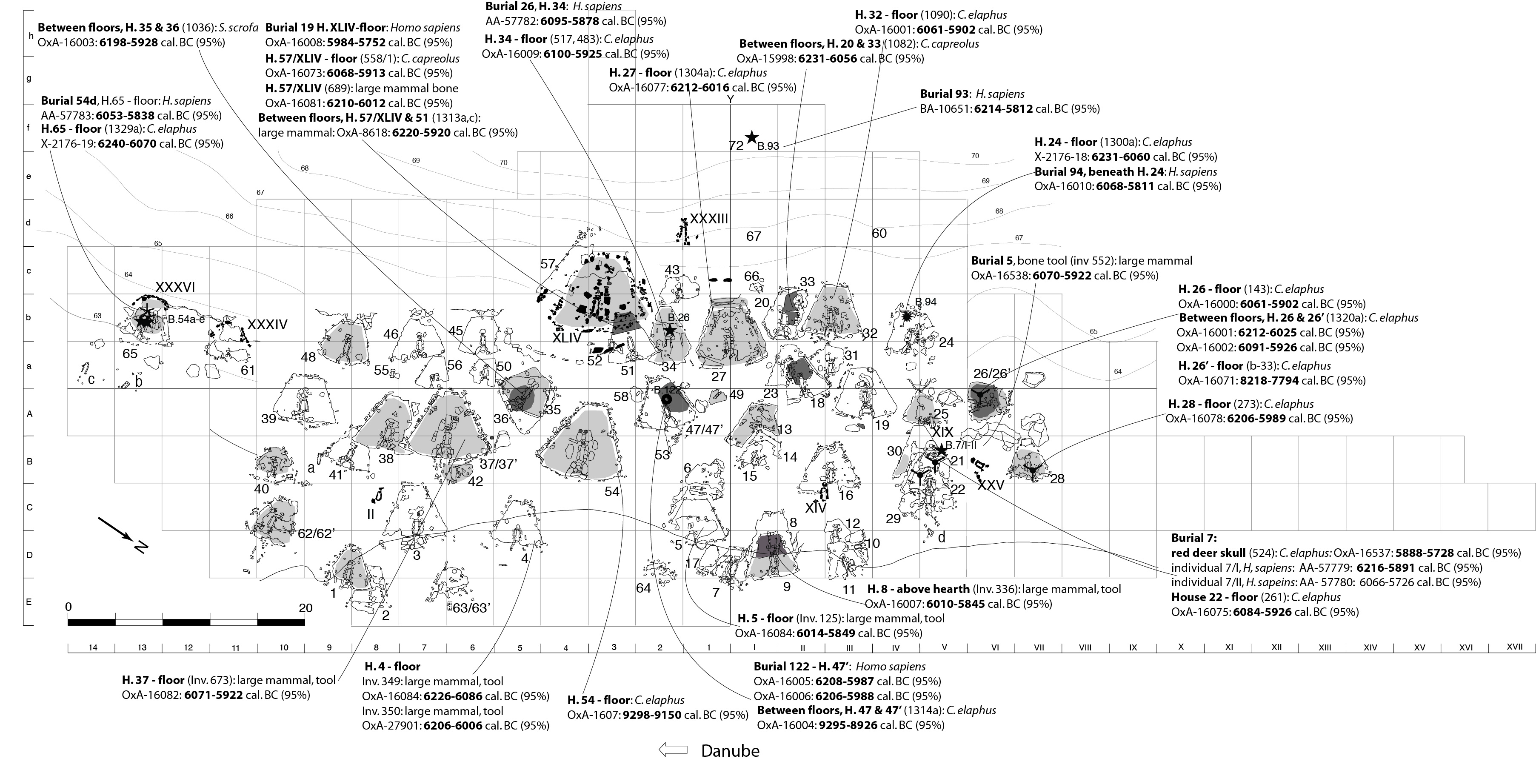


**Supplementary Figure S4.** Excavation area at Lepenski Vir and architectural features with marked samples largely found outside the space and deposits of trapezoidal buildings and related to the Early Neolithic occupation of the site (phase Lepenski Vir III); five dated samples come from domesticated animal bones and four dated burials are places as crouched/flexed inhumations, denoting a typical Neolithic burial rite. Shaded areas indicate contexts with available faunal remains that have been AMS-dated.


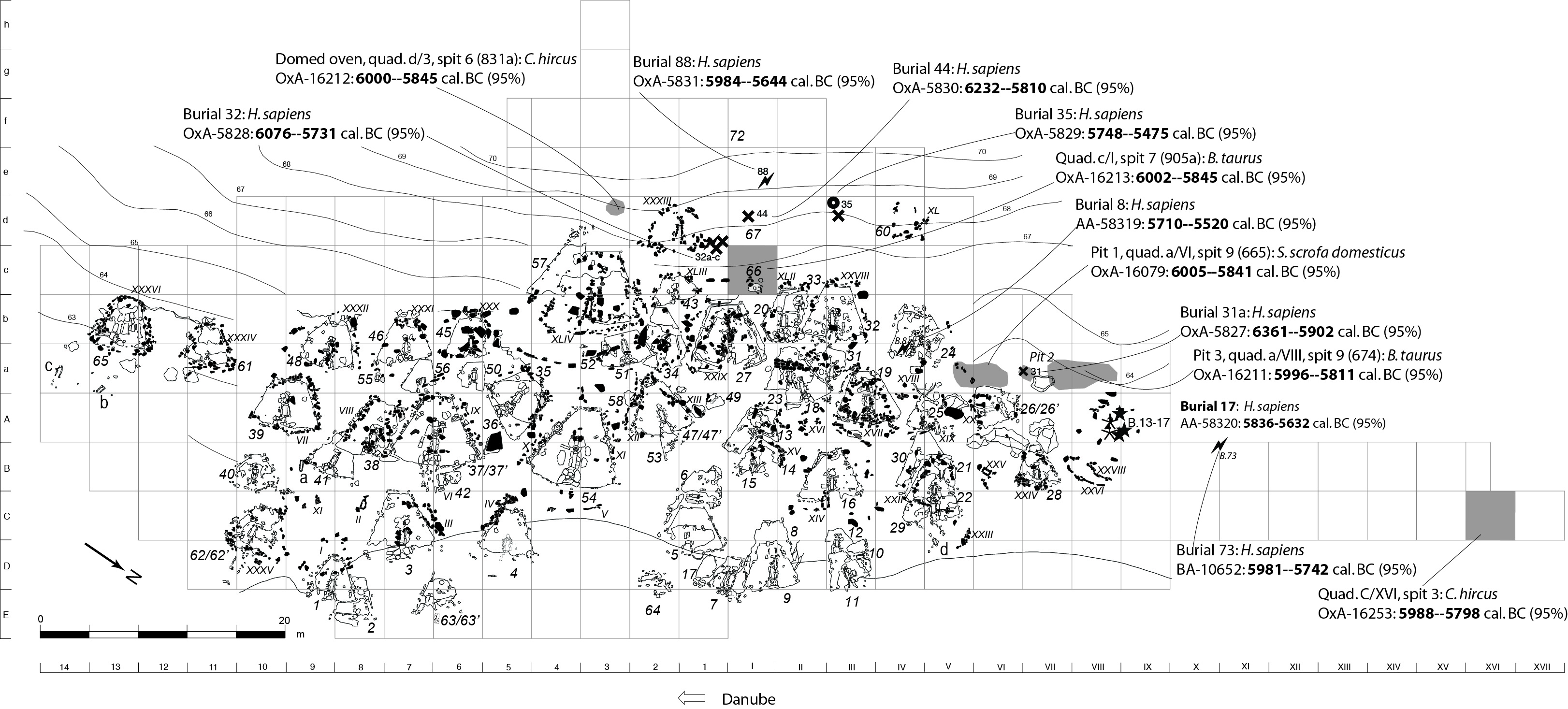


**A**

**Supplementary Figure S5**: Different sets of radiocarbon measurements being modelled for Lepenski Vir:

(A) Model 1: All available radiocarbon measurements (N=103);

(B) Model 3: Only articulated human and animal remains (N=35). For the radiocarbon measurements, distributions in outline are the results of simple radiocarbon calibrations, solid distributions are the output from the chronological model. The large square brackets and OxCal v. 4.3.2 CQL2 keywords define the overall model exactly (see the CQL codes below). Blue: human bone; magenta: animal bones; green: charcoal.

**B**

**Supplementary Figure S6**: Stratigraphy of Lepenski Vir as seen on section 5 along line b of the excavation grid that cuts through the floors of buildings 27, 20, 33, and 32 (quadrants b/1 to b/IV); (A) after plan no. 277 (Center for Archaeological Research, Faculty of Philosophy in Belgrade); (B) photo of the section segment with a visible cut for building 27 and the natural on the left; (C) photo of the section segment with a visible cut for building 32 and the natural on the right.

**
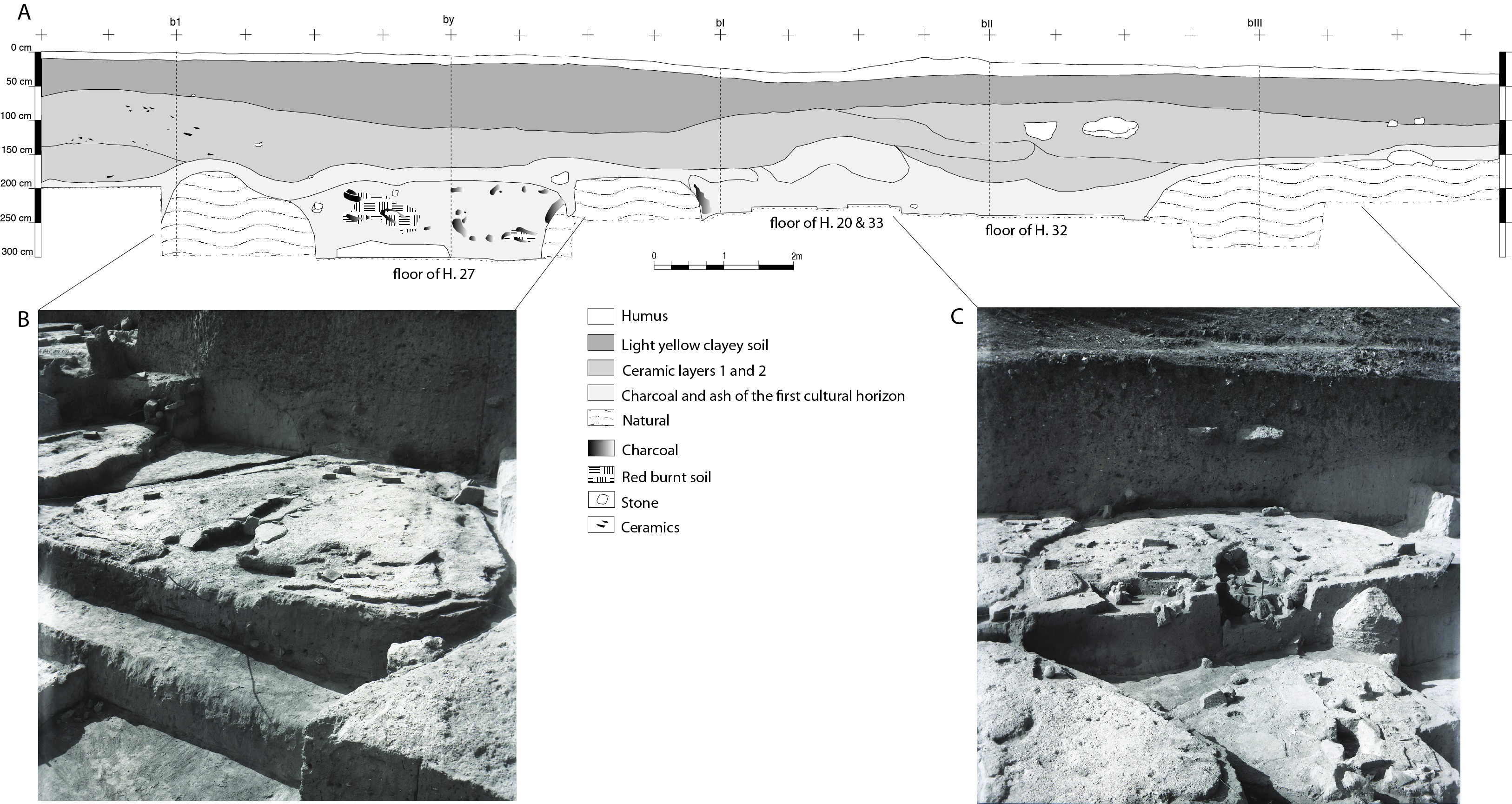
**

**Supplementary text 4: Details of dated contexts and samples**

**Location with building 31:** articulated remains of a brown bear were found underneath the floor of Building 31 and a humerus with cutmarks is dated by OxA-24812 (8410±39 BP). A possibly structurally deposited human mandible (Burial 105) was found together with these bear remains.

**
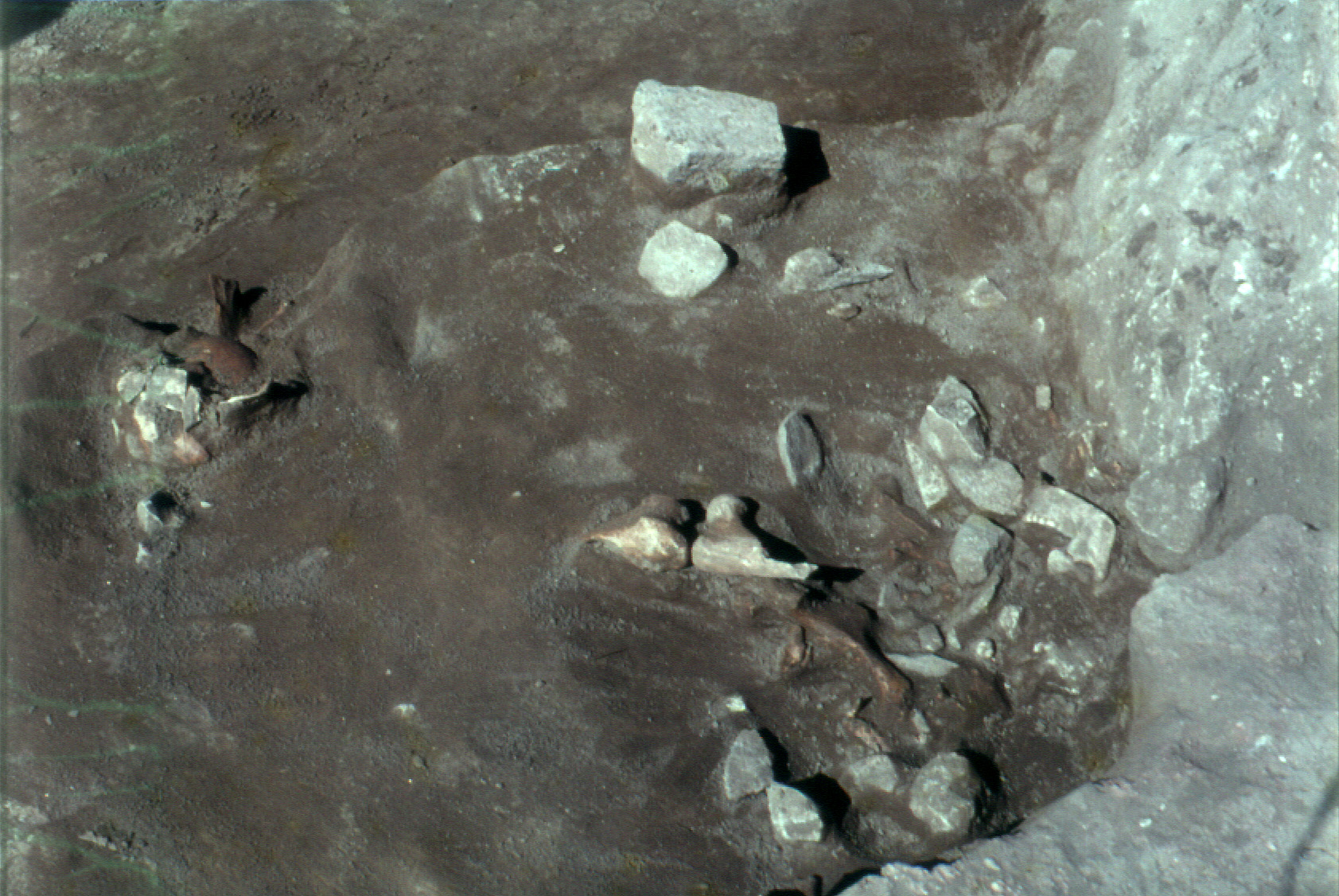
**

**Location with Hearth “a” and burial 22:** (A) Middle Mesolithic occupation zone (dark-stained) around rectangular stone-lined hearth “a” associated with AMS-dated disarticulated human mandible burial 22 (AA-57781: 8374±75 BP – corrected for the reservoir effect), a shed red deer antler fragment (inv. bag: 286: OxA-16074: 8645±40 BP) and (B) a wild boar tusk tool (inv. BB-187: OxA-26549: 8659±45 BP and OxA-26550: 8710±45 BP). The scale is 3 cm.

**A** **B**


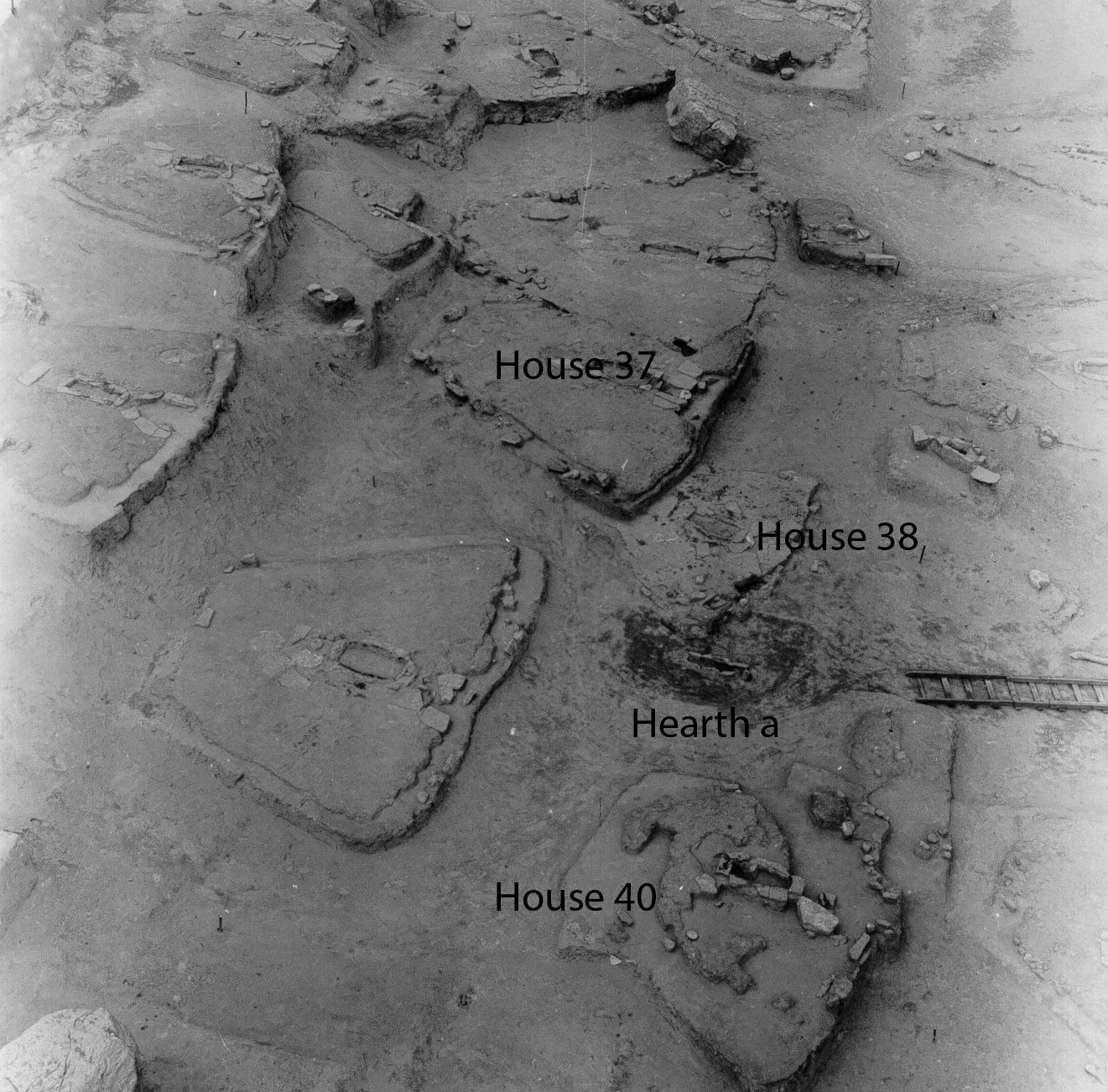

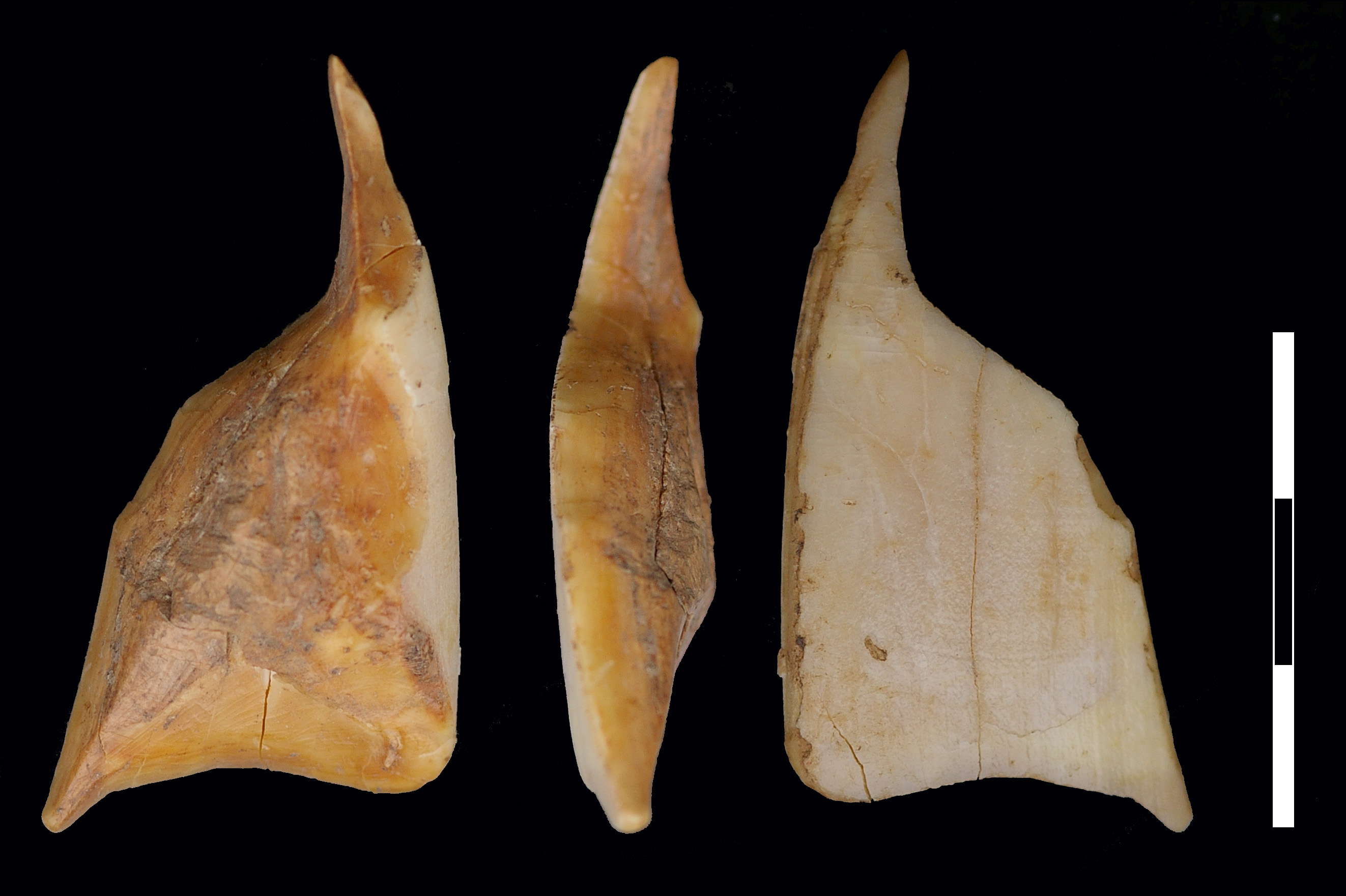


**Location with Buildings 37, and 38:** (A) building 37 with carved centrally placed sandstone querns and (B) AMS-dated bone projectile point found on its limestone floor (Inv. 673: OxA-16082: 7138±37 BP); (C) the floor level and rectangular stone-lined hearth of building 38 that was partly damaged by the construction of building 37; (D) dated red deer antler mattock tool (OxA-26552: 10,035±50 BP) found beneath the floor of this building, which dates older Early Mesolithic occupation deposits. The scale is 3 cm.

A B

**
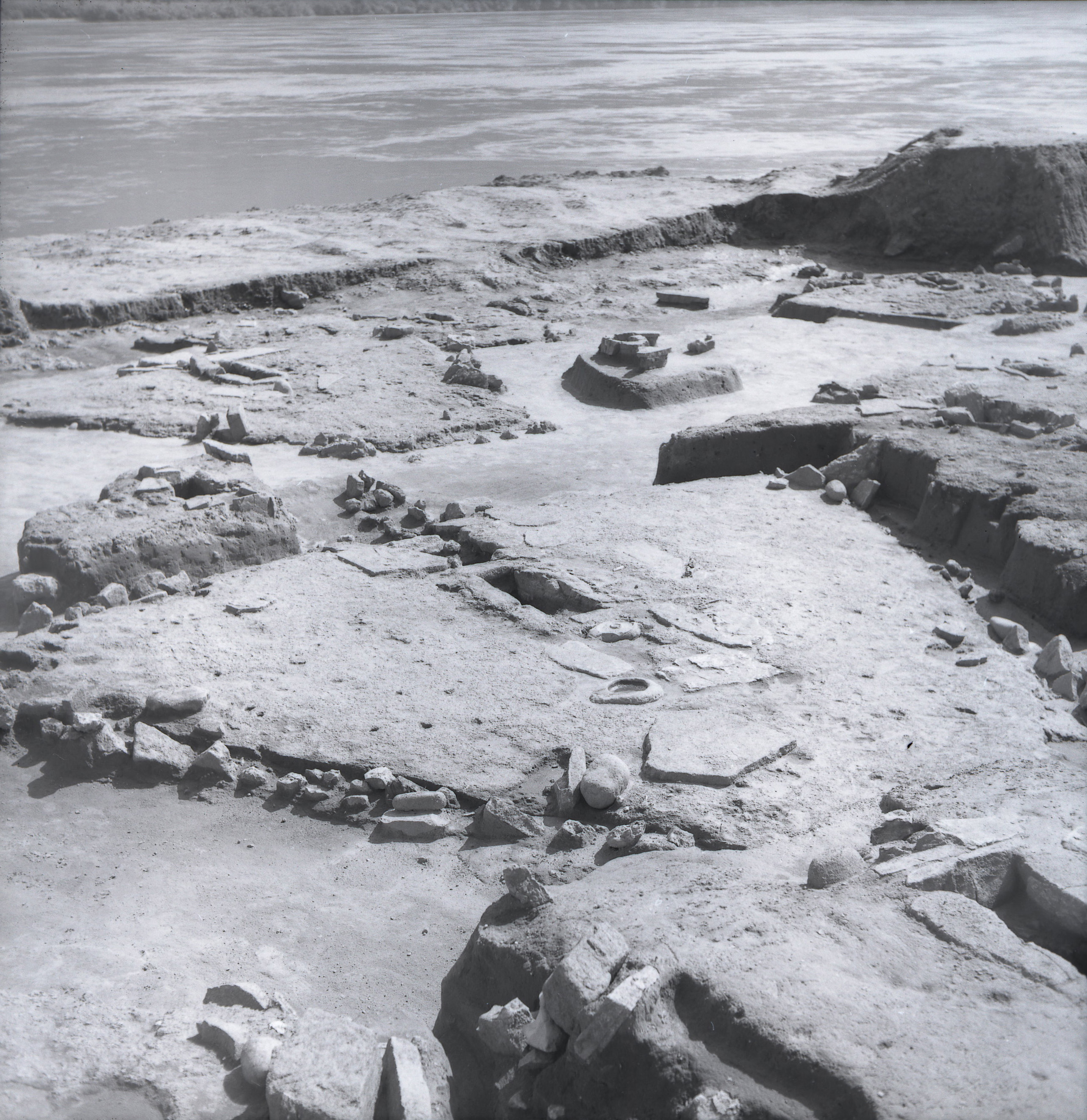

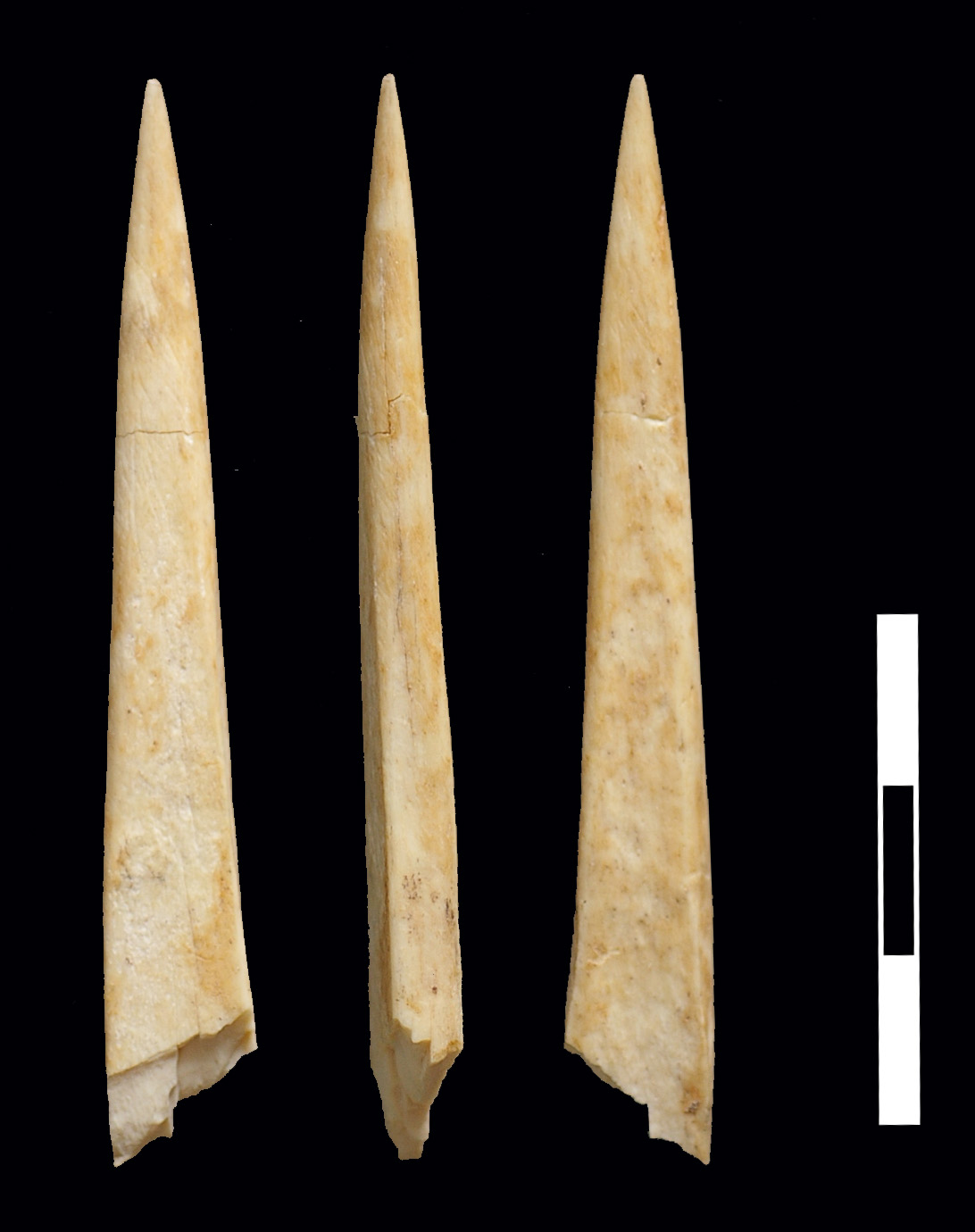
**

C D

**
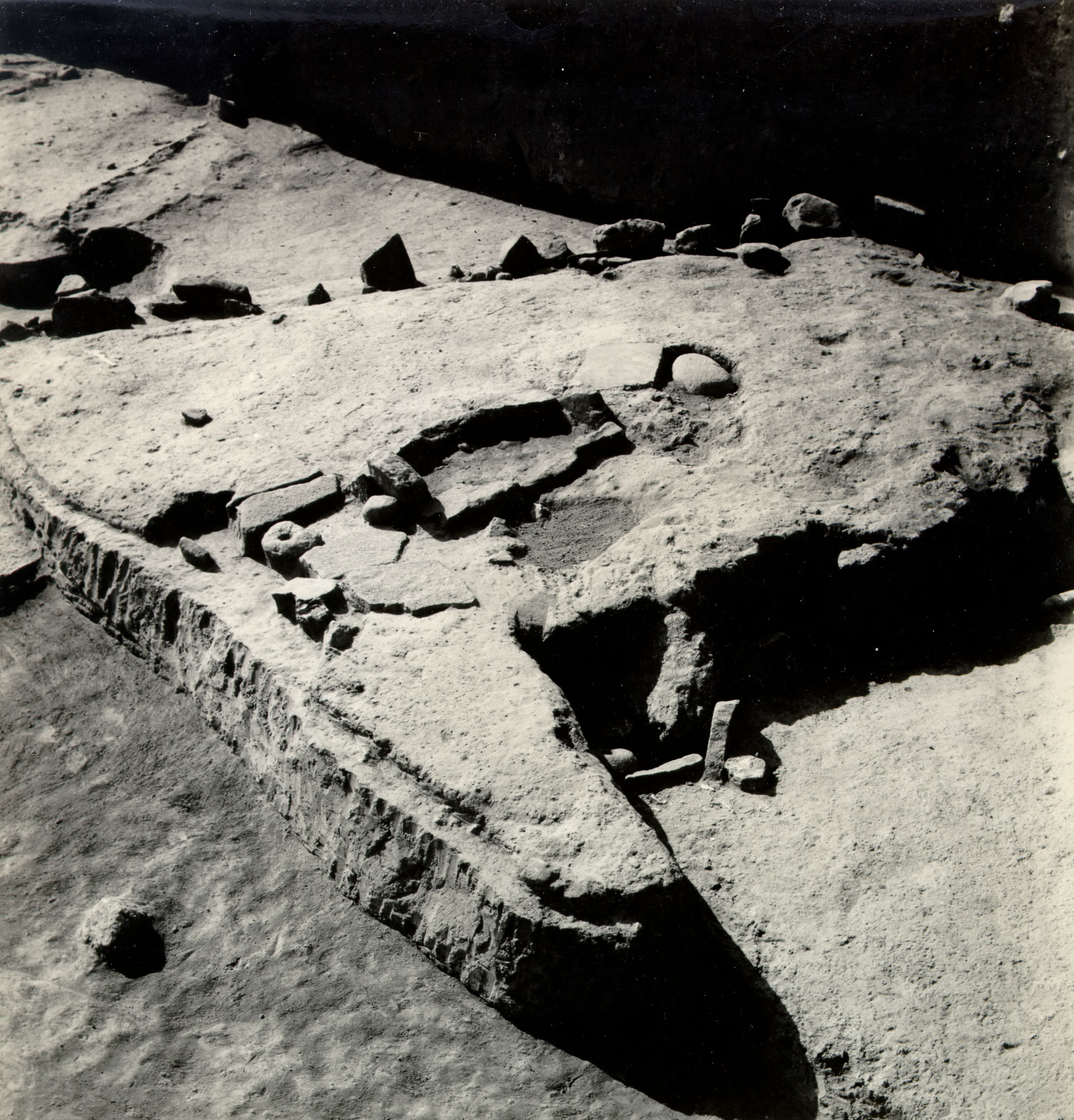

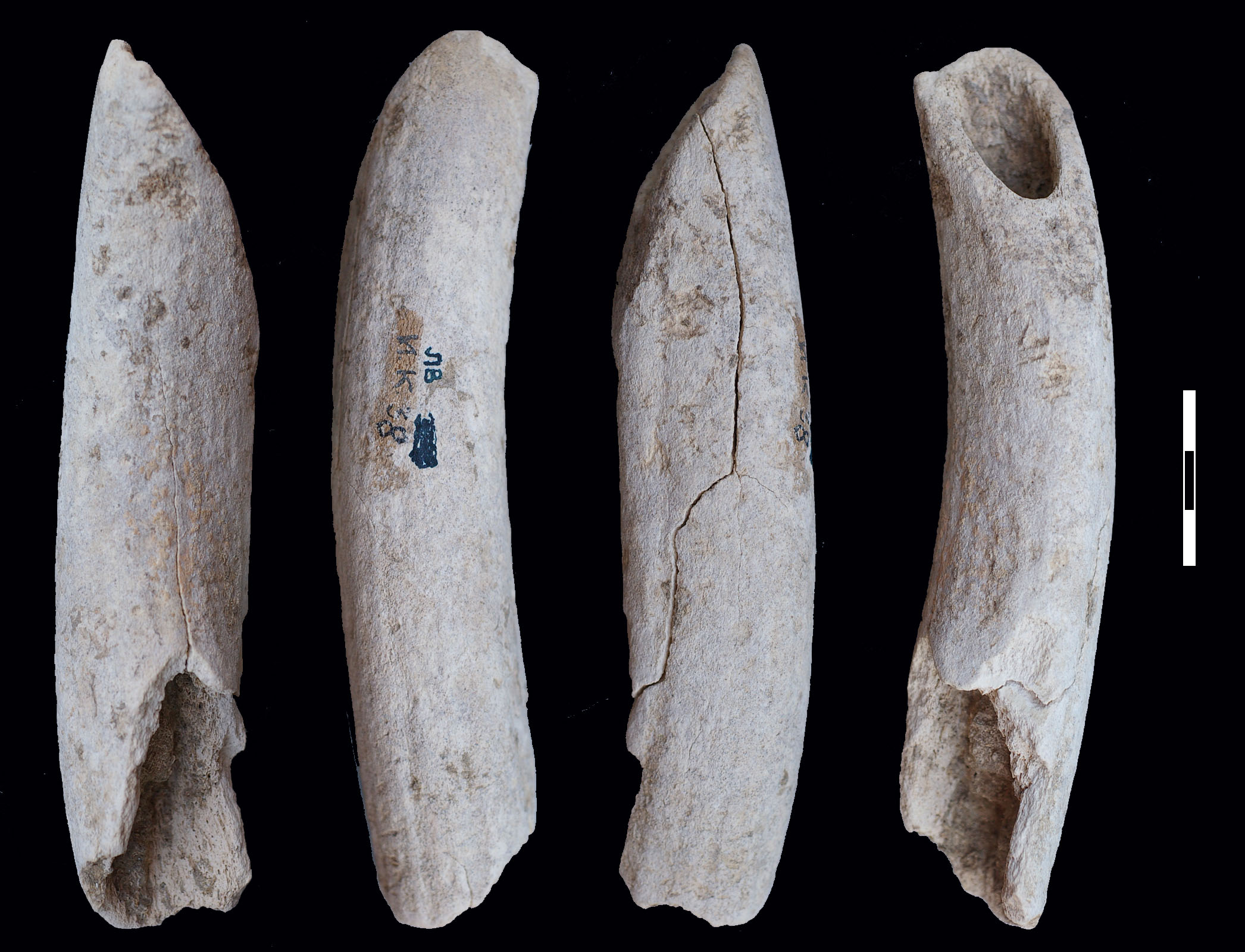
**

**Location with buildings 47, 47’ and burial 122:** (A) The floor of older building 47’, superposed by the floor of younger building 47; An “aniconic” (unornamented) sculpted boulder is surrounded by the slabs of the building’s ash-place. AMS-dated structurally deposited disarticulated human skull burial 122 (OxA-16005: 7098±47 BP and OxA-16006: 7109±41 BP, both corrected for the reservoir effect) is found within the deposit between buildings 47 and 47’. (B) Facing northeast: two neonate burials (123 and 124) are visible in the back area of the building, most likely dug from the level of younger building 47. OxA-16004 (9730±50 BP) dates a sample also found between the floors of building 47 and 47’, but the dated remains are residual and the date corresponds with another Early Mesolithic date from beneath the floor of older building 47’ (OxA-16072: 9850±50 BP), strongly suggesting that older deposits (containing residual remains of animal bones, one of which we dated with OxA-16072) must have been dug here during phase I-II and secondarily used for covering the floor of building 47’ before the floor of younger building 47 was built.

A B

**
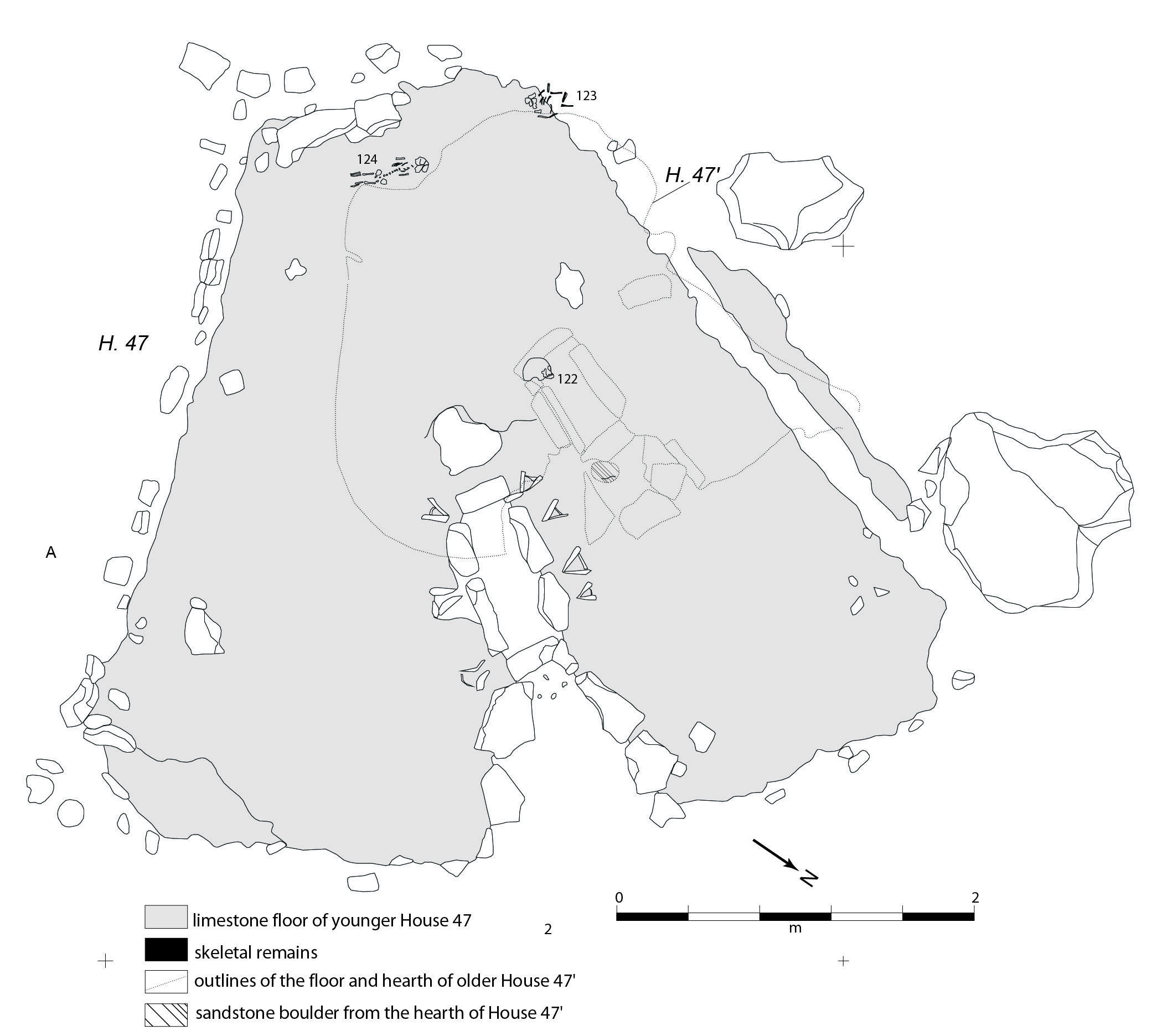

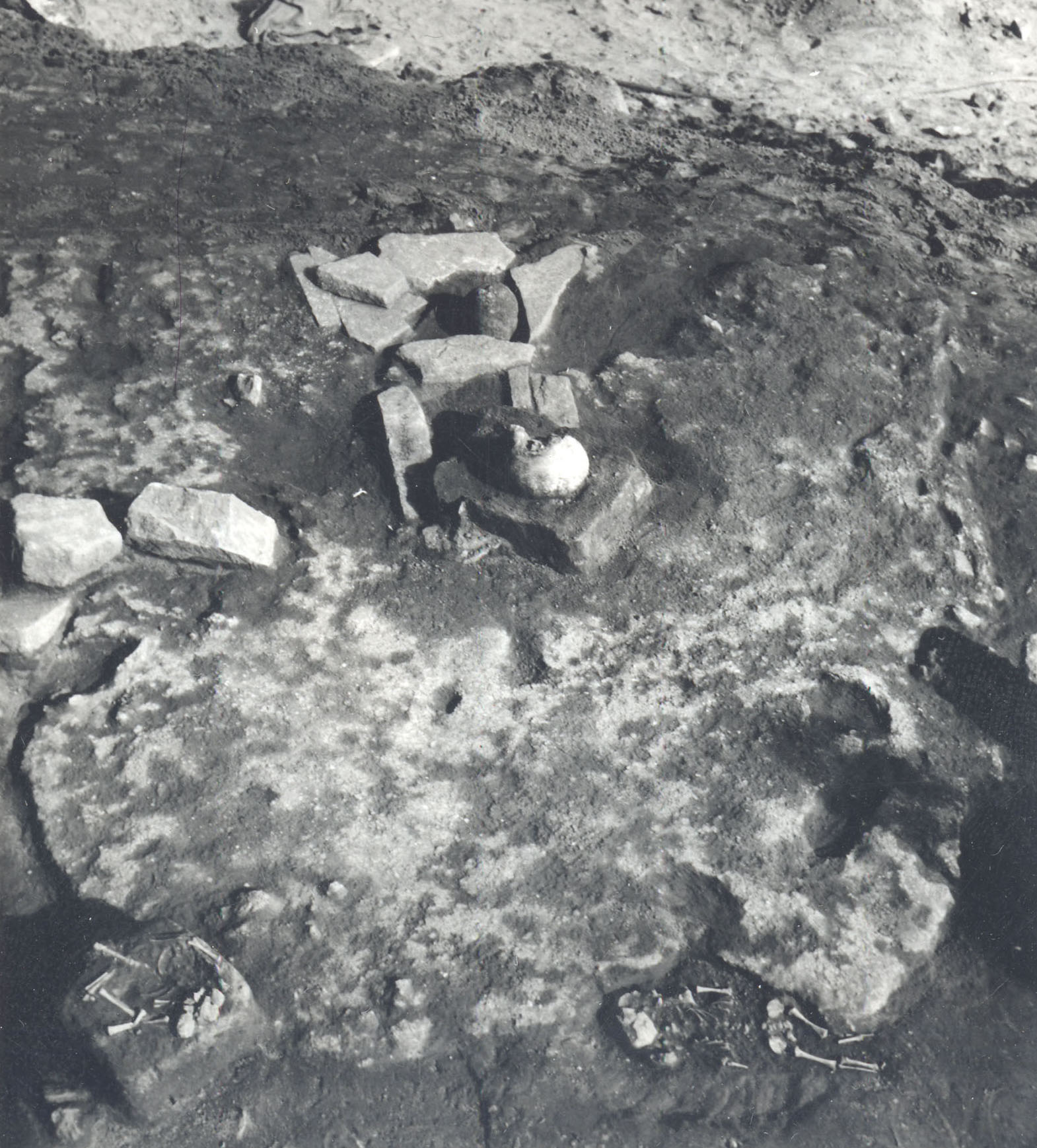
**

**Location with buildings 34 and burial 26:** (A) AMS-dated burial 26 (AA-57782: 7122±57 BP and OxA-25206: 7058±37 BP, both corrected for the reservoir effect) dug through the floor of building 34, behind the stone-lined hearth, and laid to rest as a typical Mesolithic extended supine burial and can confidently be assigned to phase I-II; (B) A red deer mandible found on the floor of the building was dated by OxA-16009 (7165±40 BP), giving a comparable date too for the end of the occupation of building 34.

**A** **B**


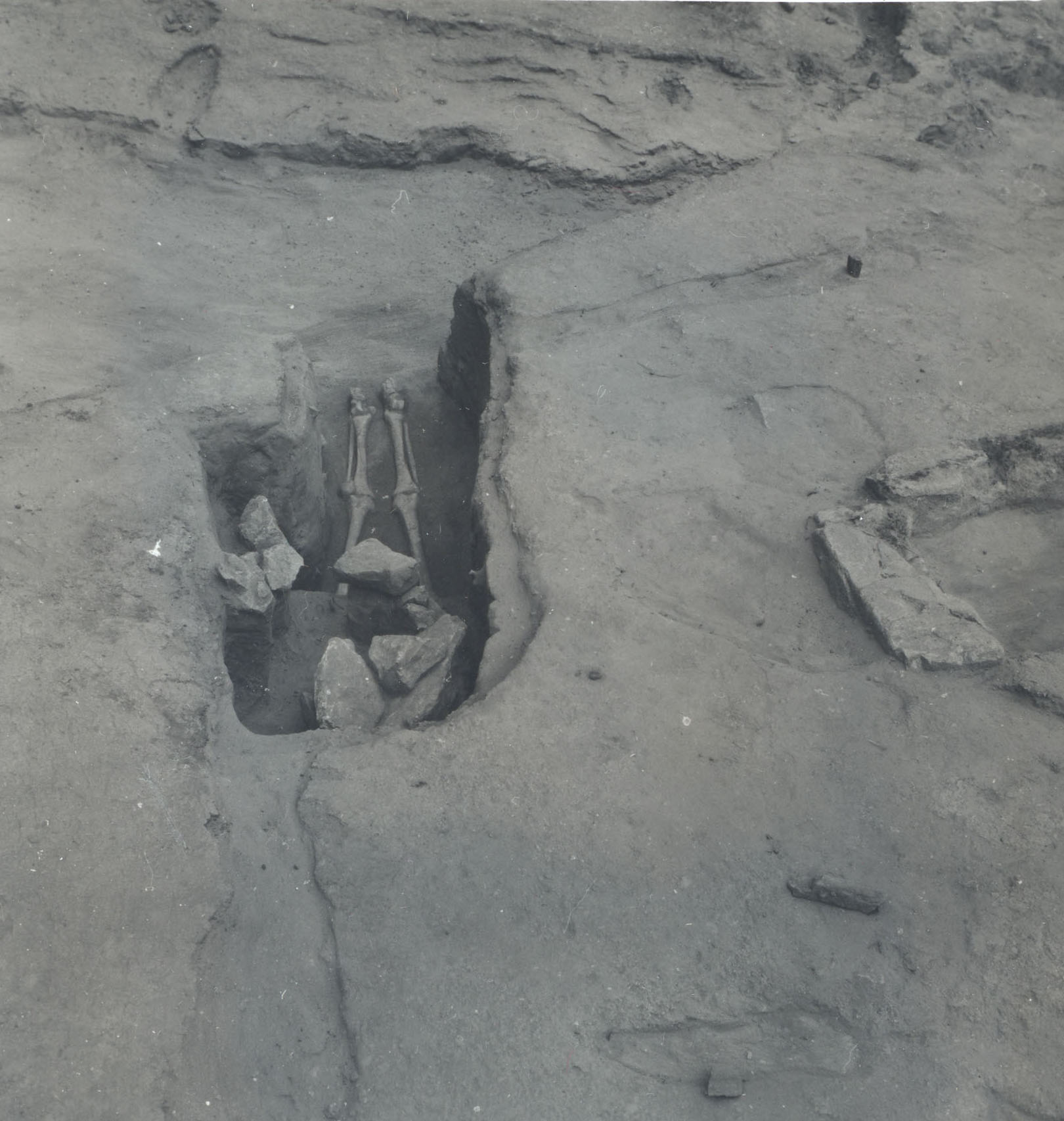

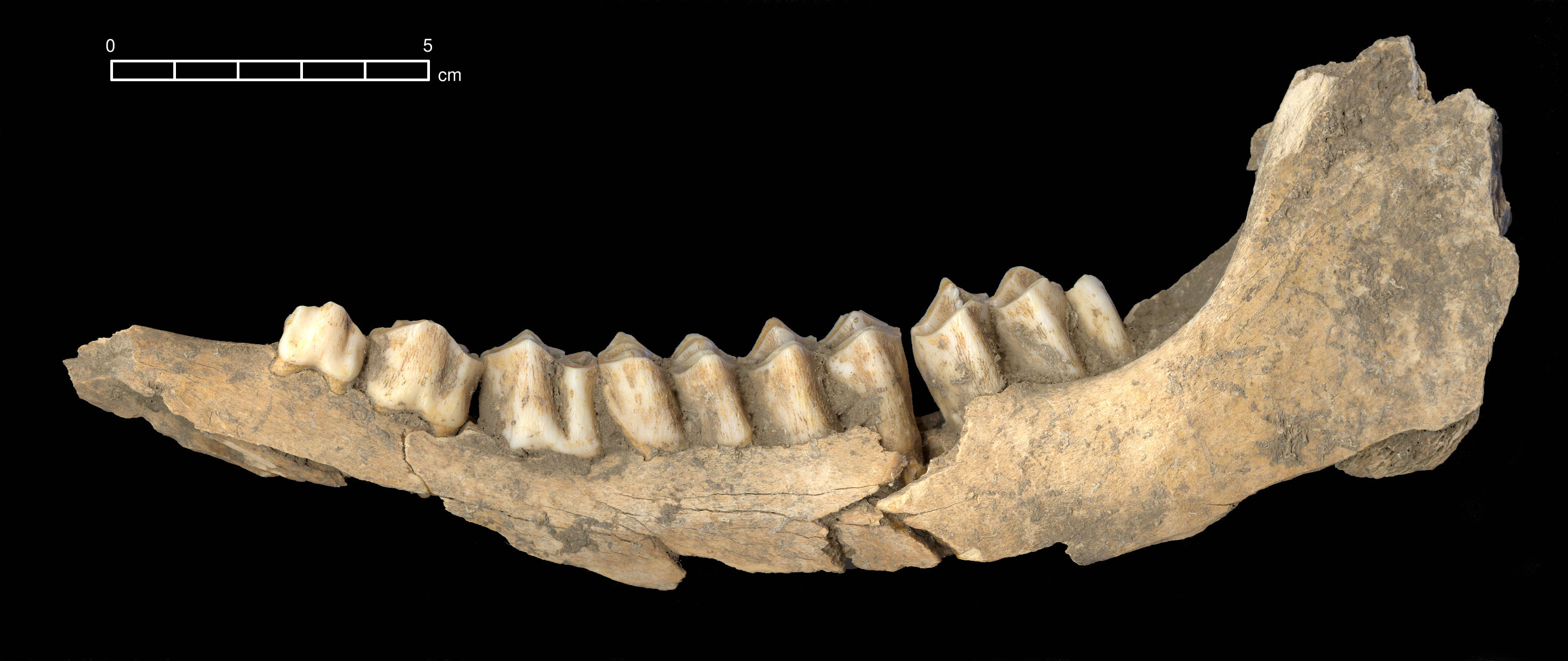


**Location with Buildings 24, and Burials 8 and 9:** (A) AMS-dated burial 8 (AA-58319: 6690±54 BP and OxA-25207: 6984±39 BP both corrected for the reservoir effect) found on the layer of fill covering the floor of building 24 (phase I-II) as crouched inhumation typical of Neolithic burial rites and confidently assigned to phase III. Burial 9 is dated by OxA-25208: 6893±50 BP, after the correction for the reservoir effect; (B) the floor of building 24 and a concentration of neonate burials in its back zone. Neonate burial 94 is AMS-dated by OxA-16010: 6980±81 BP, after the correction for the reservoir effect. An AMS-dated red deer animal tooth (OxA-X-2176-18: 7285±45 BP) found in the zone of the hearth of building 24 likely dates the construction/occupation of this building.

A B

**
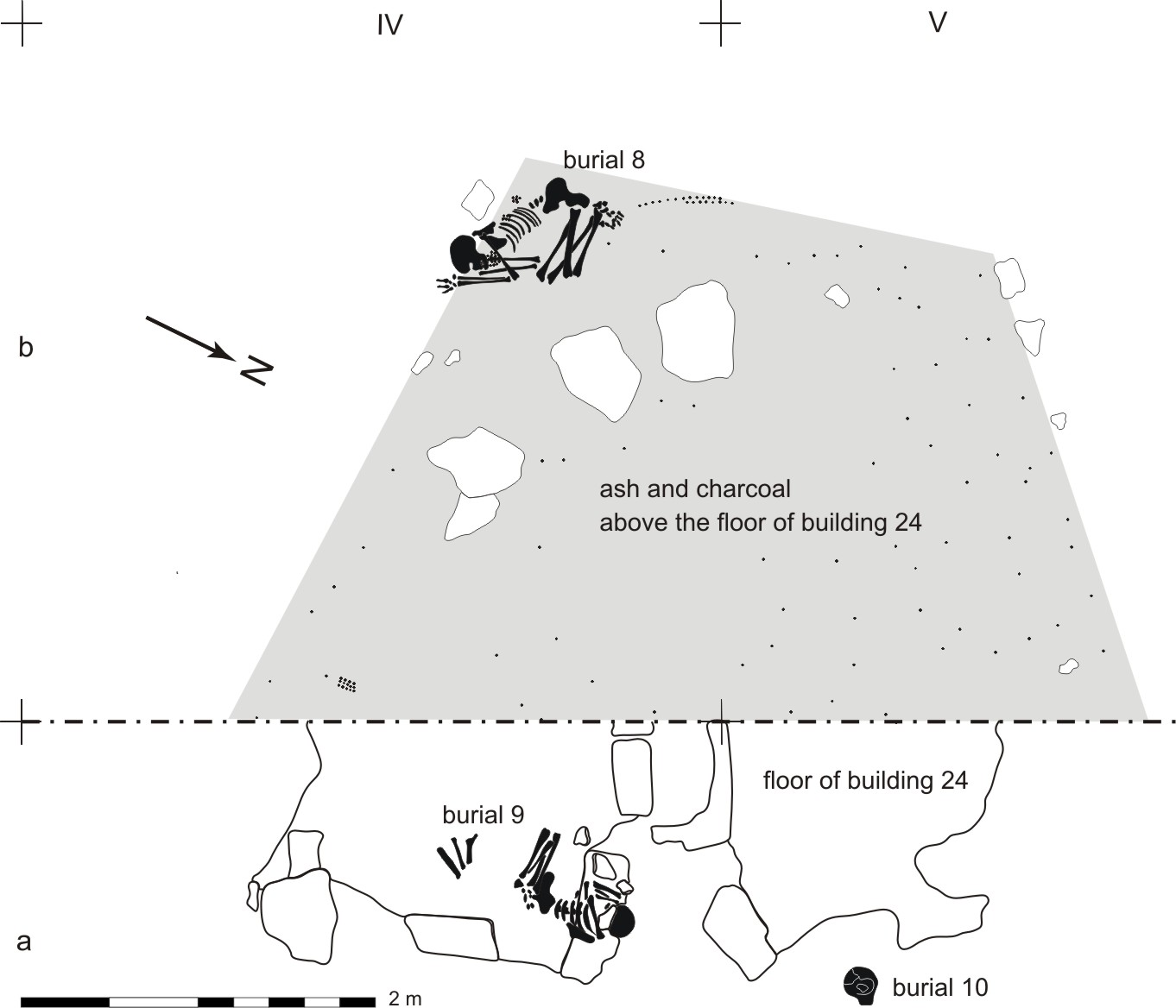

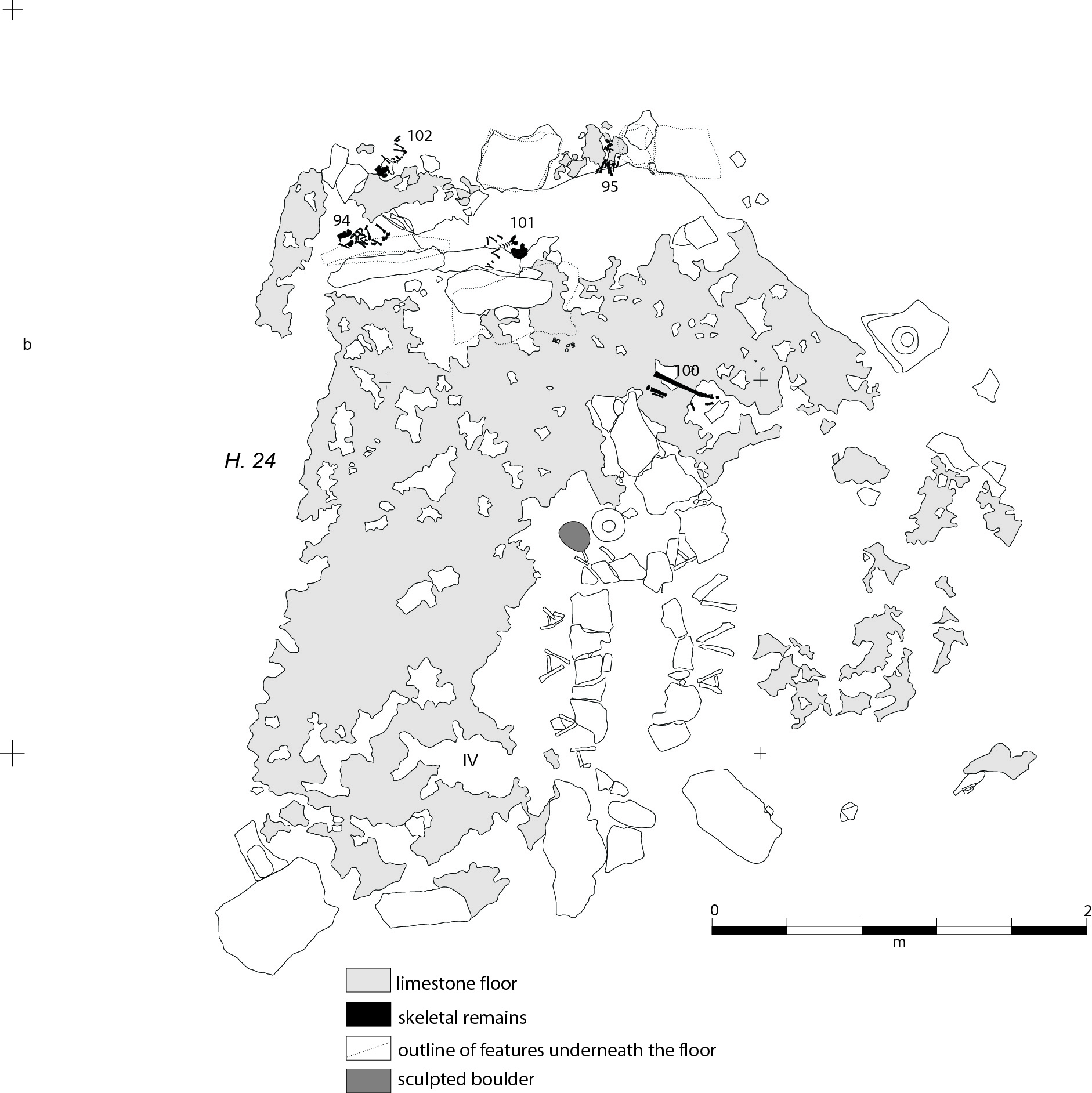
**

**Location with buildings 65/XXXV and burials 54c, 54d and 54e:** (A, B) Both images show the position of AMS-dated burials. Burial 54d (AA-57783: 7065±55 BP and OxA-25213: 7269±68 BP, both after the correction for the reservoir effect); burial 54c is dated by OxA-25209: 7169±52 BP after the correction for the reservoir effect; burial 54e is dated OxA-25210: 7155±54 BP after the correction for the reservoir effect. An animal bone sample from the back area of building 65/XXXV is dated by OxA-X-2176-19 (7314±40 BP).

**
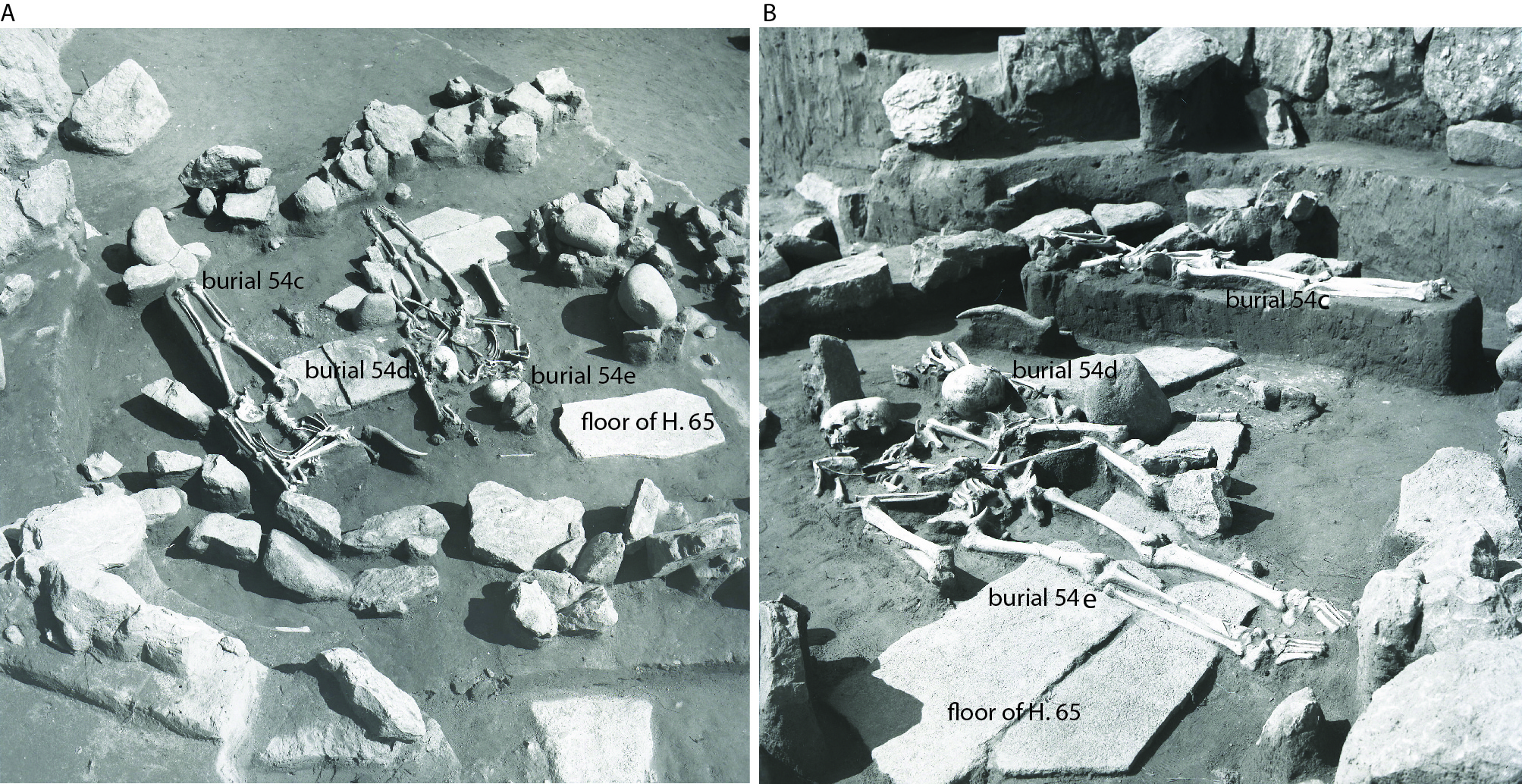
**

**Location with buildings 21, and 22 and burials 7/I and 7/II:** (A, B) In a series of overlapped buildings with aligned stone-lined rectangular hearths, structured deposition of two red deer skulls and skeletal remains of two individuals buried in burial 7, which cuts the floor of the youngest building in this location, were directly AMS-dated. OxA-16075 (7157±39 BP) dates structured deposition of red deer antlers found on the floor of older building 22. Articulated supine inhumation burial 7/I is dated by AA-57779: 7157±79 BP and OxA-25204-5: 7224±72 BP, both after the correction for the reservoir effect. Disarticulated skull burial 7/II is dated by AA-57780 (7031±95 BP after the correction for the reservoir effect). A red deer skull found next to the articulated burial has been dated by OxA-16075 (6924±37 BP) and is considered too young for this context. An aurochs skull found in the same context is dated by OxA-32933 (7133±37 BP).

A B


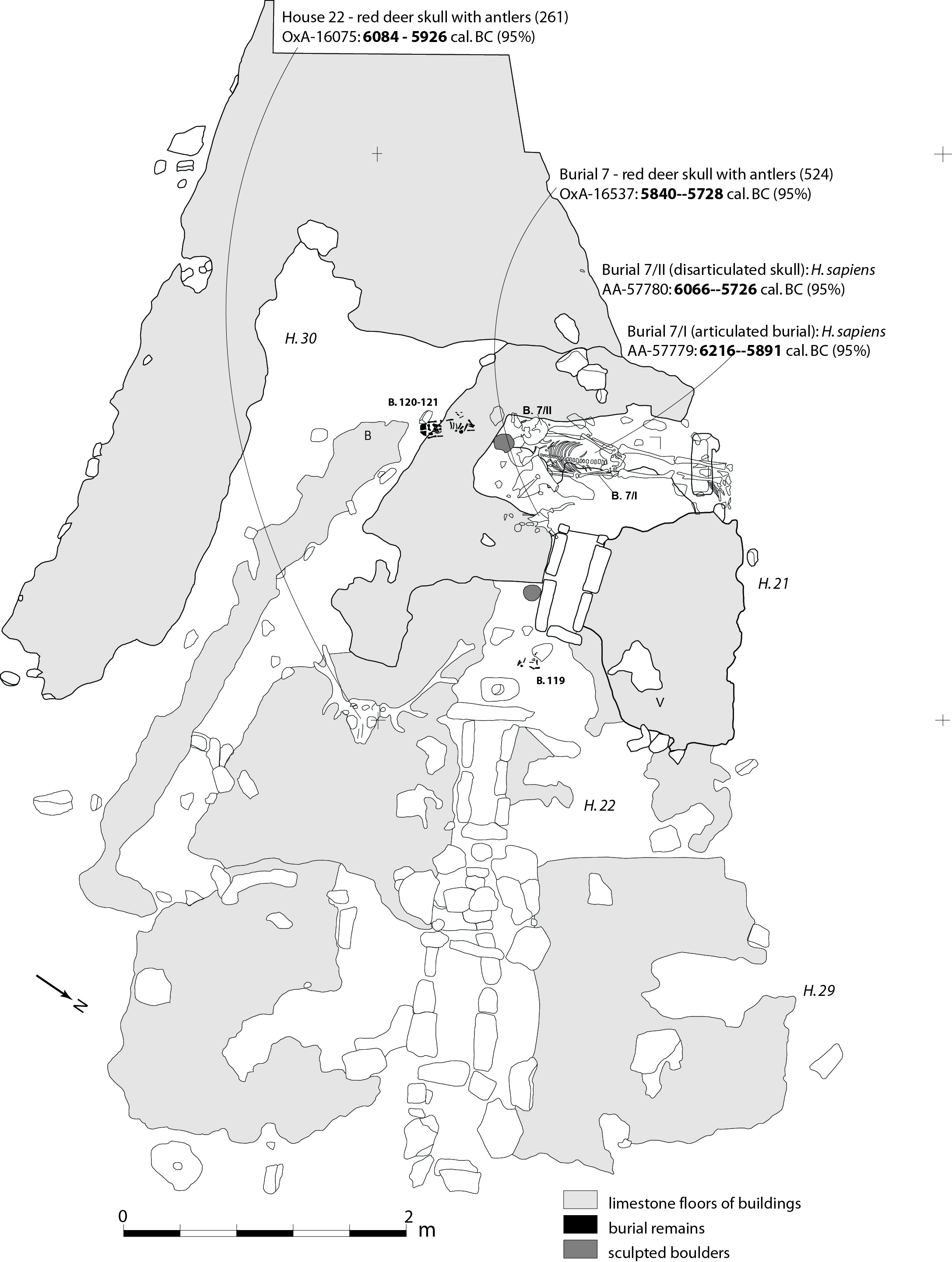

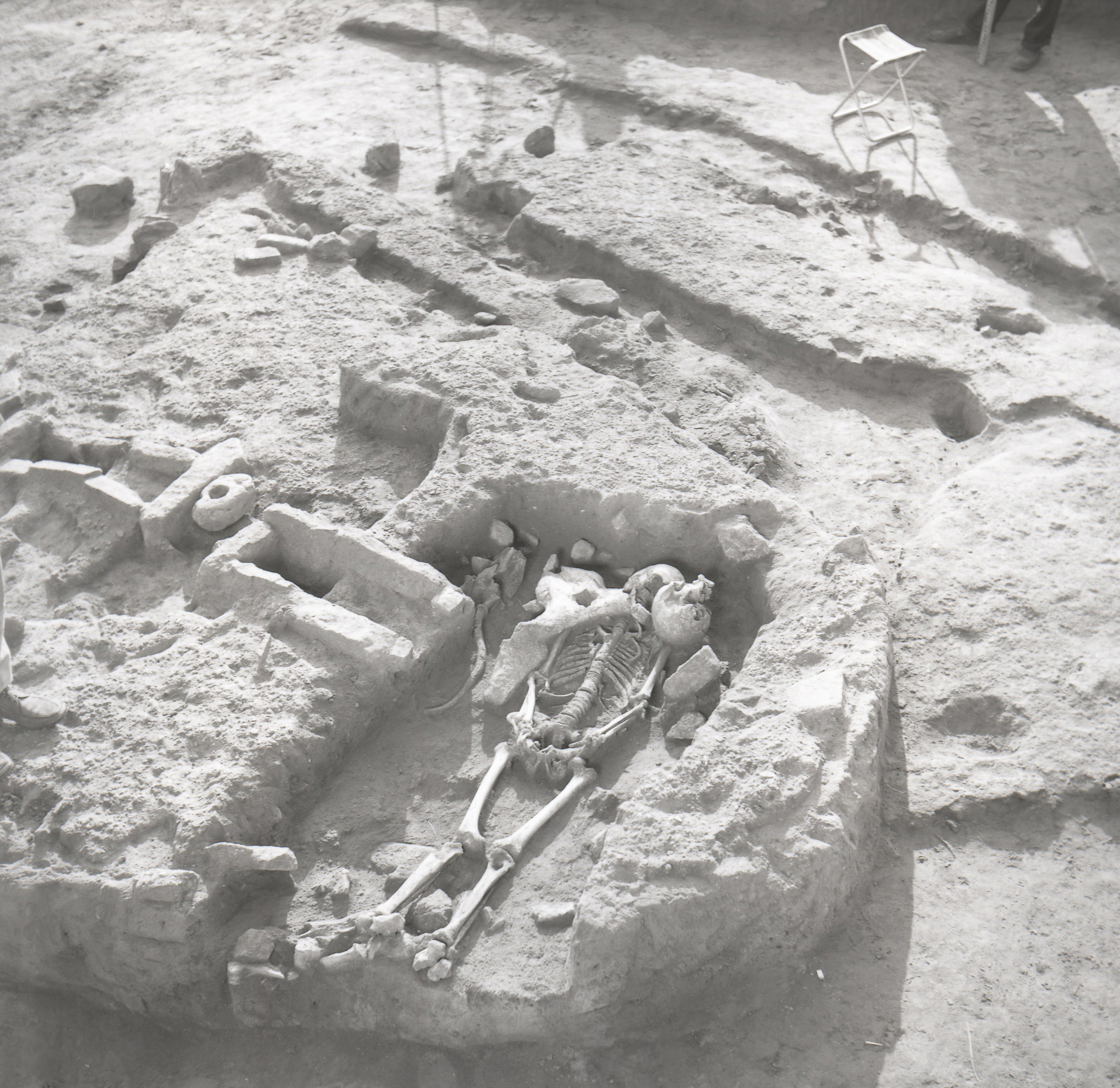


**Supplementary Figure S7.** Osseous tools AMS-dated to Middle Mesolithic phase Proto-Lepenski Vir 2 (see **Supplementary Fig. S3**). (A) OxA-24771 (8871±38 BP) dates red deer antler punch found beneath the floor of building 19; (B) OxA-26551 (8910±45 BP) dates red deer antler intermediate piece (inv. BB-23) found in quadrant A/11, spit 6; (C) OxA-26548 (8265±45 BP) dates wild boar tusk tool (inv. bb-112) found associated with a stone construction in quadrant b/13; (D) OxA-26553 (8840±45 BP) dates a pointed tool on the proximal end of a red deer metapodial (inv. bb-106) found in quadrant C/XV, spit 19.

| A | B |
| --- | --- |
| 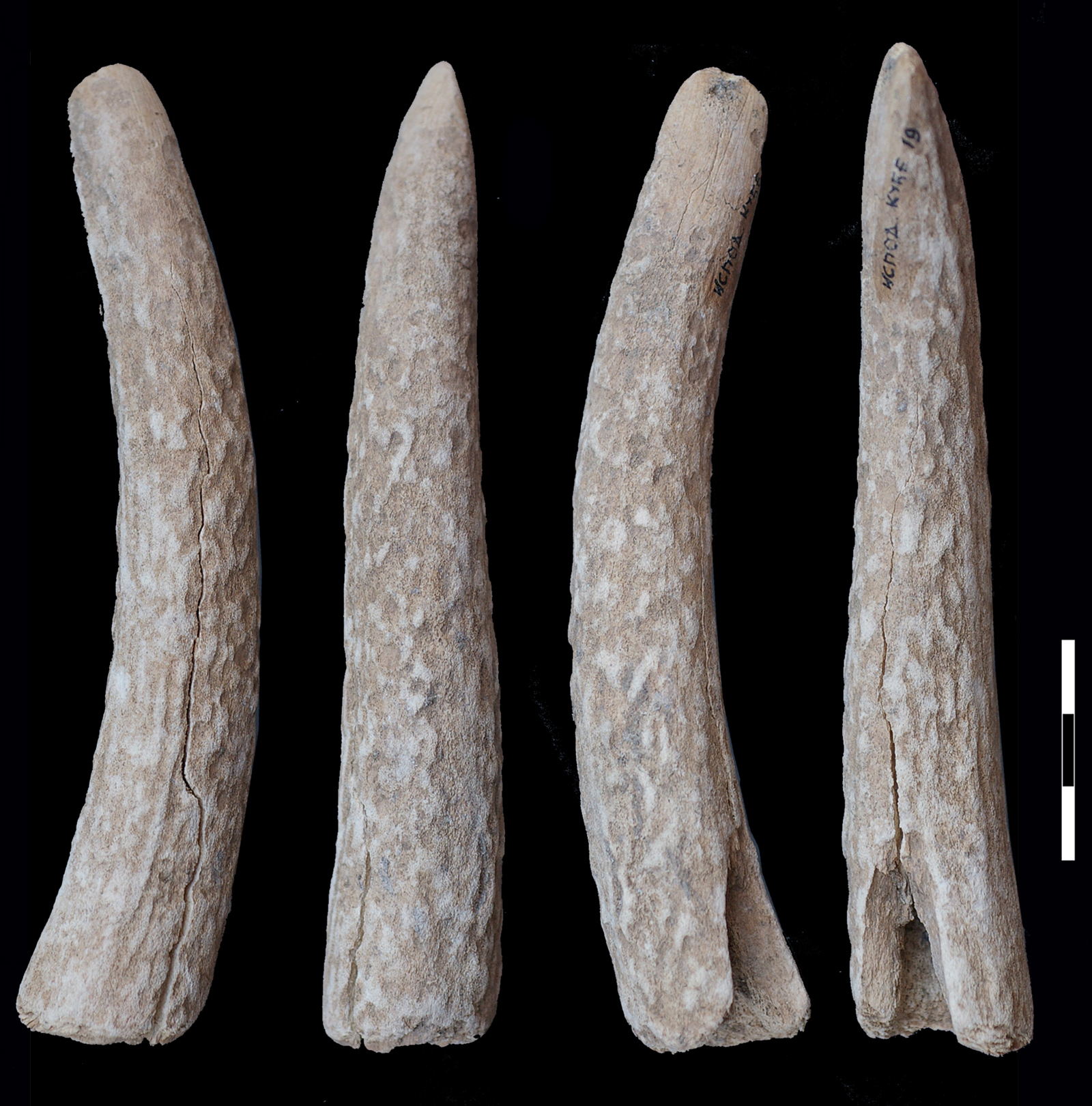 | 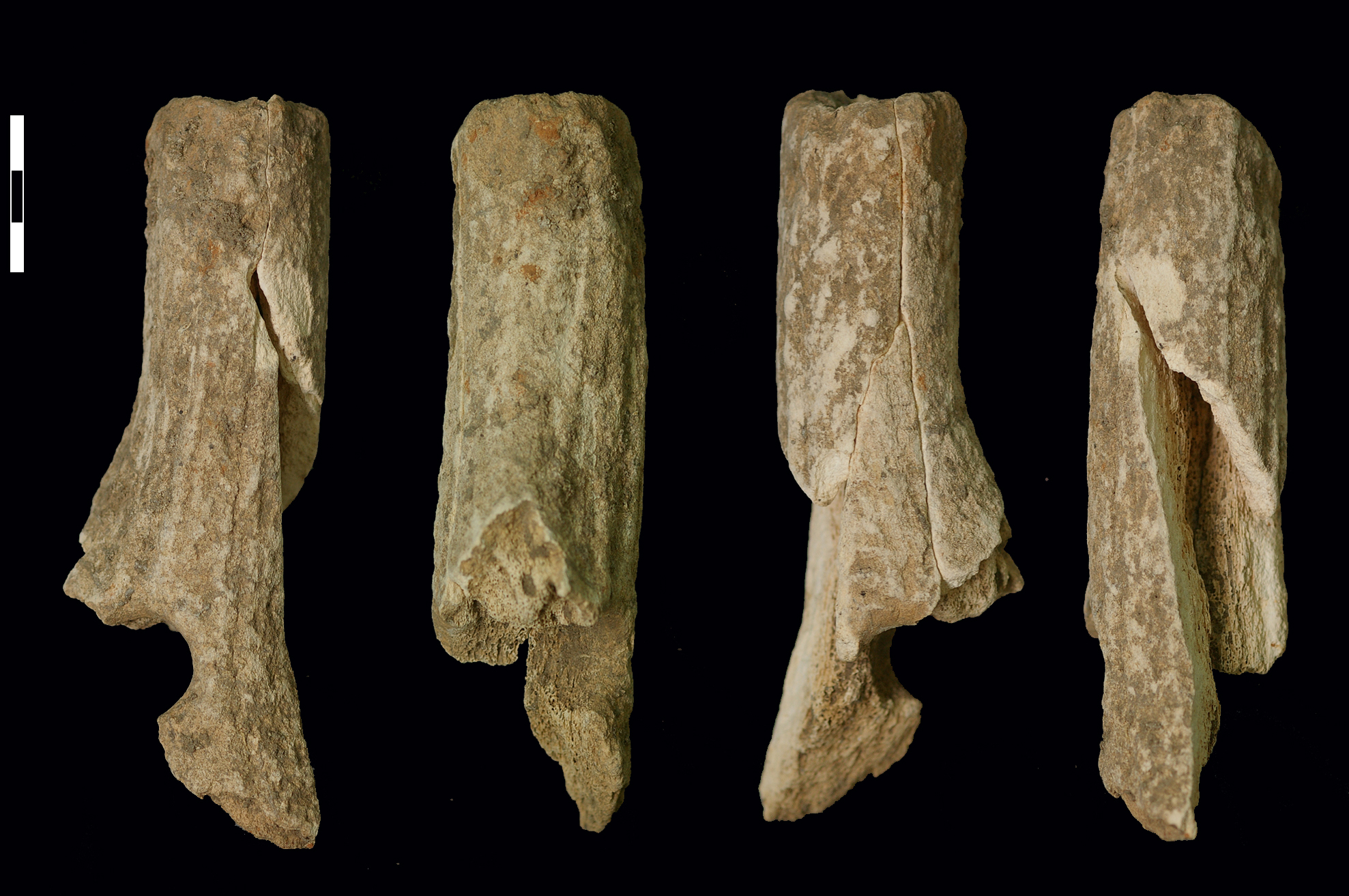 |
| C | D |
| 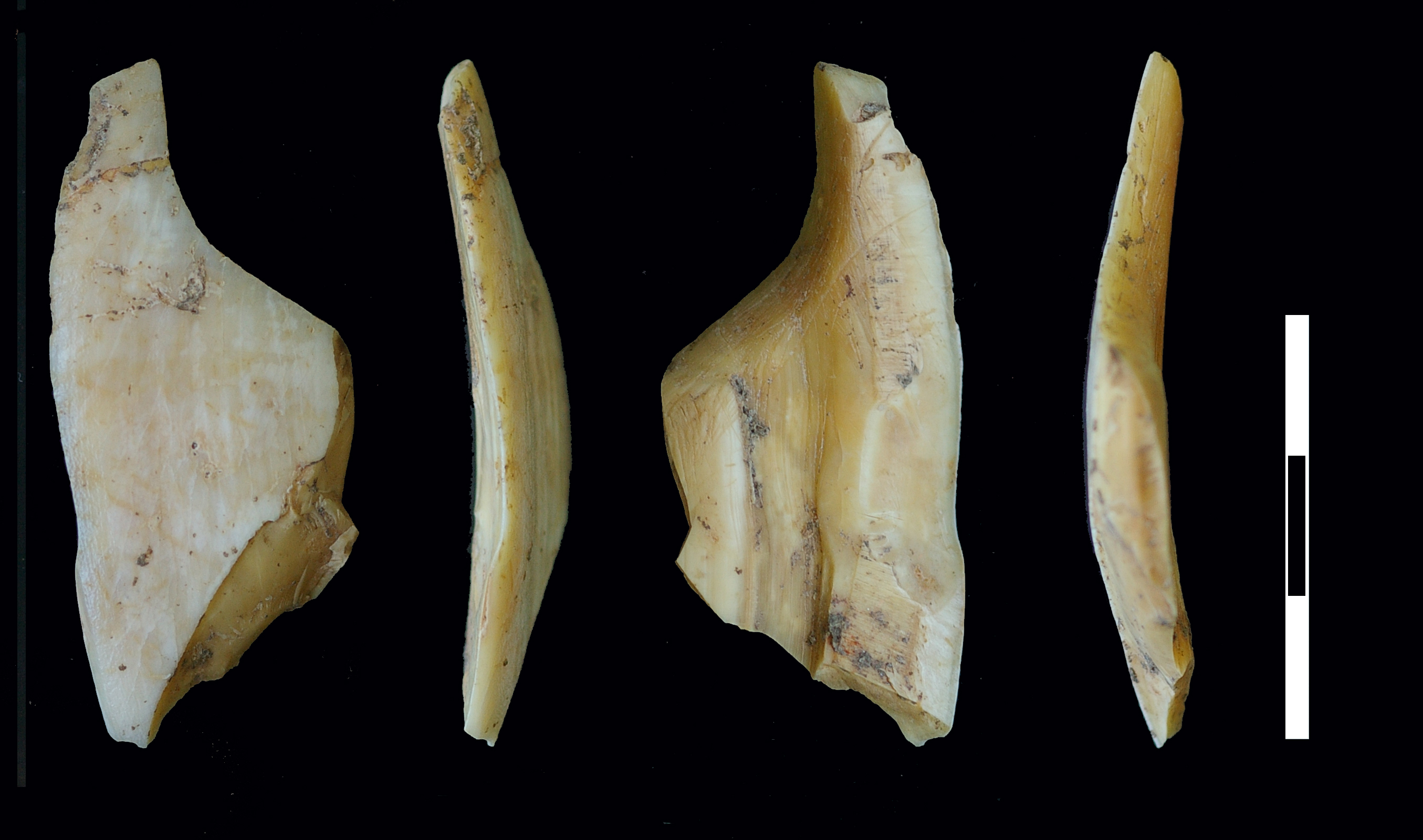 | 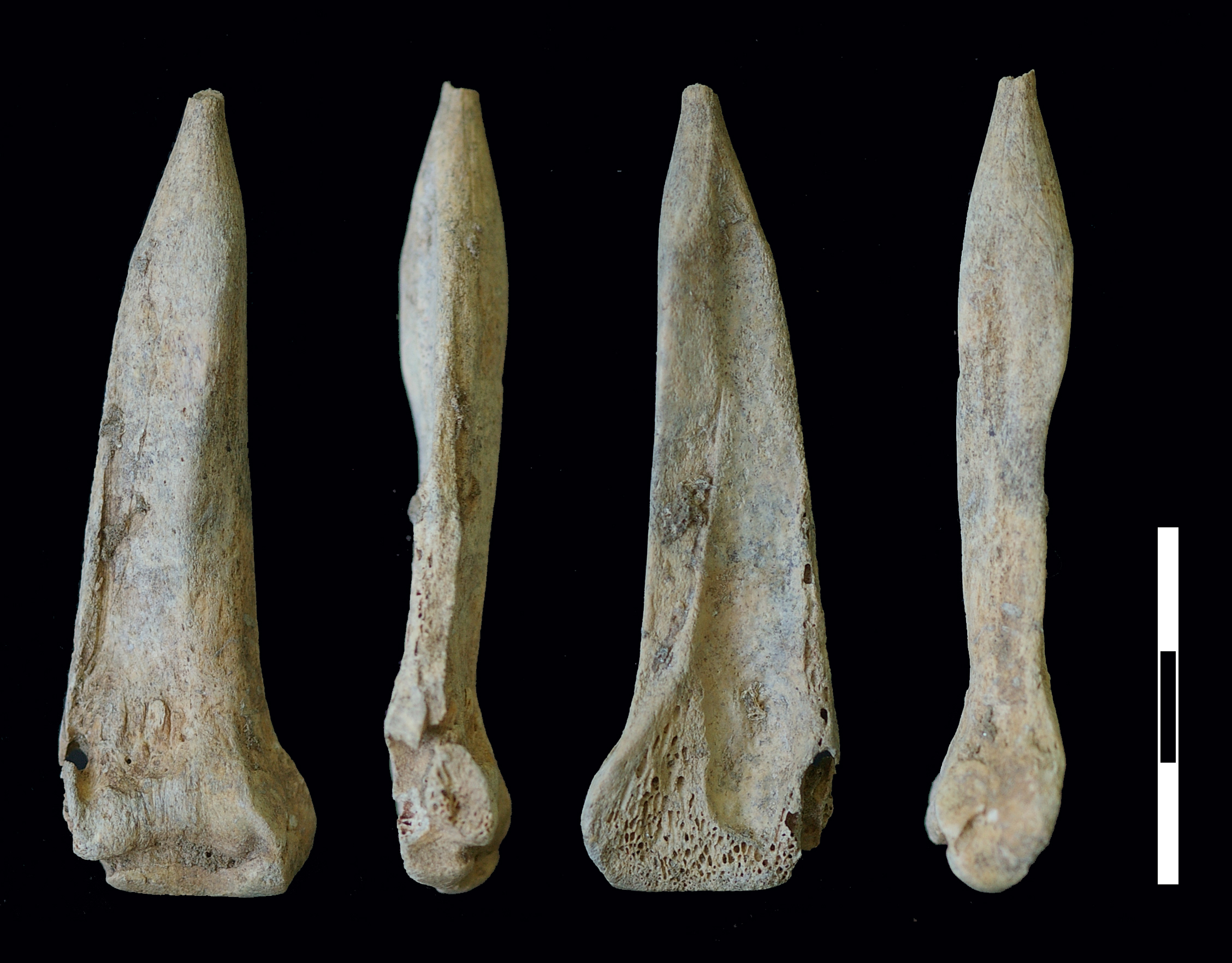 |

**Supplementary Figure 8.** Osseous tools AMS-dated to Mesolithic-Neolithic transitional phase Lepenski Vir I-II (phase with trapezoidal buildings, see **Supplementary Fig. S4**). (A) OxA-16083 (7059±36 BP) dates a pointed tool (projectile, inv. 125) found on the floor of building 5; (B) OxA-16081 (7219±37 BP) dates a pointed tool (projectile, inv. 689) found in the stone construction surrounding building 57/XLIV; (C) OxA-26547 (7396±40 BP) dates a wild boar tusk tool (inv. bb-209) found beneath the floor of building 54; (D) OxA-27901 (7207±35 BP) dates a fishhook (inv. 350) found on the floor of building 4; (E) OxA-16084 (7285±37 BP) dates pointed tool (inv. 349) found on the floor of building 4; (F) OxA-26554 (7462±44 BP after the correction for the reservoir effect) dates a tapered base of a bone tool (inv. bb-193) found beneath the floor of building 4. δ^15^N value of 12.0‰ for this sample indicates that the bone tool comes from a mammal individual likely affected by the reservoir effect due to a heavy consumption of fish resources. ZooMS analysis identified it as belonging to *Canis* sp.

| A | B | C |
| --- | --- | --- |
| 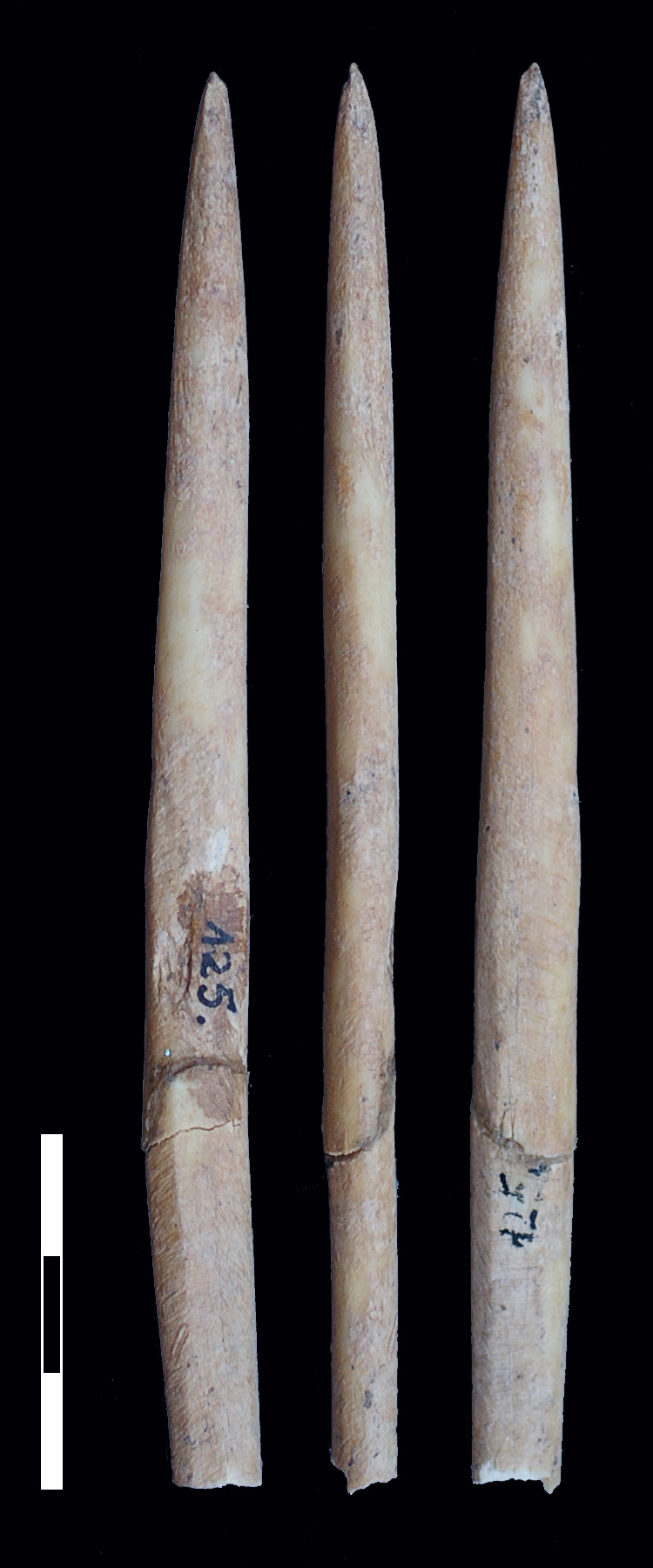 | 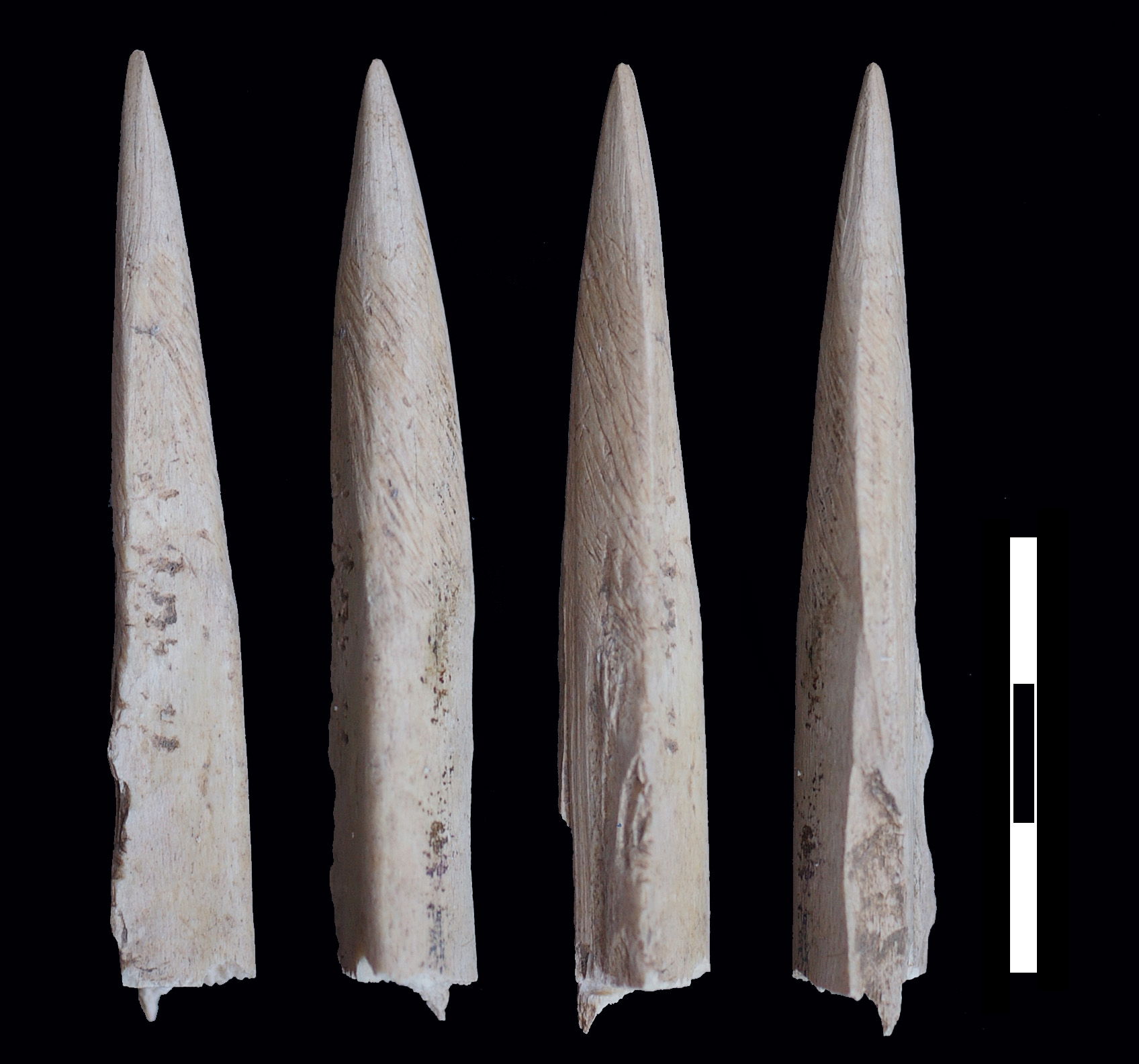 | 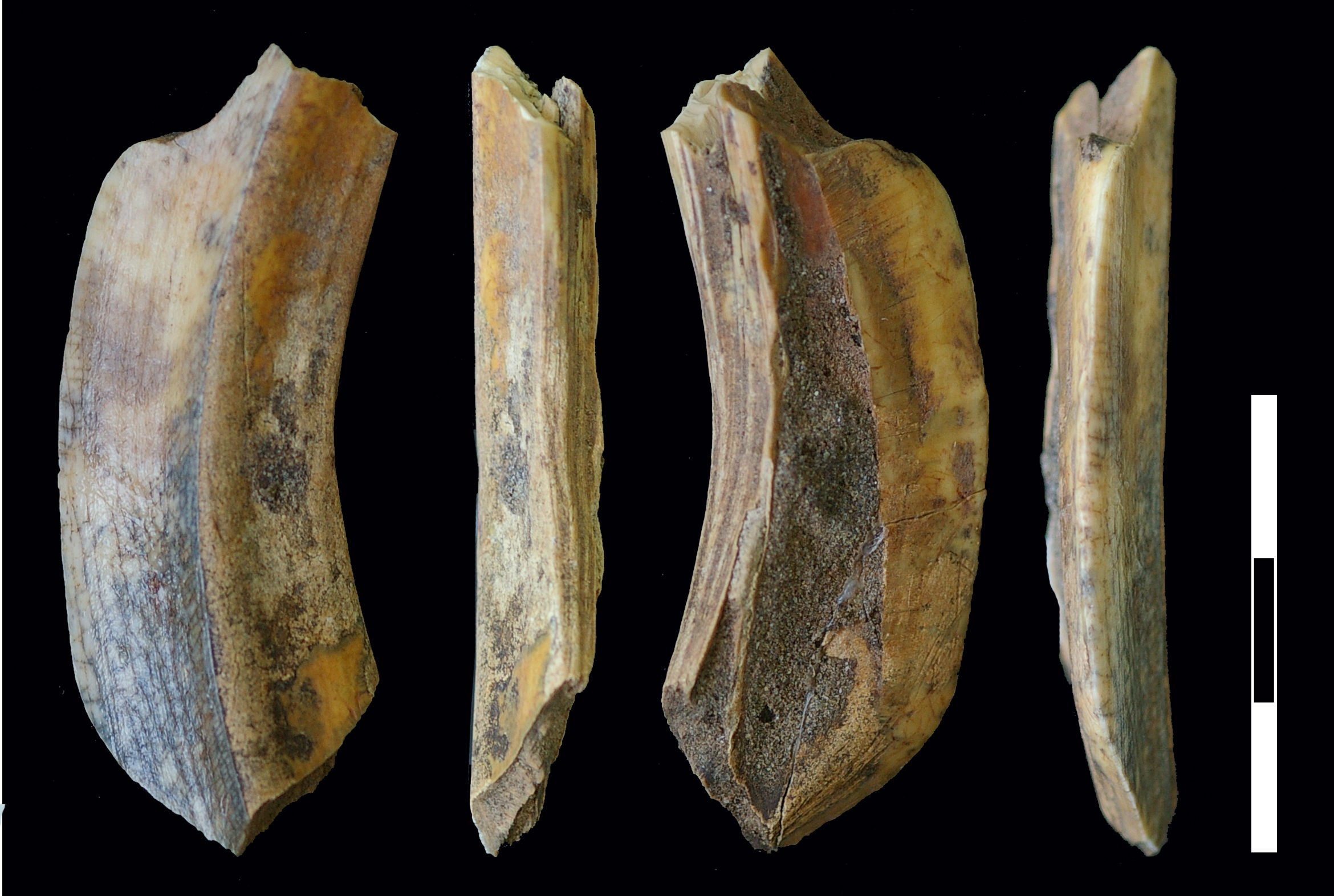 |
| D | E | F |
| 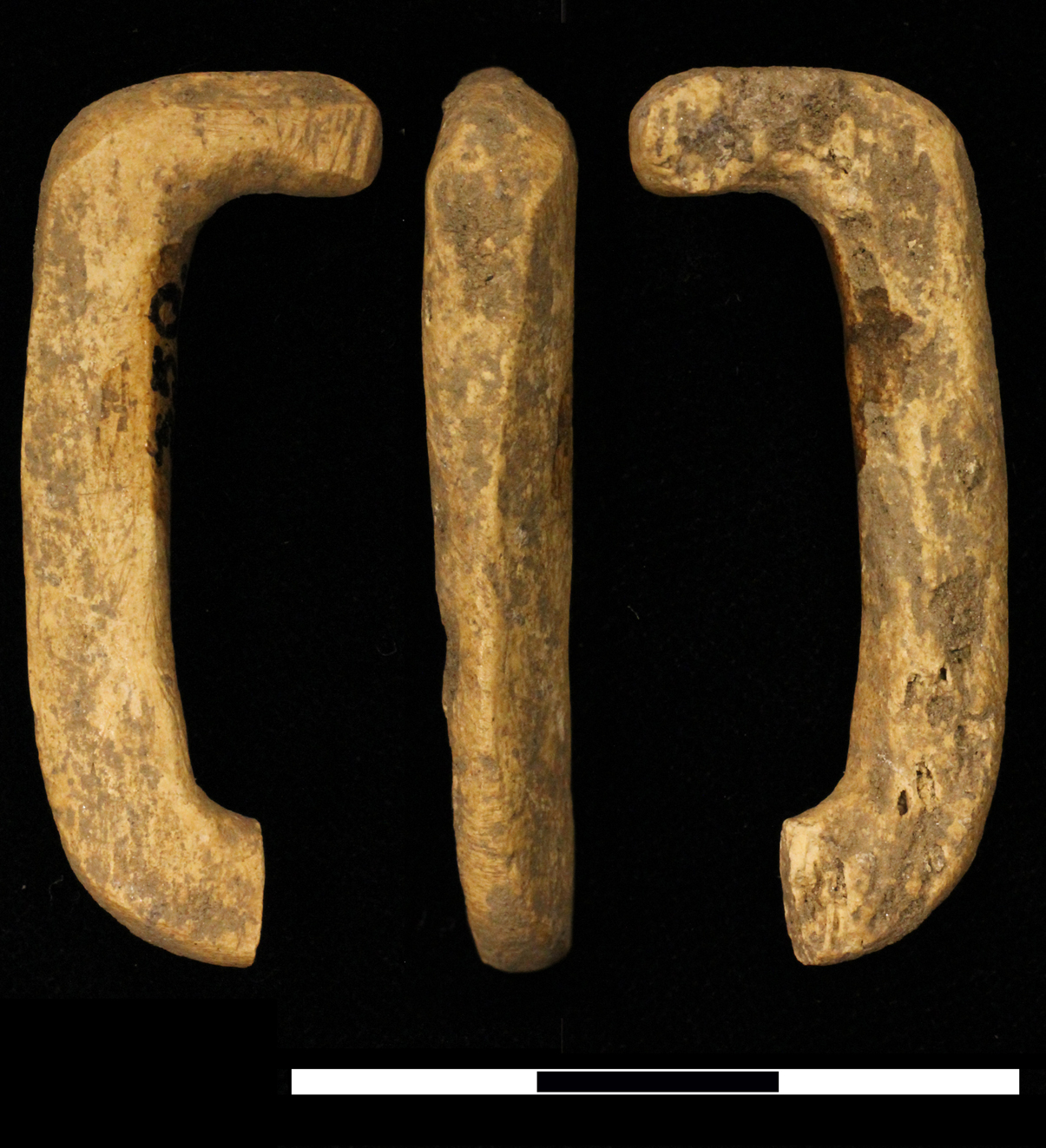 | 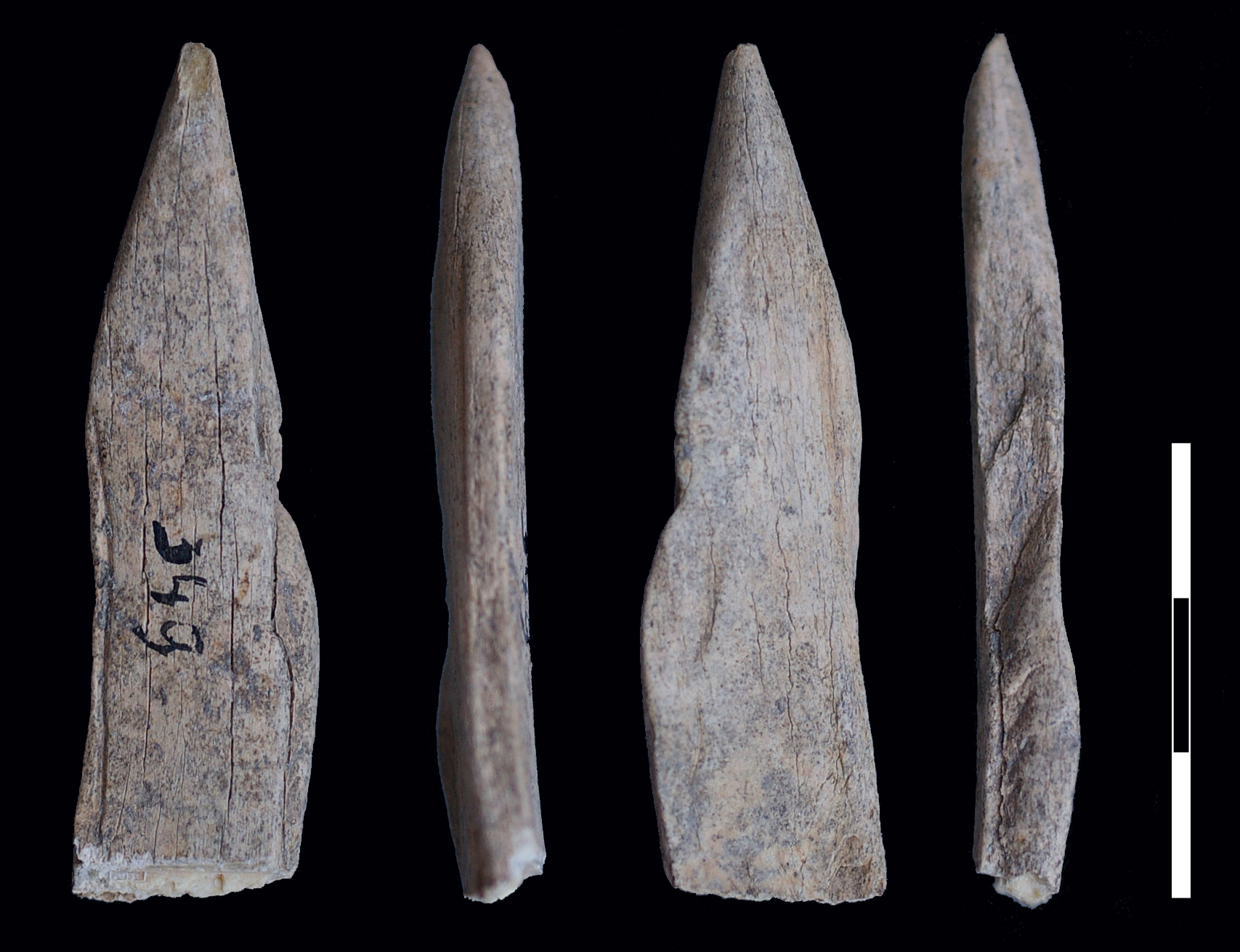 | 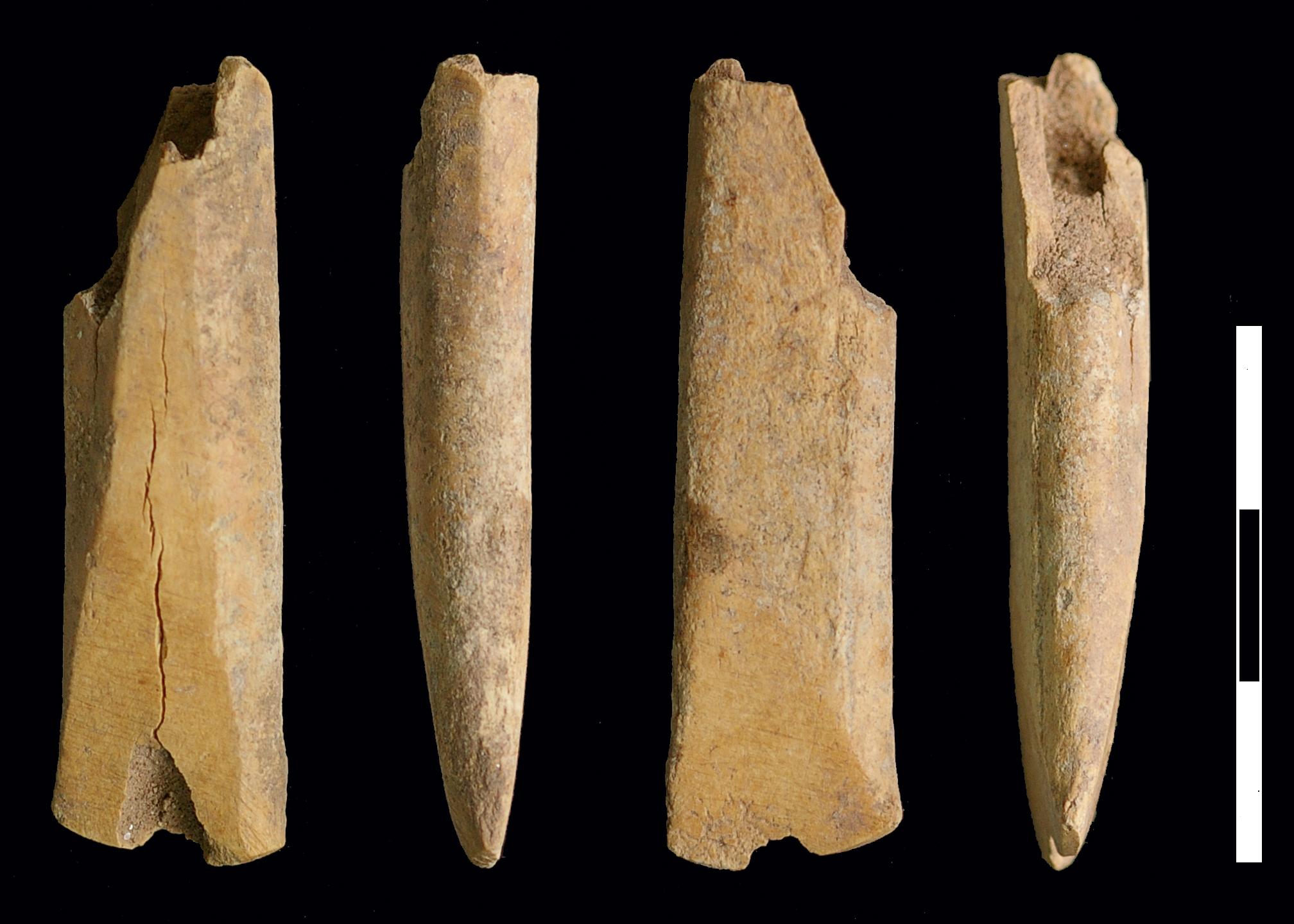 |

**Supplementary Figure S9.** Osseous tools dated to Early Neolithic phase Lepenski Vir III (see **Supplementary Fig. S5**). (A) OxA-16007 (7050±37 BP) dates pointed tool (inv. 336) found in a stone construction above building 8, spit 7. (B) Another pointed tool shown here is a flat symmetrical awl with half worked epiphysis found in the same context (the same inventory number) and clearly indicates a new, Neolithic *chaîne opératoire* that appears at Lepenski Vir for the first time during phase I-II and is dominant in phase III.

| A | B |
| --- | --- |
| 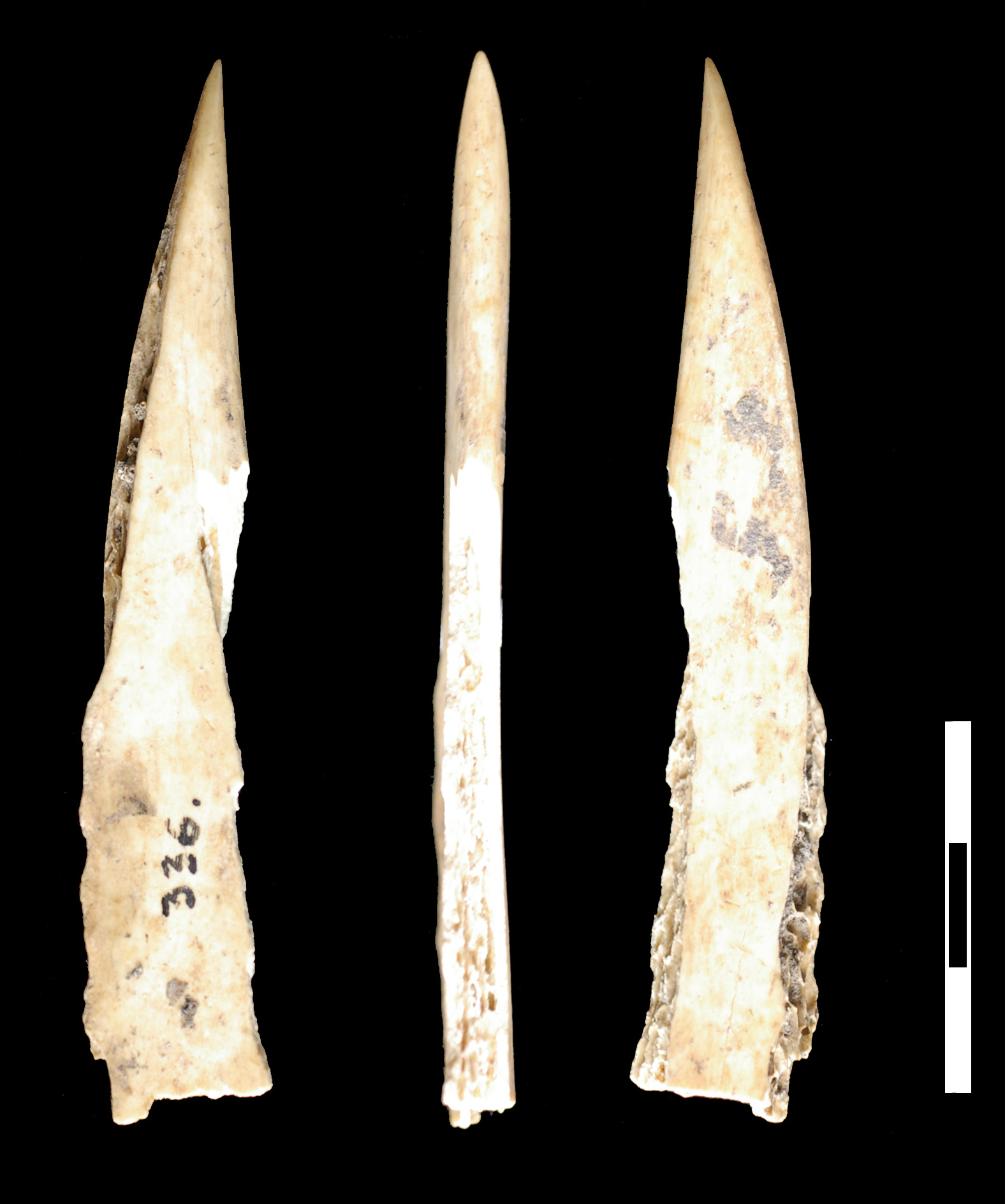 | 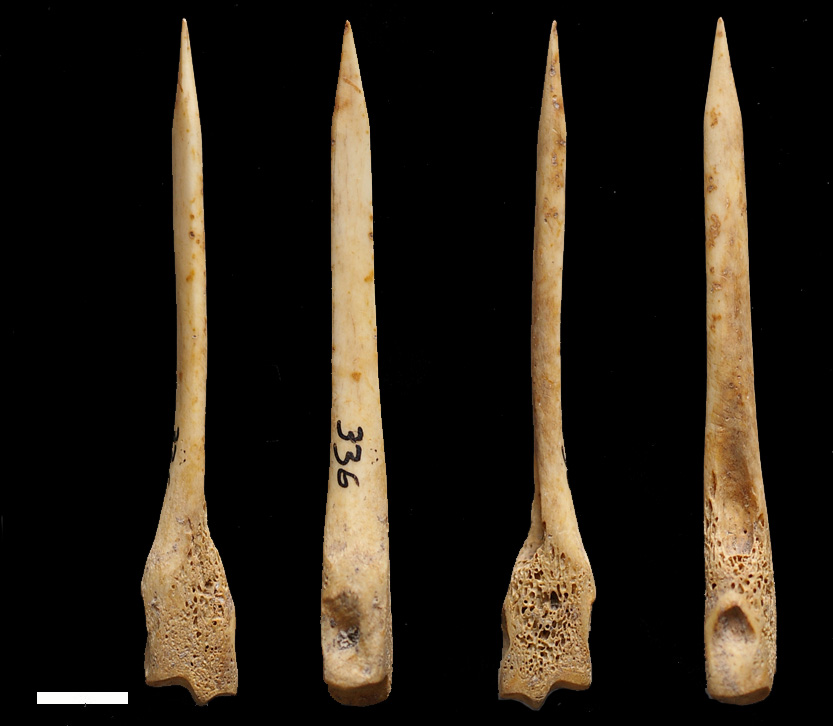 |

**Supplementary Table S3**: List of ornamented and aniconic boulders from Lepenski Vir. Catalogue numbers correspond with numbers on **Figure 1** in the main text of the article.

| **No.** | **Building – sculpture name** | | **Pigment colour(s)** | **Colour intensity** | **Coloured (body) part** | **Traces of fire** | **Weight (kg)** | **Dimensions**  **(cm)** | **Context** |
| --- | --- | --- | --- | --- | --- | --- | --- | --- | --- |
| 1 | H. 28 – Adam | | dark brown | hardly visible | neck & body | – | 5.11 | 16x13x23.2 | approx. above child burial (burial 92) – rear of building |
| 2 | H. 40 – Sirena [Mermaid] | | pale red | light | body & lips | – | 4.26 | 16x14x14.6 | above head of child (burial 61) – rear of building |
| 3 | H. 31 – Vrač [Shaman] | | red | light | relief parts | – | 8.5 | 18.5x16x22 | behind hearth |
| 4 | H. XXVIII – Hronos [Chronos] | | red | Hardly visible | relief parts | – | 21.86 | 23x19x36.5 | stone construction above building 23 |
| 5 | H. 24 – Sculpture | | – | – | – | black & red on the lower part | 13.6 | 19.5x18.5x24 | behind hearth |
| 6 | NA – Lady of Lepenski Vir | | red | light | relief parts of the face | – | 5.9 | 17.5x15.5x16 | backfilled earth (1966) |
| 7 | H. XLIV/57 – Rodonačelnik [Progenitor] | | red | light | neck & eye holes |  | 55 | 33x32x52 | behind hearth |
| 8 | H. XLIV/57 – Praroditeljka [First Mother] | | red | light | relief parts | red on the back | 38.5 | 39x27x51 | in front of hearth (lying on its back) |
| 9 | H. XLIV/57 – Danubius | | brown-black | ? | relief parts | – | 53 | 33x25x51 | in front of hearth |
| 10 | H. XLIV/57 – Praotac [Great Grandfather] | | red | light | relief parts & body | red & black on the back | 21.25 | 26x17x36 | in front of hearth |
| 11 | H. XLIV/57 – Sculpture | | – | – | – | – | 10.5 | 18x18x26 | NE (B) front corner of building |
| 12 | NA – Vodena vila [Water Fairy] | | red | light | relief parts | – | 23.1 | 26x19x38 | space between buildings – quad. a/5 |
| 13 | H. 54 - Black Obelisk | | brown-red | light | relief parts | black whole upper part | c. 50 | 27x23x52 | behind hearth |
| 14 | H. 54 - Black Obelisk | | pale red | patchy | relief parts | black whole upper part | c. 20 | 27x19x36 | behind hearth |
| 15 | H. 38 – Veliki beleg [Large Sign] | | red | light | relief parts | red strip | c. 50 | 37x29x48 | behind hearth |
| 16 | H. 28 – Sculpture | | – | – | – | the top – black | ? | 17.5x11x21.5 | rear of building – right side of the stone ‘table’ |
| 17 | H. 3 – Sculpture | | – | – | – | black traces | ? | 12.3x9.7x17.5 | left corner at the back of the building |
| 18 | H. 51† – Vulva | | – | – | – | intensive red | 3.2 | 13.8x11.2x18 | in front of hearth |
| 19 | H. 46 – Sculpture | | brown-red | light | surface | lower part | ? | 19x14x28 | ash place/in front of hearth |
| 20 | NA – Sculpture | | – | – | – | – | 3.4 | 14.2x12x16 | space between buildings quad. a/13, spit 8 |
| 21 | H. 50 – Sculpture | | – | – | – | – | 8.5 | 20x14.5x20.6 | behind hearth |
| 22 | H. 9 – Sculpture | | red | light | relief parts | 2 small zones | ? | 17x15x26 | behind hearth |
| 23 | H. 22 – Vilenjak [Elf] | | – | – | – | – | c. 25 | 41x17x27 | along right side of hearth |
| 24 | H. 6 – Stari poglavica  [Old Chief] | | red | light | relief parts | – | – |  | next to the building |
| 25 | H. 21 – Vodena buba [Water Bug] | | – | – | – | – | 4.6 | 15x13.5x19 | along left side of hearth |
| 26 | H. 37 – Sculpture – Somče [Catfish] | | red | light | back part | – | 4.7 | 14.2x12x19 | in building infill |
| 27 | H. 43 - Crvena skulptura [Red Sculpture] | | red | intensive | relief parts & strips extending ornam. grooves | decorated part–intensive | ? | 22x19x23 | taking the role of the rear stone of the hearth |
| 28 | H. 71 – Crveni znak [Red Mark] | | – | – | – | over ornam. part | ? | 20x12x26 | behind hearth |
| 29 | H. 25 – Sculpture | | red-brown | light | surface | gray-black(?) | ? |  | above head of burial 4 |
| 30 | H. 21 – Sculpture | | red | intensive | relief parts | red | ? | 15x10x18 | on forehead of burial 7/I |
| 31 | H. 16 – Beli znak [White Sign] | | – | – | – | – | ? | 23x20x34 | next to building [?] |
| 32 | H. 46 – Sculpture | | – | – | – | top – black & red | 10.6 | 21x14x27 | left side of building |
| 33 | H. VII – Sculpture | | – | – | – | – | ? |  | infill of building 39 (quad. A/10) |
| 34 | NA – Larva | | – | – | – | – | ? | 7x5.5x9 | space between buildings – quad. a/V, towards a/VI, W section, outside building (26/7/67) |
| 35 | NA – Šumski list [Forest Leaf] | | red | light | relief parts | – | 1.1 | 11x7x12 | space between buildings – quad. b/5 – disturbed layer |
| 36 | NA – Sculpture | | – | – | – | – | ? | 20x17x23 | space between buildings – quad. A/10 (spit 7 to southern section) |
| 37 | NA – Sculpture | | red | light | relief parts | top – black | ? | 16x12x21 | not *in situ* (?); H. VII |
| 38 | NA – Medaljon [Broach] | | red | very light | decorated fringes | – | 16.6 | 27x17x31 | backfilled earth (1966) |
| 39 | H. XLIV/57 – Sculpture | | – | – | – | black (?) | ? | 11.5x5x14.5 | beneath floor level |
| 40 | NA – Sculpture | | – | – | – | – | ? |  | backfilled earth (1966) |
| 41 | H. 54 – Sculpture | | – | – | – | black-gray | ? | 14x5.2x14.5 | floor area (?) |
| 42 | H. XXXIII –Varvarin [Barbarian] | | red | intensive | the neck | red | 18.6 | 26.3x14x45.5 | infill of building 48 |
| 43 | H. XVII – Jelen u šumi [Red Deer in Forest] | | red | light | relief parts | – | 50–70 | 38x21x62 cm | in front of building 19 |
| - | H. 40 – mortar | | ? | ? | ? | ? | 3.9 | 14.5x11x18.5 | behind hearth |
| *Aniconic boulders* | | | | | | | | | |
| 44 | H. 36 – Aniconic | | – | – | – | – | ? |  | behind hearth |
| 45 | H. 47’ – Aniconic | | – | – | – | – | ? |  | ash place/in front of hearth |
| 46 | H. 3 – Aniconic | | – | – | – | – | ? | 26x16x37 | behind hearth |
| 47 | H. 61 – Aniconic | | – | – | – | – | ? |  | ash place/in front of hearth |
| 48 | H. 33 – Aniconic | | – | – | – | – | ? |  | behind hearth |
| 49 | H. 65/XXXV – Aniconic | | – | – | – | – | ? |  | behind hearth of building 65 |
| 50 | H. 19 – Čuma | | – | – | – | – | ? |  | behind hearth |
| 51 | H. 22 – Aniconic | | – | – | – | – | ? |  | in front of hearth |
| 52 | H. XLIV/57 – Aniconic | | – | – | – | – | ? |  | behind hearth |
| 53 | H. XLIV/57 – Aniconic | | – | – | – | – | ? |  | behind hearth |
|  | | *Ornamented mortars* | | | | | | | |
| 54 | H. 33 – Jelen [Red Deer] | | red-brown | ? | non-ornament. part | – | 5.85 | 16.5x11.5x24 | behind hearth |
| 55 | H. 45 – Riba [Fish] | | – | – | – | burned head | 6.4 | 16.2x12.5x27 | behind hearth |
| 56 | H. 35 – Mortar | | – | – | – | – | ? |  | ash place/in front of hearth |
| 57 | H. 40 – Mortar [Animal head] | | red-brown | intensive | relief parts | – | 3.7 | 14x12x22 | behind hearth |
| 58 | H. 7 – Mortar [White Altar] | | – | – | – | – | 6.6 | 16.3x14x20 | behind hearth |
| 59 | H. 19 – Mortar | | – | – | – | – | ? | 12.5x9.5x18 | beneath floor |
| 60 | NA – Mortar | | red-brown | ? | relief parts | – | ? | 20.5x16x29 | space between buildings – quad. a/II, 1.35 m (24/7/67) |
| 61 | NA – Mortar | | – | – | – | – | ? | 12.5x9.8x17.5 | space between buildings – quad. A/VII, spit 7 (24/7/67) |
| 62 | H. XIX – Mortar | | – | – | – | – | 1.5 | 11.5x5.5x13.5 | behind hearth |
| 63 | H. 54 – Mortar | | – | – | – | – | ? |  | behind hearth |
| 64 | H. 37 – Mortar | | – | – | – | red – rim of the receptacle | 14.6 | 21x14.5x38 | behind hearth |
| 65 | H. 37 – Mortar | | red-brown | ? | buried portion-relief parts | - | 48.95 | 35x28x44 | behind hearth – lower portion buried in floor |
| 67 | H. 5 – Mortar | | – | – | – | burned surface | ? | 16.5x25.5 | from pile of stones within building(?) |
| *Aniconic mortars* | | | | | | | | | |
| 66 | H. 46 – Mortar | | – | – | – | burned on the upper surface | ? | 30x18x43 | behind hearth |
| 68 | H. XXXIII – Mortar | | – | – | – | – | 28.9 | 32x18x37 | behind hearth |
| 69 | H. 36 – Mortar | | – | – | – | – | ? |  | behind hearth |
| 70 | H. 41 – Mortar | | – | – | – | – | ? |  | behind hearth |
| 71 | H. 29 – Mortar | | – | – | – | – | ? |  | front left corner of building |
| 72 | H. 38 – Mortar | | – | – | – | – | ? | 18x14x20 | ash place/in front of hearth |
| 73 | H. 39 – Mortar | | – | – | – | – | ? |  | behind hearth |
| 74 | H. 7 – Mortar | | – | – | – | – | ? |  | within hearth |
| 75 | H. 9 – Mortar | | – | – | – | – | ? |  | behind hearth |
| 76 | H. 31 – Mortar | | – | – | – | – | ? |  | behind hearth |
| 77 | H. 51† – Mortar | | – | – | – | – | ? |  | behind hearth |
| 78 | H. 11 – Mortar | | – | – | – | – | ? |  | behind hearth |
| 79 | H. 4 – Mortar | | – | – | – | – | ? |  | behind hearth |
| 80 | H. 23 – Mortar | | – | – | – | – | ? |  | behind hearth |
| 81 | H. 32 – Mortar | | – | – | – | – | ? |  | behind hearth |
| 82 | H. 24 – Mortar | | – | – | – | – | ? |  | behind hearth |
| 83 | H. 3 – Mortar | | – | – | – | – | ? |  | front corner of building |
| 84 | H. XIV – Mortar | | – | – | – | – | ? |  | behind hearth [above the level of building 16] |
| 85a | H. 20 – Mortar | | – | – | – | – | ? |  | behind left corner of hearth |
| 85b | H. 20 – Mortar | | – | – | – | – | ? |  | behind right corner of hearth |
| 86 | H. 26’ – Mortar | | – | – | – | – | ? | 9.5x13.5x22 | on left side of hearth |
| 87 | H. 22 – Mortar | | – | – | – | – | 5 | 24.6x15x10.2 | behind hearth |
| 88 | NA – Mortar [?] | | – | – | – | – | ? | 14x11x23 | not *in situ* (?) |
| – | H. 13 – Mortar | | ? | ? | ? | ? | ? |  | behind hearth |
| – | H. 18 – Mortar | | ? | ? | ? | ? | ? |  | behind hearth |
| – | H. 64 – Mortar | | ? | ? | ? | ? | ? |  | back of building |
| – | H. 64 – Mortar | | ? | ? | ? | ? | ? |  | behind hearth |
| – | H. 62 – Sculpture | | ? | ? | ? | ? | ? | 10.5x11.5 | behind the head of burial 46 |
|  |  | |  |  |  |  |  |  |  |

**Supplementary text 5: The CQL codes for Models 1–3**

*The CQL code for Model 1 (all available measurements dating Mesolithic and Neolithic contexts from Lepenski Vir)*

Plot()

{

Outlier_Model("General",T(5),U(0,4),"t");

Outlier_Model("SSimple",N(0,2),0,"s");

Sequence()

{

Boundary("Start Early_Middle_Meso_Proto_LV");

Phase("Early_Middle_Meso_Proto_LV")

{

R_Date("C/I-II, charcoal Pinus: OxA-32863", 12335, 50)

{

Outlier("General", 0.05);

color="green";

};

R_Date("quad. A/3, spit 2, charcoal Pinus: OxA-32863", 10075, 45)

{

Outlier("General", 0.05);

color="green";

};

R_Date("beneath H.38: OxA-26552", 10035, 50)

{

Outlier("General", 0.05);

color="magenta";

};

R_Date("Burial 60: OxA-25092", 9549, 71)

{

Outlier("General", 0.05);

color="magenta";

};

Phase("between H.47")

{

R_Date("beneath H.47&47': OxA-16072", 9850, 50)

{

Outlier("General", 0.05);

color="magenta";

};

R_Date("beneath H.47': OxA-16004", 9730, 50)

{

Outlier("General", 0.05);

color="magenta";

};

};

R_Date("H.54-floor: OxA-16076", 9750, 45)

{

Outlier("General", 0.05);

color="magenta";

};

R_Date("Burial 50: BA-10651", 9082, 62)

{

Outlier("General", 0.05);

color="blue";

};

R_Date("Burial 69: OxA-25215", 8695, 64)

{

Outlier("General", 0.05);

color="blue";

};

R_Date("A/11, spit 6: OxA-26551", 8910, 45)

{

Outlier("General", 0.05);

color="magenta";

};

R_Date("C/XV, spit 19: OxA-26553", 8840, 45)

{

Outlier("General", 0.05);

color="magenta";

};

R_Date("beneath H.19: OxA-24771", 8871, 38)

{

Outlier("General", 0.05);

color="magenta";

};

R_Date("between H.26 & 26': OxA-16071", 8855, 45)

{

Outlier("General", 0.05);

color="magenta";

};

R_Date("beneath H.23: OxA-8610", 8770, 60)

{

Outlier("General", 0.05);

color="magenta";

};

Phase("Hearth 'a'")

{

R_Date("Burial 22: AA-57781", 8431, 78)

{

Outlier("General", 0.05);

color="magenta";

};

R_Date("shed red deer antler: OxA-16074", 8645, 40)

{

Outlier("General", 0.05);

color="magenta";

};

R_Date("red deer bone: OxA-24813", 8640, 40)

{

Outlier("General", 0.05);

color="magenta";

};

R_Combine("boar tusk tool")

{

Outlier("General", 0.05);

color="magenta";

R_Date("OxA-26549", 8659, 45)

{

Outlier("SSimple", 0.05);

};

R_Date("OxA-26550", 8710, 45)

{

Outlier("SSimple", 0.05);

};

};

R_Date("beneath H.31: OxA-24812", 8410, 39)

{

Outlier("General", 0.05);

color="magenta";

};

R_Date("b/13, stone const.: OxA-26548", 8265, 45)

{

Outlier("General", 0.05);

color="magenta";

};

};

Interval("Duration Early_Middle_Meso_Proto_LV");

};

Boundary("End Early_Middle_Meso_Proto_LV");

Boundary("Start Transition_LV_I-II");

Phase("Transition_LV_I-II")

{

Phase("H.37-floor")

{

R_Date("H.37, bone tool: OxA-16082", 7138, 37)

{

Outlier("General", 0.05);

color="magenta";

};

R_Combine("H.37_beam1, charcoal")

{

Outlier("General", 0.05);

color="green";

R_Date("OxA-32886", 7156, 36)

{

Outlier("SSimple", 0.05);

};

R_Date("OxA-32887", 7191, 35)

{

Outlier("SSimple", 0.05);

};

};

R_Combine("H.37_beam2, charcoal")

{

Outlier("General", 0.05);

color="green";

R_Date("Bln-649", 6800, 100)

{

Outlier("SSimple", 0.05);

};

R_Date("Bln-678", 6900, 100)

{

Outlier("SSimple", 0.05);

};

R_Date("BM-379", 6900, 150)

{

Outlier("SSimple", 0.05);

};

};

};

Phase("H.62")

{

R_Date("H.62, charcoal: OxA-32865", 7176, 36)

{

Outlier("General", 0.05);

color="green";

};

R_Date("H.62, charcoal: KN-405", 7430, 160)

{

Outlier("General", 0.05);

color="green";

};

};

Sequence("H.20,33 & 32")

{

Phase("between H.20 & 33")

{

R_Date("between H.20 & 33: OxA-8725", 7380, 93)

{

Outlier("General", 0.05);

color="magenta";

};

R_Date("between H.20 & 33: OxA-15998", 7280, 45)

{

Outlier("General", 0.05);

color="magenta";

};

};

Phase("H.32")

{

R_Date("H.32-floor: OxA-15999", 7111, 40)

{

Outlier("General", 0.05);

color="magenta";

};

R_Date("H.32, charcoal: P-1598", 6814, 69)

{

Outlier("General", 0.05);

color="green";

};

};

};

Sequence("H.26 & 26'")

{

R_Combine("between H.26 & 26’: OxA-16001, OxA-16002")

{

Outlier("General", 0.05);

color="magenta";

R_Date("OxA-16001", 7235, 40)

{

Outlier("SSimple", 0.05);

};

R_Date("OxA-16002", 7160, 40)

{

Outlier("SSimple", 0.05);

};

};

R_Date("H.26-floor, red deer skull w/antlers: OxA-16000", 7070, 40)

{

Outlier("General", 0.05);

color="magenta";

};

};

Sequence("H.47,47' & Burial 122")

{

R_Combine("Burial 122, between H.47 & 47': OxA-16005, OxA-16006")

{

Outlier("General", 0.05);

color="blue";

R_Date("OxA-16005", 7190, 45)

{

Outlier("SSimple", 0.05);

};

R_Date("OxA-16006", 7190, 40)

{

Outlier("SSimple", 0.05);

};

};

R_Date("H.47-floor: UCLA-1407", 6970, 60)

{

Outlier("General", 0.05);

color="green";

};

};

Sequence("H.51 & 57/XLIV")

{

R_Date("Between H.51 & 57/XLIV: OxA-8618", 7200, 60)

{

Outlier("General", 0.05);

color="magenta";

};

Phase("H.57/XLIV floor")

{

R_Date("OxA-16081", 7219, 37)

{

Outlier("General", 0.05);

color="magenta";

};

R_Date("OxA-16073", 7125, 40)

{

Outlier("General", 0.05);

color="magenta";

};

R_Date("Burial 19(3): OxA-16008", 7092, 42)

{

Outlier("General", 0.05);

color="blue";

};

};

};

Phase("H.34 & Burial 26")

{

R_Date("H.34_floor: OxA-16009",7165, 40)

{

Outlier("General", 0.05);

color="magenta";

};

R_Combine("Burial 26: AA-57782, OxA-16002")

{

Outlier("General", 0.05);

color="blue";

R_Date("Burial 26: AA-57782", 7122, 57)

{

Outlier("SSimple", 0.05);

};

R_Date("Burial 26: OxA-25206", 7058, 37)

{

Outlier("SSimple", 0.05);

};

};

R_Date("H.34, charcoal: Bln-650", 6820, 100)

{

color="green";

};

};

Sequence("H.24 & Burials 94 and 100")

{

R_Date("Burial 100, beneath H.24: OxA-34519", 7333, 62)

{

Outlier("General", 0.05);

color="blue";

};

R_Date("Hearth of H.24: X-2176-18", 7285, 45)

{

Outlier("General", 0.05);

color="magenta";

};

R_Date("Burial 94, H.24: OxA-16010", 6980, 81)

{

Outlier("General", 0.05);

color="blue";

};

};

Phase("H.65/XXXV & Burials 54a-e")

{

R_Date("H.65/XXXV: X-2176-19", 7314, 40)

{

Outlier("General", 0.05);

color="magenta";

};

R_Combine("Burial 54d: AA-57783, OxA-25213")

{

Outlier("General", 0.05);

color="blue";

R_Date("AA-57783", 7067, 55)

{

Outlier("SSimple", 0.05);

};

R_Date("OxA-25213", 7269, 68)

{

Outlier("SSimple", 0.05);

};

};

R_Date("Burial 54c: OxA-25209", 7169, 52)

{

Outlier("General", 0.05);

color="blue";

};

R_Date("Burial 54e: OxA-25210", 7155, 54)

{

Outlier("General", 0.05);

color="blue";

};

};

Sequence("H.21 & 22_Burials_7/I & II")

{

R_Date("H.22_floor_red deer skull: OxA-16075", 7157, 39)

{

Outlier("General", 0.05);

color="magenta";

};

Phase("Burial 7/I & II, red deer skull, H.21")

{

R_Combine("Burial 7/I: AA-57779, OxA-25204, OxA-25205")

{

Outlier("General", 0.05);

color="blue";

R_Date("AA-57779", 7157, 79)

{

Outlier("SSimple", 0.05);

};

R_Date("OxA-25204", 7224, 72)

{

Outlier("SSimple", 0.05);

};

R_Date("OxA-25205", 7203, 73)

{

Outlier("SSimple", 0.05);

};

};

R_Date("Burial 7/II: AA-57780", 7031, 95)

{

Outlier("General", 0.05);

color="blue";

};

R_Date("red deer skull w/antlers: OxA-16537", 6924, 37)

{

Outlier("General", 0.05);

color="magenta";

};

R_Date("aurochs skull: OxA-32933", 7133, 37)

{

Outlier("General", 0.05);

color="magenta";

};

};

};

Sequence("H.54")

{

R_Date("H.54-beneath the floor-ivory tool: OxA-26547", 7396, 40)

{

Outlier("General", 0.05);

color="magenta";

};

Phase("H.54-floor, charcoal: Bln-649, Bln-678, BM-379")

{

R_Date("H.54: Z-115", 6984, 94)

{

Outlier("General", 0.05);

color="green";

};

R_Date("H.54: Bln-653", 7040, 100)

{

Outlier("General", 0.05);

color="green";

};

R_Date("H.54: Bln-738", 7225, 100)

{

Outlier("General", 0.05);

color="green";

};

R_Date("H.54: KN-407", 7280, 160)

{

Outlier("General", 0.05);

color="green";

};

};

};

Phase("H.27-floor")

{

R_Date("H.27: OxA-16077", 7225, 40)

{

Outlier("General", 0.05);

color="magenta";

};

R_Date("H.27, charcoal: KN-406", 7210, 200)

{

Outlier("General", 0.05);

color="green";

};

};

Phase("between H.35 & 36")

{

R_Combine("charcoal, timber between H.35 & 36:Bln-740a, Bln-740b")

{

Outlier("General", 0.05);

color="green";

R_Date("Bln-740a", 7310, 100)

{

Outlier("SSimple", 0.05);

};

R_Date("Bln-740b", 7360, 100)

{

Outlier("SSimple", 0.05);

};

};

R_Date("between H.35 & 36: OxA-16003", 7170, 40)

{

Outlier("General", 0.05);

color="magenta";

};

};

Sequence("H.4")

{

R_Date("OxA-26554: bone tool, beneath the floor", 7462, 44)

{

Outlier("General", 0.05);

color="magenta";

};

Phase("H.4-floor")

{

R_Date("OxA-16084: bone tool", 7285, 37)

{

Outlier("General", 0.05);

color="magenta";

};

R_Date("fish hook: OxA-27901", 7207, 35)

{

Outlier("General", 0.05);

color="magenta";

};

};

};

Sequence("H.40_Burials_61 & 21")

{

R_Date("Burial 61, H.40: OxA-25211", 7179, 73)

{

Outlier("General", 0.05);

color="blue";

};

R_Date("Burial 21, H.40: OxA-34968", 7080, 42)

{

Outlier("General", 0.05);

color="blue";

};

};

R_Date("H.16, charcoal: Bln-576", 6820, 100)

{

Outlier("General", 0.05);

color="green";

};

R_Date("H.1, charcoal: Bln-575", 6860, 100)

{

Outlier("General", 0.05);

color="green";

};

R_Date("H.9, charcoal: Bln-647", 6845, 100)

{

Outlier("General", 0.05);

color="green";

};

R_Date("H.28-floor, red deer skull: OxA-16078", 7191, 40)

{

Outlier("General", 0.05);

color="magenta";

};

R_Date("H.5, bone tool: OxA-16083", 7059, 36)

{

Outlier("General", 0.05);

color="magenta";

};

R_Date("Burial 45b, H.61/XXXIV: OxA-25214", 7289, 69)

{

Outlier("General", 0.05);

color="blue";

};

R_Date("Burial 14, group burial: OxA-25090", 7226, 72)

{

Outlier("General", 0.05);

color="blue";

};

R_Date("Burial 93: BA-10651", 7123, 89)

{

Outlier("General", 0.05);

color="blue";

};

R_Date("Burial 89a: OxA-25089", 7046, 71)

{

Outlier("General", 0.05);

color="blue";

};

R_Date("Burial 44: OxA5830", 7153, 106)

{

Outlier("General", 0.05);

color="blue";

};

R_Date("Burial 31a: OxA-5827", 7311, 108)

{

Outlier("General", 0.05);

color="blue";

};

R_Date("Burial 79a: OxA-25091", 7119, 74)

{

Outlier("General", 0.05);

color="blue";

};

R_Date("H.51-floor, charcoal: Bln-652", 6620, 100)

{

Outlier("General", 0.05);

color="green";

};

Interval("Duration Transition_LV_I-II");

};

Boundary("End Transition_LV_I-II");

Boundary("Start Early_Neo_LV_III");

Phase("Early_Neo_LV_III")

{

R_Date("H.IX, charcoal: Bln-654", 6630, 100)

{

Outlier("General", 0.05);

color="green";

};

R_Date("H.XXXII, charcoal: Bln-655", 6560, 100)

{

Outlier("General", 0.05);

color="green";

};

R_Date("above H.8-bone tool: OxA-16007", 7050, 40)

{

Outlier("General", 0.05);

color="magenta";

};

R_Date("d/3, spit 6, goat: OxA-16212", 7041, 35)

{

Outlier("General", 0.05);

color="magenta";

};

R_Date("C/XVI, spit 3, goat: OxA-16253", 7008, 38)

{

Outlier("General", 0.05);

color="magenta";

};

R_Date("c/I, spit 7, domestic cattle: OxA-16213", 7043, 37)

{

Outlier("General", 0.05);

color="magenta";

};

R_Date("Pit 1, domestic pig: OxA-16079", 7037, 39)

{

Outlier("General", 0.05);

color="magenta";

};

R_Date("Pit 3, domestic cattle: OxA-16211", 7021, 36)

{

Outlier("General", 0.05);

color="magenta";

};

R_Date("Burial 5-bone tool: OxA-16538", 7136, 37)

{

Outlier("General", 0.05);

color="magenta";

};

R_Combine("Burial 8, above H.24: AA-58319, OxA-25207")

{

Outlier("General", 0.05);

color="blue";

R_Date("AA-58319", 6690, 54)

{

Outlier("SSimple", 0.05);

};

R_Date("OxA-25207", 6984, 39)

{

Outlier("SSimple", 0.05);

};

};

R_Date("Burial 9, above H.24: OxA-25208", 6893, 45)

{

Outlier("General", 0.05);

color="blue";

};

R_Date("Burial 32: OxA-5828", 7032, 95)

{

Outlier("General", 0.05);

color="blue";

};

R_Date("Burial 88: OxA-5831", 6979, 92)

{

Outlier("General", 0.05);

color="blue";

};

R_Date("Burial 35: OxA-5829", 6721, 93)

{

Outlier("General", 0.05);

color="blue";

};

R_Date("Burial 73: BA-10652", 6973, 48)

{

Outlier("General", 0.05);

color="blue";

};

R_Date("Burial 17: AA-58320", 6829, 53)

{

Outlier("General", 0.05);

color="blue";

};

Interval("Duration Early_Neo_LV_III");

};

Boundary("End Early_Neo_LV_III");

};

};

*The CQL code for Model 2 (all available dates with the exclusion of charcoal measurements dating Mesolithic and Neolithic contexts from Lepenski Vir)*

Plot()

{

Outlier_Model("General",T(5),U(0,4),"t");

Outlier_Model("SSimple",N(0,2),0,"s");

Sequence()

{

Boundary("Start Early-Middle_Meso_Proto_LV");

Phase("Early-Middle_Meso_Proto_LV")

{

R_Date("beneath H.38: OxA-26552", 10035, 50)

{

Outlier("General", 0.05);

color="magenta";

};

R_Date("Burial 60: OxA-25092", 9549, 71)

{

Outlier("General", 0.05);

color="blue";

};

Phase("between H.47")

{

R_Date("beneath H.47&47': OxA-16072", 9850, 50)

{

Outlier("General", 0.05);

color="magenta";

};

R_Date("beneath H.47': OxA-16004", 9730, 50)

{

Outlier("General", 0.05);

color="magenta";

};

};

R_Date("H.54-floor: OxA-16076", 9750, 45)

{

Outlier("General", 0.05);

color="magenta";

};

R_Date("Burial 50: BA-10651", 9082, 62)

{

Outlier("General", 0.05);

color="blue";

};

R_Date("Burial 69: OxA-25215", 8695, 64)

{

Outlier("General", 0.05);

color="blue";

};

R_Date("A/11, spit 6: OxA-26551", 8910, 45)

{

Outlier("General", 0.05);

color="magenta";

};

R_Date("C/XV, spit 19: OxA-26553", 8840, 45)

{

Outlier("General", 0.05);

color="magenta";

};

R_Date("beneath H.19: OxA-24771", 8871, 38)

{

Outlier("General", 0.05);

color="magenta";

};

R_Date("between H.26 & 26': OxA-16071", 8855, 45)

{

Outlier("General", 0.05);

color="magenta";

};

R_Date("beneath H.23: OxA-8610", 8770, 60)

{

Outlier("General", 0.05);

color="magenta";

};

Phase("Hearth 'a'")

{

R_Date("Burial 22: AA-57781", 8431, 78)

{

Outlier("General", 0.05);

color="magenta";

};

R_Date("shed red deer antler: OxA-16074", 8645, 40)

{

Outlier("General", 0.05);

color="magenta";

};

R_Date("red deer bone: OxA-24813", 8640, 40)

{

Outlier("General", 0.05);

color="magenta";

};

R_Combine("boar tusk tool")

{

Outlier("General", 0.05);

color="magenta";

R_Date("OxA-26549", 8659, 45)

{

Outlier("SSimple", 0.05);

};

R_Date("OxA-26550", 8710, 45)

{

Outlier("SSimple", 0.05);

};

};

R_Date("beneath H.31: OxA-24812", 8410, 39)

{

Outlier("General", 0.05);

color="magenta";

};

R_Date("b/13, stone const.: OxA-26548", 8265, 45)

{

Outlier("General", 0.05);

color="magenta";

};

};

Interval("Duration Early-Middle_Meso_Proto_LV");

};

Boundary("End Early-Middle_Meso_Proto_LV");

Boundary("Start Transition_LV_I-II");

Phase("Transition_LV_I-II")

{

R_Date("H.37, bone tool: OxA-16082", 7138, 37)

{

Outlier("General", 0.05);

color="magenta";

};

Sequence("H.20,33 & 32")

{

Phase("between H.20 & 33")

{

R_Date("between H.20 & 33: OxA-8725", 7380, 93)

{

Outlier("General", 0.05);

color="magenta";

};

R_Date("between H.20 & 33: OxA-15998", 7280, 45)

{

Outlier("General", 0.05);

color="magenta";

};

};

R_Date("H.32-floor: OxA-15999", 7111, 40)

{

Outlier("General", 0.05);

color="magenta";

};

};

Sequence("H.26 & 26'")

{

R_Combine("between H.26 & 26’: OxA-16001, OxA-16002")

{

Outlier("General", 0.05);

color="magenta";

R_Date("OxA-16001", 7235, 40)

{

Outlier("SSimple", 0.05);

};

R_Date("OxA-16002", 7160, 40)

{

Outlier("SSimple", 0.05);

};

};

R_Date("H.26-floor, red deer skull w/antlers: OxA-16000", 7070, 40)

{

Outlier("General", 0.05);

color="magenta";

};

};

R_Combine("Burial 122, between H.47 & 47': OxA-16005, OxA-16006")

{

Outlier("General", 0.05);

color="blue";

R_Date("OxA-16005", 7190, 45)

{

Outlier("SSimple", 0.05);

};

R_Date("OxA-16006", 7190, 40)

{

Outlier("SSimple", 0.05);

};

};

Sequence("H.51 & 57/XLIV")

{

R_Date("Between H.51 & 57/XLIV: OxA-8618", 7200, 60)

{

Outlier("General", 0.05);

color="magenta";

};

Phase("H.57/XLIV floor")

{

R_Date("OxA-16081", 7219, 37)

{

Outlier("General", 0.05);

color="magenta";

};

R_Date("OxA-16073", 7125, 40)

{

Outlier("General", 0.05);

color="magenta";

};

R_Date("Burial 19(3): OxA-16008", 7092, 42)

{

Outlier("General", 0.05);

color="blue";

};

};

};

Phase("H.34 & Burial 26")

{

R_Date("H.34_floor: OxA-16009",7165, 40)

{

Outlier("General", 0.05);

color="magenta";

};

R_Combine("Burial 26: AA-57782, OxA-16002")

{

Outlier("General", 0.05);

color="blue";

R_Date("Burial 26: AA-57782", 7122, 57)

{

Outlier("SSimple", 0.05);

};

R_Date("Burial 26: OxA-25206", 7058, 37)

{

Outlier("SSimple", 0.05);

};

};

};

Sequence("H.24 & Burials 94 and 100")

{

R_Date("Burial 100, beneath H.24: OxA-34519", 7333, 62)

{

Outlier("General", 0.05);

color="blue";

};

R_Date("Hearth of H.24: X-2176-18", 7285, 45)

{

Outlier("General", 0.05);

color="magenta";

};

R_Date("Burial 94, H.24: OxA-16010", 6980, 81)

{

Outlier("General", 0.05);

color="blue";

};

};

Phase("H.65/XXXV & Burials 54a-e")

{

R_Date("H.65/XXXV: X-2176-19", 7314, 40)

{

Outlier("General", 0.05);

color="magenta";

};

R_Combine("Burial 54d: AA-57783, OxA-25213")

{

Outlier("General", 0.05);

color="blue";

R_Date("AA-57783", 7067, 55)

{

Outlier("SSimple", 0.05);

};

R_Date("OxA-25213", 7269, 68)

{

Outlier("SSimple", 0.05);

};

};

R_Date("Burial 54c: OxA-25209", 7169, 52)

{

Outlier("General", 0.05);

color="blue";

};

R_Date("Burial 54e: OxA-25210", 7155, 54)

{

Outlier("General", 0.05);

color="blue";

};

};

Sequence("H.21 & 22_Burials_7/I & II")

{

R_Date("H.22_floor_red deer skull: OxA-16075", 7157, 39)

{

Outlier("General", 0.05);

color="magenta";

};

Phase("Burial 7/I & II, red deer skull, H.21")

{

R_Combine("Burial 7/I: AA-57779, OxA-25204, OxA-25205")

{

Outlier("General", 0.05);

color="blue";

R_Date("AA-57779", 7157, 79)

{

Outlier("SSimple", 0.05);

};

R_Date("OxA-25204", 7224, 72)

{

Outlier("SSimple", 0.05);

};

R_Date("OxA-25205", 7203, 73)

{

Outlier("SSimple", 0.05);

};

};

R_Date("Burial 7/II: AA-57780", 7031, 95)

{

Outlier("General", 0.05);

color="blue";

};

R_Date("red deer skull w/antlers: OxA-16537", 6924, 37)

{

Outlier("General", 0.05);

color="magenta";

};

R_Date("aurochs skull: OxA-32933", 7133, 37)

{

Outlier("General", 0.05);

color="magenta";

};

};

};

R_Date("H.54-beneath the floor-ivory tool: OxA-26547", 7396, 40)

{

Outlier("General", 0.05);

color="magenta";

};

R_Date("H.27-floor: OxA-16077", 7225, 40)

{

Outlier("General", 0.05);

color="magenta";

};

R_Date("between H.35 & 36: OxA-16003", 7170, 40)

{

Outlier("General", 0.05);

color="magenta";

};

Sequence("H.4")

{

R_Date("OxA-26554: bone tool, beneath the floor", 7462, 44)

{

Outlier("General", 0.05);

color="magenta";

};

Phase("H.4-floor")

{

R_Date("OxA-16084: bone tool", 7285, 37)

{

Outlier("General", 0.05);

color="magenta";

};

R_Date("fish hook: OxA-27901", 7207, 35)

{

Outlier("General", 0.05);

color="magenta";

};

};

};

Sequence("H.40_Burials_61 & 21")

{

R_Date("Burial 61, H.40: OxA-25211", 7179, 73)

{

Outlier("General", 0.05);

color="blue";

};

R_Date("Burial 21, H.40: OxA-34968", 7080, 42)

{

Outlier("General", 0.05);

color="blue";

};

};

R_Date("H.28-floor, red deer skull: OxA-16078", 7191, 40)

{

Outlier("General", 0.05);

color="magenta";

};

R_Date("H.5, bone tool: OxA-16083", 7059, 36)

{

Outlier("General", 0.05);

color="magenta";

};

R_Date("Burial 45b, H.61/XXXIV: OxA-25214", 7289, 69)

{

Outlier("General", 0.05);

color="blue";

};

R_Date("Burial 14, group burial: OxA-25090", 7226, 72)

{

Outlier("General", 0.05);

color="blue";

};

R_Date("Burial 93: BA-10651", 7123, 89)

{

Outlier("General", 0.05);

color="blue";

};

R_Date("Burial 89a: OxA-25089", 7046, 71)

{

Outlier("General", 0.05);

color="blue";

};

R_Date("Burial 44: OxA5830", 7153, 106)

{

Outlier("General", 0.05);

color="blue";

};

R_Date("Burial 31a: OxA-5827", 7311, 108)

{

Outlier("General", 0.05);

color="blue";

};

R_Date("Burial 79a: OxA-25091", 7119, 74)

{

Outlier("General", 0.05);

color="blue";

};

Interval("Duration Transition_LV_I-II");

};

Boundary("End Transition_LV_I-II");

Boundary("Start Early_Neo_LV_III");

Phase("Early_Neo_LV_III")

{

R_Date("above H.8-bone tool: OxA-16007", 7050, 40)

{

Outlier("General", 0.05);

color="magenta";

};

R_Date("d/3, spit 6, goat: OxA-16212", 7041, 35)

{

Outlier("General", 0.05);

color="magenta";

};

R_Date("C/XVI, spit 3, goat: OxA-16253", 7008, 38)

{

Outlier("General", 0.05);

color="magenta";

};

R_Date("c/I, spit 7, domestic cattle: OxA-16213", 7043, 37)

{

Outlier("General", 0.05);

color="magenta";

};

R_Date("Pit 1, domestic pig: OxA-16079", 7037, 39)

{

Outlier("General", 0.05);

color="magenta";

};

R_Date("Pit 3, domestic cattle: OxA-16211", 7021, 36)

{

Outlier("General", 0.05);

color="magenta";

};

R_Date("Burial 5-bone tool: OxA-16538", 7136, 37)

{

Outlier("General", 0.05);

color="magenta";

};

R_Combine("Burial 8, above H.24: AA-58319, OxA-25207")

{

Outlier("General", 0.05);

color="blue";

R_Date("AA-58319", 6690, 54)

{

Outlier("SSimple", 0.05);

};

R_Date("OxA-25207", 6984, 39)

{

Outlier("SSimple", 0.05);

};

};

R_Date("Burial 9, above H.24: OxA-25208", 6893, 45)

{

Outlier("General", 0.05);

color="blue";

};

R_Date("Burial 32: OxA-5828", 7032, 95)

{

Outlier("General", 0.05);

color="blue";

};

R_Date("Burial 88: OxA-5831", 6979, 92)

{

Outlier("General", 0.05);

color="blue";

};

R_Date("Burial 35: OxA-5829", 6721, 93)

{

Outlier("General", 0.05);

color="blue";

};

R_Date("Burial 73: BA-10652", 6973, 48)

{

Outlier("General", 0.05);

color="blue";

};

R_Date("Burial 17: AA-58320", 6829, 53)

{

Outlier("General", 0.05);

color="blue";

};

Interval("Duration Early_Neo_LV_III");

};

Boundary("End Early_Neo_LV_III");

};

};

*The CQL code for Model 3 (only articulated human and animal remains dating Mesolithic and Neolithic contexts from Lepenski Vir)*

Plot()

{

Outlier_Model("General",T(5),U(0,4),"t");

Outlier_Model("SSimple",N(0,2),0,"s");

Sequence()

{

Boundary("Start Early-Middle_Meso_Proto_LV");

Phase("Early-Middle_Meso_Proto_LV")

{

R_Date("Burial 60: OxA-25092", 9549, 71)

{

Outlier("General", 0.05);

color="blue";

};

R_Date("Burial 50: BA-10651", 9082, 62)

{

Outlier("General", 0.05);

color="blue";

};

R_Date("Burial 69: OxA-25215", 8695, 64)

{

Outlier("General", 0.05);

color="blue";

};

R_Date("articulated bear, beneath H.31: OxA-24812", 8410, 39)

{

Outlier("General", 0.05);

color="magenta";

};

Interval("Duration Early-Middle_Meso_Proto_LV");

};

Boundary("End Early-Middle_Meso_Proto_LV");

Boundary("Start Transition_LV_I-II");

Phase("Transition_LV_I-II")

{

R_Date("H.26, red deer skull w/antlers: OxA-16000", 7070, 40)

{

Outlier("General", 0.05);

color="magenta";

};

R_Combine("Burial 122, between H.47 & 47': OxA-16005, OxA-16006")

{

Outlier("General", 0.05);

color="blue";

R_Date("OxA-16005", 7190, 45)

{

Outlier("SSimple", 0.05);

};

R_Date("OxA-16006", 7190, 40)

{

Outlier("SSimple", 0.05);

};

};

R_Combine("Burial 26, H.34: AA-57782, OxA-16002")

{

Outlier("General", 0.05);

color="blue";

R_Date("Burial 26: AA-57782", 7122, 57)

{

Outlier("SSimple", 0.05);

};

R_Date("Burial 26: OxA-25206", 7058, 37)

{

Outlier("SSimple", 0.05);

};

};

Sequence("H.24")

{

R_Date("Burial 100, beneath H.24: OxA-34519", 7333, 62)

{

Outlier("General", 0.05);

color="blue";

};

R_Date("Burial 94, through the floor-H.24: OxA-16010", 6980, 81)

{

Outlier("General", 0.05);

color="blue";

};

};

Phase("H.65/XXXV & Burials 54a-e")

{

R_Combine("Burial 54d: AA-57783, OxA-25213")

{

Outlier("General", 0.05);

color="blue";

R_Date("AA-57783", 7067, 55)

{

Outlier("SSimple", 0.05);

};

R_Date("OxA-25213", 7269, 68)

{

Outlier("SSimple", 0.05);

};

};

R_Date("Burial 54c: OxA-25209", 7169, 52)

{

Outlier("General", 0.05);

color="blue";

};

R_Date("Burial 54e: OxA-25210", 7155, 54)

{

Outlier("General", 0.05);

color="blue";

};

};

Sequence("H.21 & 22_Burials_7/I&II")

{

R_Date("H.22_floor_red deer skull: OxA-16075", 7157, 39)

{

color="magenta";

};

Phase("Burial 7/I&II, red deer and aurochs skulls, H.21")

{

R_Combine("Burial 7/I: AA-57779, OxA-25204, OxA-25205")

{

Outlier("General", 0.05);

color="blue";

R_Date("AA-57779", 7157, 79)

{

Outlier("SSimple", 0.05);

};

R_Date("OxA-25204", 7224, 72)

{

Outlier("SSimple", 0.05);

};

R_Date("OxA-25205", 7203, 73)

{

Outlier("SSimple", 0.05);

};

};

R_Date("Burial 7/II: AA-57780", 7031, 95)

{

Outlier("General", 0.05);

color="blue";

};

R_Date("red deer skull w/antlers: OxA-16537", 6924, 37)

{

Outlier("General", 0.05);

color="magenta";

};

R_Date("aurochs skull: OxA-32933", 7133, 37)

{

Outlier("General", 0.05);

color="magenta";

};

};

};

R_Date("H.28-floor, red deer skull: OxA-16078", 7191, 40)

{

Outlier("General", 0.05);

color="magenta";

};

R_Date("Burial 45b, H.61/XXXIV: OxA-25214", 7289, 69)

{

Outlier("General", 0.05);

color="blue";

};

R_Date("Burial 61, H.40: OxA-25211", 7179, 73)

{

Outlier("General", 0.05);

color="blue";

};

R_Date("Burial 14, group burial: OxA-25090", 7226, 72)

{

Outlier("General", 0.05);

color="blue";

};

R_Date("Burial 93: BA-10651", 7123, 89)

{

Outlier("General", 0.05);

color="blue";

};

R_Date("Burial 89a: OxA-25089", 7046, 71)

{

Outlier("General", 0.05);

color="blue";

};

Interval("Duration Transition_LV_I-II");

};

Boundary("End Transition_LV_I-II");

Boundary("Start Early_Neo_LV_III");

Phase("Early_Neo_LV_III")

{

R_Combine("Burial 8, above H.24: AA-58319, OxA-25207")

{

Outlier("General", 0.05);

color="blue";

R_Date("AA-58319", 6690, 54)

{

Outlier("SSimple", 0.05);

};

R_Date("OxA-25207", 6984, 39)

{

Outlier("SSimple", 0.05);

};

};

R_Date("Burial 9, above H.24: OxA-25208", 6893, 45)

{

Outlier("General", 0.05);

color="blue";

};

R_Date("Burial 32: OxA-5828", 7032, 95)

{

Outlier("General", 0.05);

color="blue";

};

R_Date("Burial 88: OxA-5831", 6979, 92)

{

Outlier("General", 0.05);

color="blue";

};

R_Date("Burial 73: BA-10652", 6973, 48)

{

Outlier("General", 0.05);

color="blue";

};

R_Date("Burial 17: AA-58320", 6829, 53)

{

Outlier("General", 0.05);

color="blue";

};

Interval("Duration Early_Neo_LV_III");

};

Boundary("End Early_Neo_LV_III");

};

};

**SI References**

1. Cook, G. *et al.* Problems of dating human bones from the Iron Gates. *Antiquity* **76**, 77–85 (2002).

2. Cook, G. *et al.* A freshwater diet-derived ^14^C reservoir effect at the Stone Age sites in the Iron Gates gorge. *Radiocarbon* **43**(2A), 453–60 (2001).

3. Bronk Ramsey, C. Radiocarbon calibration and analysis of stratigraphy: the OxCal Program. *Radiocarbon* **37**(2), 425–430 (1995).

4. Bronk Ramsey, C. Development of the radiocarbon program OxCal. *Radiocarbon* **43**(2A), 355–363 (2001).

5. Bonsall, C. *et al.* New AMS ^14^C dates for human remains from Stone Age sites in the Iron Gates reach of the Danube, southeast Europe. *Radiocarbon* **57(1)**, 33–46 (2015).

6. Borić, D. & Dimitrijević, V. Absolute chronology and stratigraphy of Lepenski Vir. *Starinar* **57**/2007, 9–55 (2009).

7. Borić, D. & Price, T. D. Strontium isotopes document greater human mobility at the start of the Balkan Neolithic. *Proc. Natl. Acad. Sci. USA* *110*, 3298–3303 (2013).

8. Whittle, A., Bartosiewicz, L., Borić, D., Pettitt, P. & Richards, M. In the beginning: new radiocarbon dates for the Early Neolithic in northern Serbia and south-east Hungary. *Antaeus* **25**, 63–117 (2002).

9. Quitta, H. Die Radiocarbondaten und ihre historische Interpretation in *Lepenski Vir. Eine vorgeschichtliche Geburtsstätte europäischer Kultur* (ed. Srejović, D.) 272–285 (Gustav Lübbe Verlag, 1975).

10. Bonsall, C. *et al.* Mesolithic and early Neolithic in the Iron Gates: a palaeodietary perspective. *J. of European Archaeology* **5**(1), 50–92 (1997).

11. Stafford, T., Jull, A. J. T., Brendel, K., Duhamel, R. & Donahue, D. Study of bone radiocarbon dating accuracy at the University of Arizona NSF accelerator facility for radioisotope analysis. *Radiocarbon* **29**(1), 24–44 (1987).

12. Kohl, G. & Müller, H. Berlin radiocarbon measurements III. *Radiocarbon* **11**(2), 271–277 (1969).

13. Barker, H. Radiocarbon dating: large-scale preparation of acetylene from organic material. *Nature* **172**, 631–632 (1953).

14. Barker, H. & MacKey, C. J. British Museum natural radiocarbon measurements V. *Radiocarbon* 10, 1–7 (1968).

15. Barker, H. & MacKey, C. J. British Museum natural radiocarbon measurements I. *American Journal of Science Radiocarbon* supplement **1**, 81–86 (1959).

16. Gillespie, R., Hedges, R. & White, N. R. The Oxford radiocarbon accelerator facility. *Radiocarbon* **25**, 729–737 (1983).

17. Hedges, R. Radiocarbon dating with an accelerator: review and preview. *Archaeometry* **23**, 1–18 (1981).

18. Hedges, R. & Law, I. A. The radiocarbon dating of bone. *Applied Geochemistry* **4**, 249–253 (1989).

19. Law, I. A. & Hedges, R. E. M. A semi-automated pre-treatment system and the pretreatment of older and contaminated samples. *Radiocarbon* **31**, 247–253 (1989).

20. Bronk Ramsey, C., Higham, T., Bowles, A. & Hedges, R. E. M. Improvements to the pre-treatment of bone at Oxford. *Radiocarbon* **46**, 155–163 (2004).

21. Brock, F., Bronk Ramsey, C. & Higham, T. Quality assurance of ultrafiltered bone dating. *Radiocarbon* **49**(2), 187–192 (2007).

22. Brock, F., Higham, T. & Bronk Ramsey, C. Pre-screening techniques for identification of samples suitable for radiocarbon dating of poorly preserved bones. *J. of Archaeol. Sci.* **37**, 855–865 (2010).

23. Dee, M. & Bronk Ramsey, C. Refinement of the graphite target production at ORAU. *Nuclear Instruments and Methods in Physics Research B* **172**, 449–453.

24. Bronk Ramsey, C., Higham, T. & Leach, P. Towards high precision AMS: progress and limitations. *Radiocarbon* **46**, 17–24 (2004).

25. Srdoč, D., Breyer, B. & Sliepčević, A. Ruđer Bošković Institute radiocarbon measurements I. *Radiocarbon* **13**(1), 135–140 (1971).

26. Srdoč, D., Sliepčević, A. & Planinic, J. Ruđer Bošković Institute radiocarbon measurements III. *Radiocarbon* **17**(1), 149–155 (1975).

27. Milner, N., Craig, O. E., Bailey, G. N., Pederson, K. & Anderson, S. H. Something fishy in the Neolithic? A re-evaluation of stable isotope analysis of Mesolithic and Neolithic costal populations. *Antiquity* **78**, 9–22 (2004).

28. Evershed, R. P. Exploiting molecular and isotopic signals at the Mesolithic-Neolithic transition. *Proceedings of the British Academy* **144**, 141–64 (2007).

29. van der Sluis, L. G. *et al*. Combining histology, stable isotope analysis and ZooMS collagen fingerprinting to investigate the taphonomic history and dietary behaviour of extinct giant tortoises from the Mare aux Songes deposit on Mauritius. *Palaeogeography, Palaeoclimatology, Palaeoecology* **416**, 80–91 (2014).

30. Buckley, M., Harvey, V. L. & Chamberlain, A. T. Species identification and decay assessment of Late Pleistocene fragmentary vertebrate remains from Pin Hole Cave (Creswell Crags, UK) using collagen fingerprinting. *Boreas* **46**(3), 402–411 (2017).

31. Buckley, M. Species identification of bovine, ovine and porcine type 1 collagen; comparing peptide mass fingerprinting and LC-based proteomics methods. *International Journal of Molecular Sciences*, **17**(4), 445 (2016).

32. Buck, C., Cavanagh, W. G. & Litton, C. *Bayesian Approach to Interpreting Archaeological Data* (Wiley, 1996).

33. Bayliss, A., Bronk Ramsey, C., van der Plicht, J. & Whittle, A. Bradshaw and Bayes: towards a timetable for the Neolithic. *Cambridge Archaeol. J.* **17.1**, supplement, 1–28 (2007).

1. 1 Low pretreatment yield of collagen: 590 mg was treated and 4.14 mg recovered, a yield of 0.7% wt. collagen, and below the Oxford Laboratory’s minimum threshold. [↑](#footnote-ref-1)
